# Correction: Multi-methodological approach for the Quality assessment of Senecionis scandentis Herba (Qianliguang) in the herbal market

**DOI:** 10.1371/journal.pone.0329396

**Published:** 2025-07-29

**Authors:** Hiu-Lam Ngai, Xiao Yang, Adrian Jun Chu, Rachel Harper, Alice B. J. E. Jacobsen, David Tai-Wai Lau, Ho-Yin Yu, Hung-Kay Lee, Pang-Chui Shaw

## Notice of Republication

This article was republished on July 10, 2025, to correct errors in Table 1, Figure 1, and S8 File that were introduced during the publication process. Please download this article again to view the correct version. The originally published, uncorrected article and the republished, corrected articles are provided here for reference.

## Supporting information

S1 File Originally published, uncorrected article. (PDF)

S2 File Republished, corrected article. (PDF)
