# Supplementary material for: Multi-methodological approach for the Quality assessment of Senecionis scandentis Herba (Qianliguang) in the herbal market
Source: PLoS One. 2022 Apr 14;17(4):e0267143. doi: 10.1371/journal.pone.0267143 (PMC9009707; doi:10.1371/journal.pone.0267143)
Supplement: S1 File — (PDF) [file pone.0267143.s001.pdf]

S1 File. A summary of organoleptic characteristics and morphological identities

1a. Organoleptic characteristics of Senecionis scandentis Herba

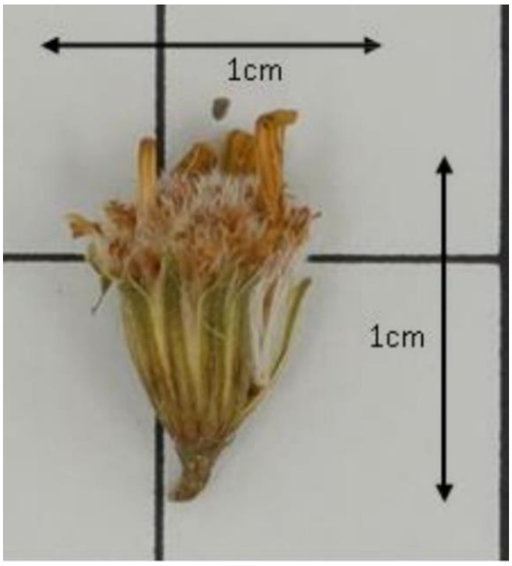

S1 Fig. Heterogamous capitula radiate; tubular flowers numerous (7.5 mm long); corolla yellow.

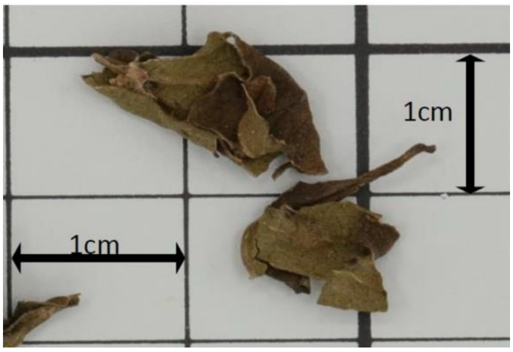

S2 Fig. Leaf veins are pinnate. Both surfaces of leaves usually appear pilose.

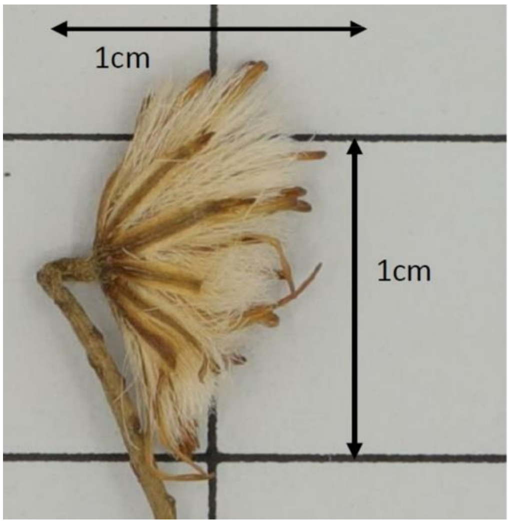

S3 Fig. Peduncles are often reflexed or extended and covered in densely minute hairs, with thinly striated bracts.

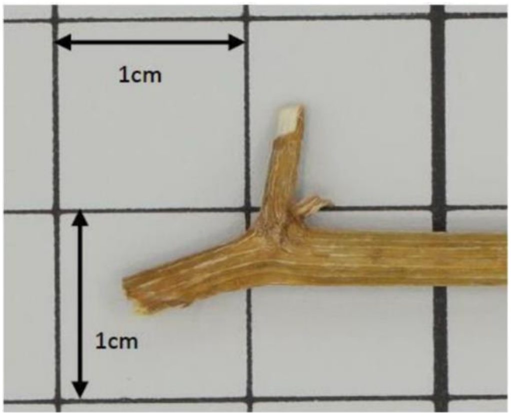

S4 Fig. Stem surface is a grayish-green, yellowish-brown, or purplish-brown color. Stem has longitudinal ridges.

1b. Organoleptic characteristics of adulterant of species belonging to the genus *Lespedeza*

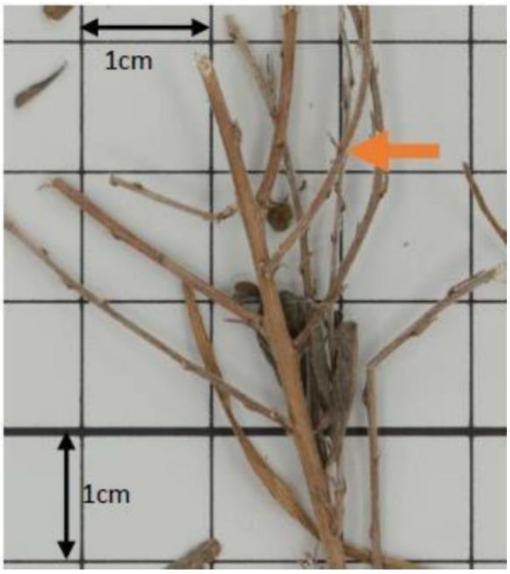

S5 Fig. Woody and fibrous stems, with sharp, stiff, flattened bristles.

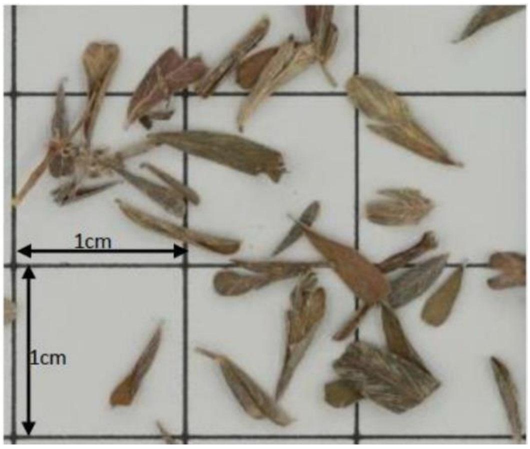

S6 Fig. Dense, narrowly oblong and pointed leaflets, with wedge-shaped base covered in hairs; grayish-green or silvery in color.

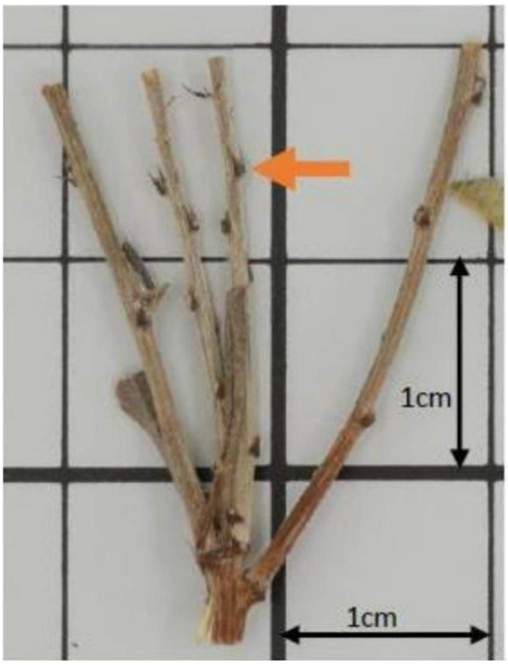

S7 Fig. Awl-shaped spines are present on the stems, indicated by the arrow.

1c. Organoleptic characteristics of adulterant *Achyranthes aspera* Linnaeus

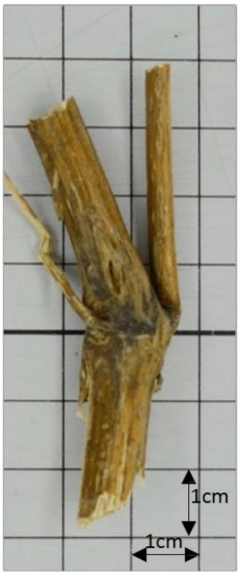

S8 Fig. Radical are stout, cylindrical in shape, in khaki color.

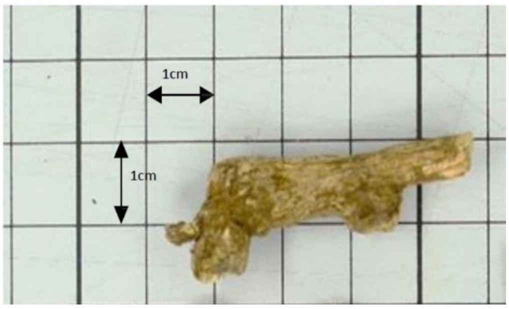

S9 Fig. Stem nodes are slightly enlarged and knee-like.

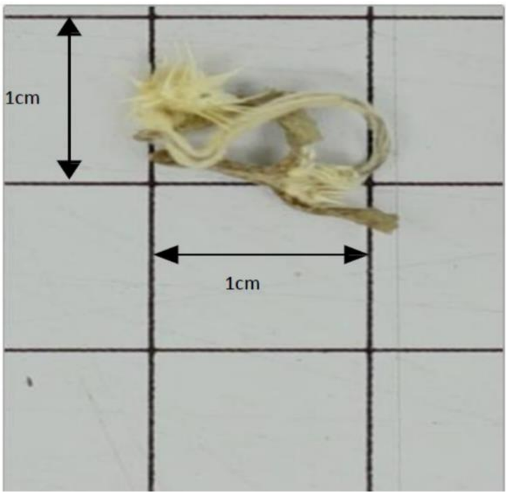

S10 Fig. Flowers are pseudo-staminodes with margins fimbriate at apex.

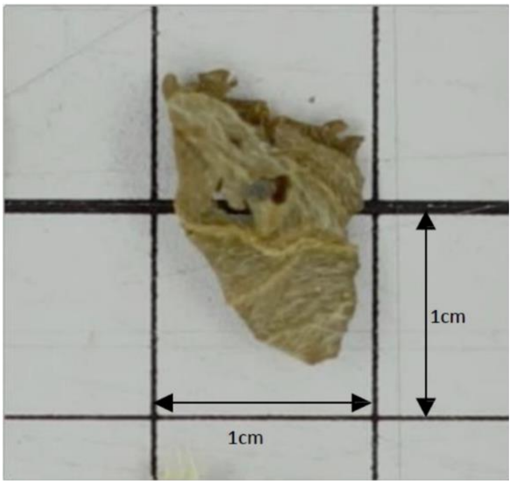

S11 Fig. Leaf 1-20 × 2-6 cm; adpressed-pubescent abaxially and adaxially.

2. Images and major morphological identities of each sample

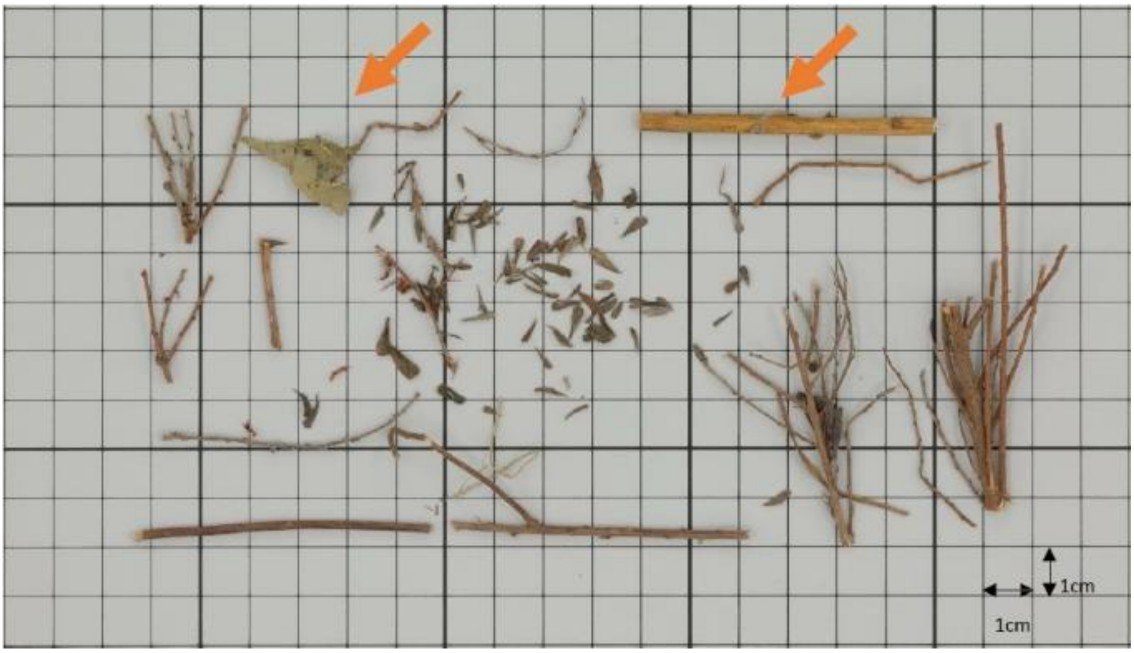

S12 Fig. Sample T5060 obtained from Shatin was authenticated as species belonging to the genus *Lespedeza*, with a few unidentified fragments, indicated by arrows.

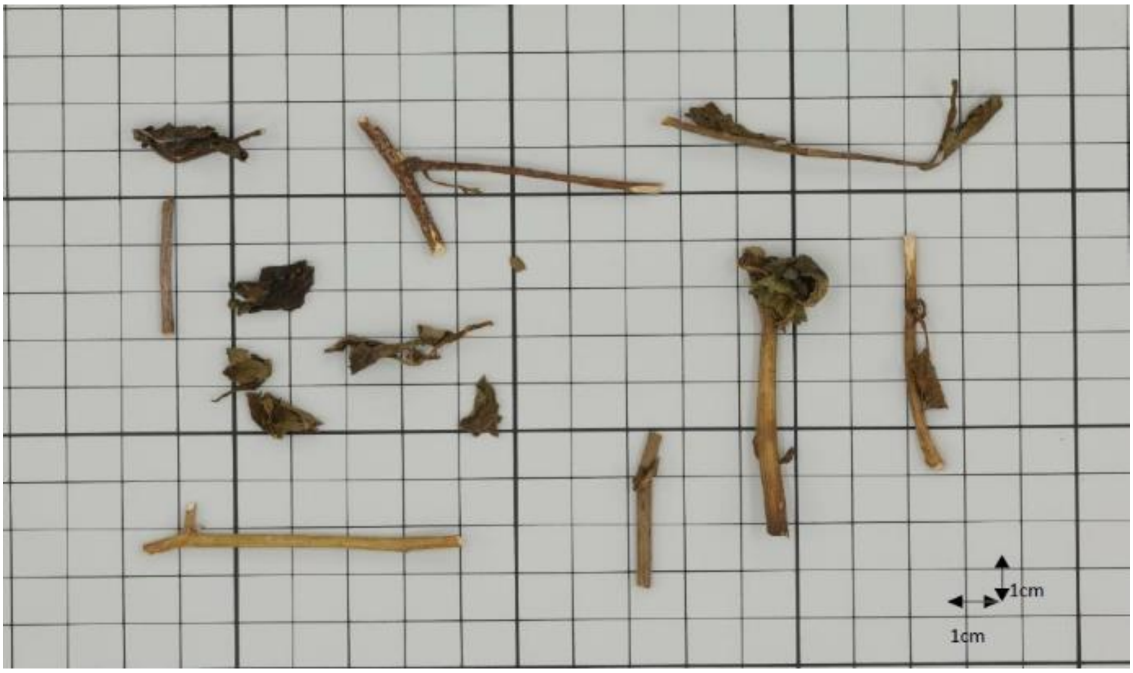

S13 Fig. Sample T5061 was obtained from Tsuen Wan. Its major authentication result was Senecionis scandentis Herba.

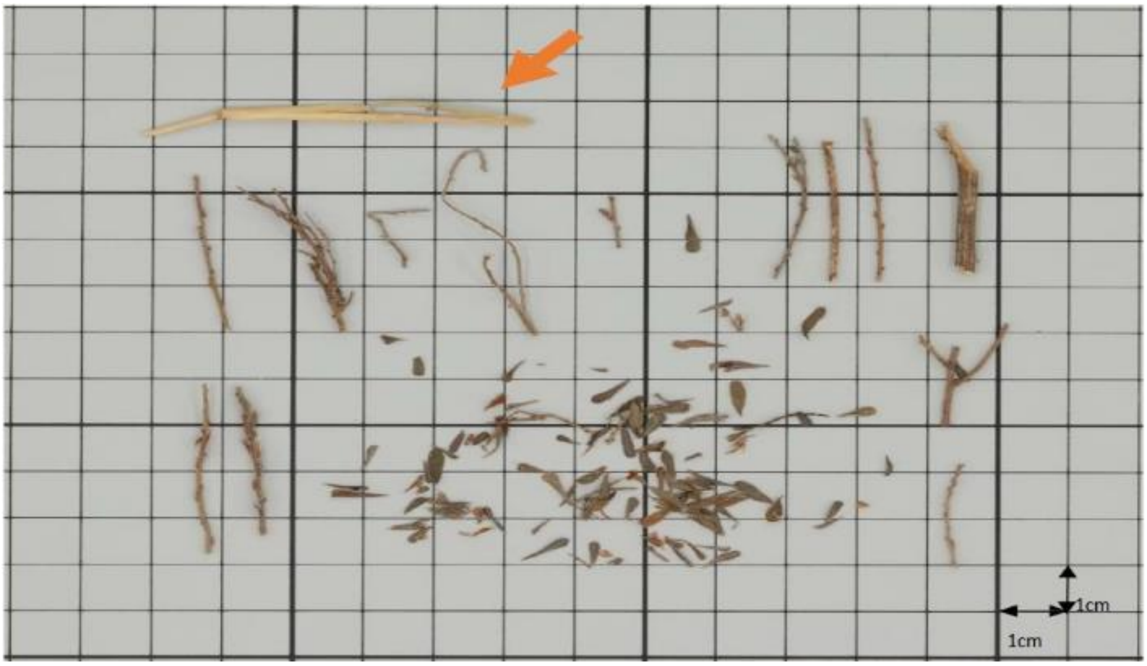

S14 Fig. Sample T5062 was obtained from Sheung Shui. It was authenticated as species belonging to the genus *Lespedeza*, with a few unidentified fragments indicated by the arrow.

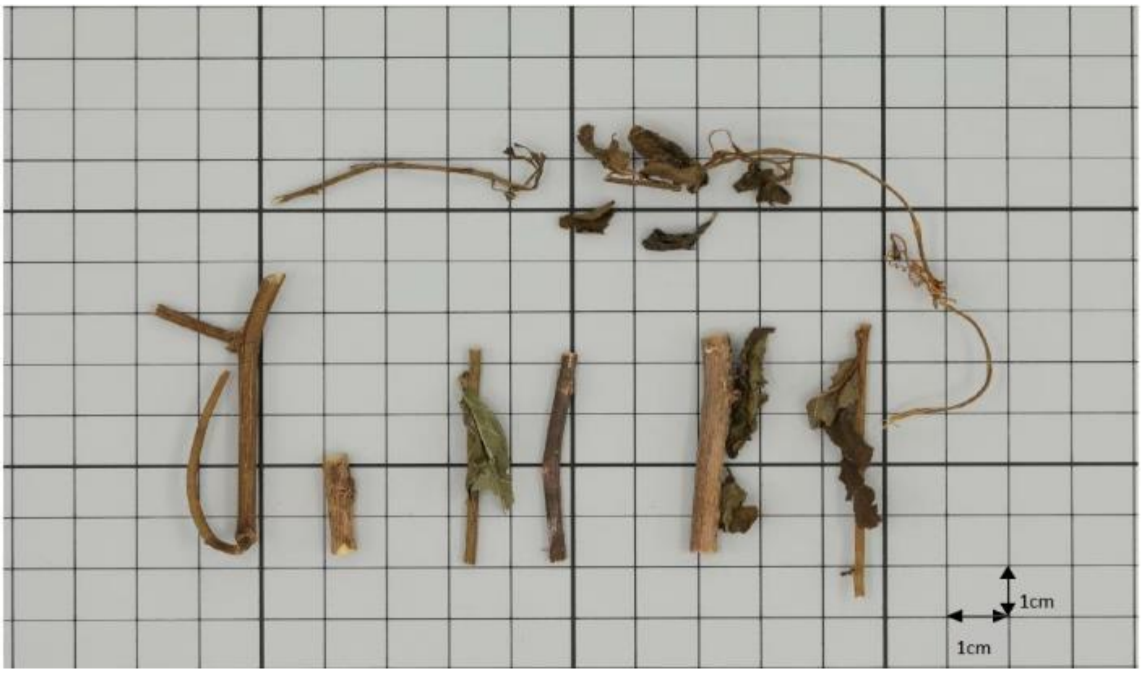

S15 Fig. Sample T5063 was obtained from Sham Shui Po. Its major authentication result was Senecionis scandentis Herba.

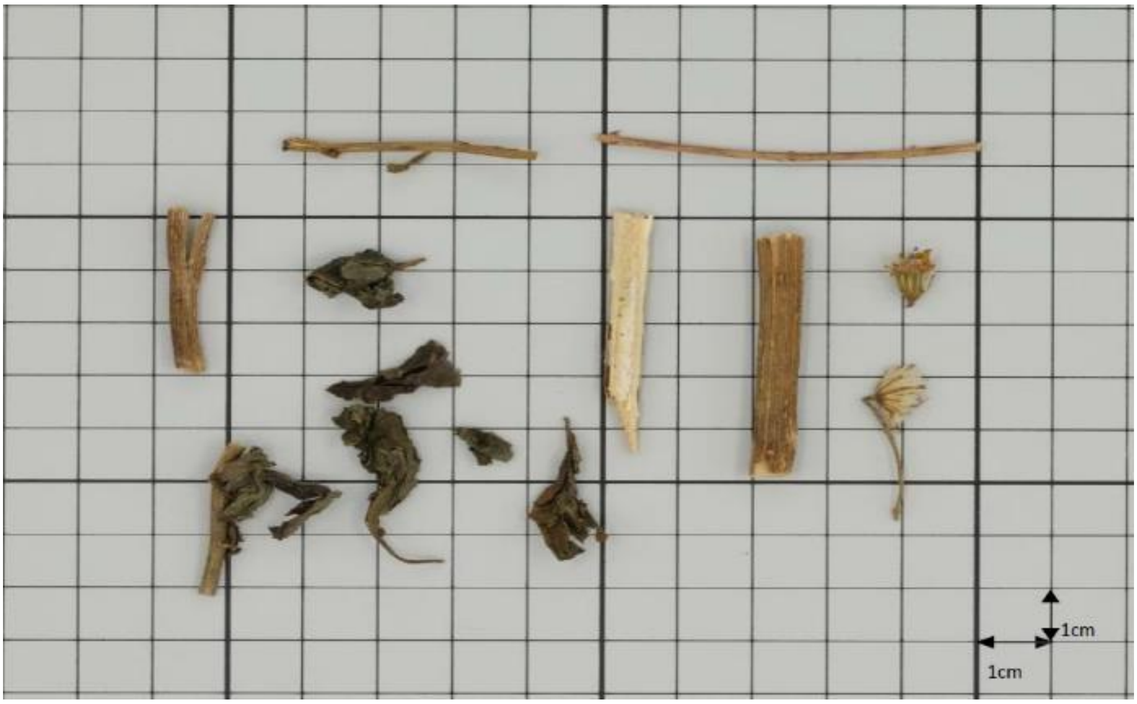

S16 Fig. Sample T5064 was obtained from Wong Tai Sin. Its major authentication result was Senecionis scandentis Herba.

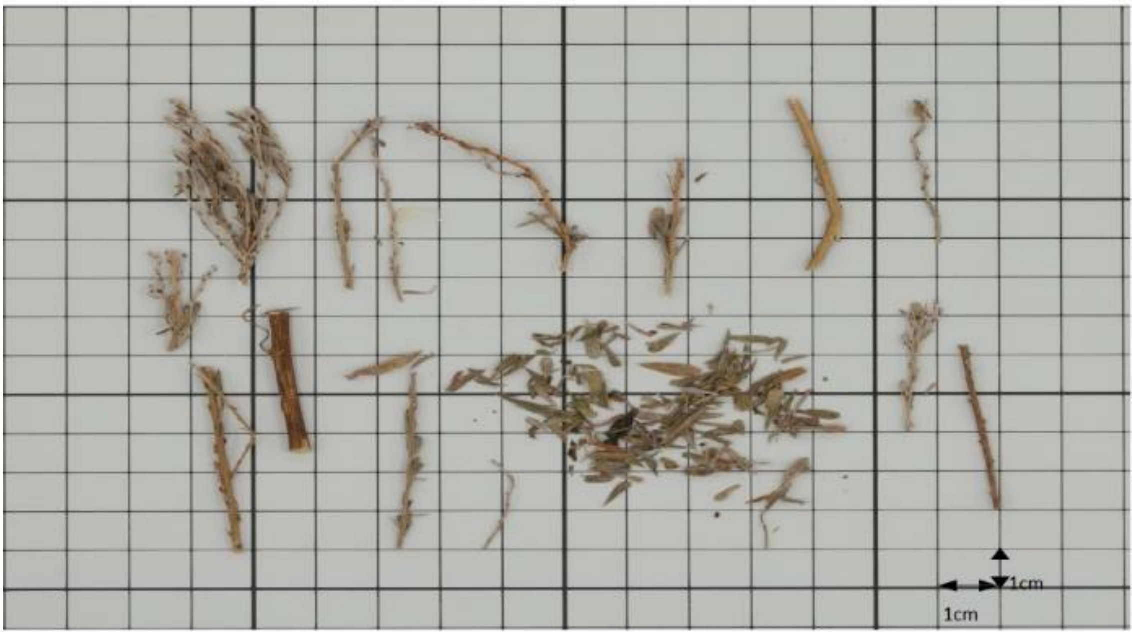

S17 Fig. Sample T5079 was obtained from Sheung Wan. Its major authentication result was species belonging to the genus *Lespedeza*.

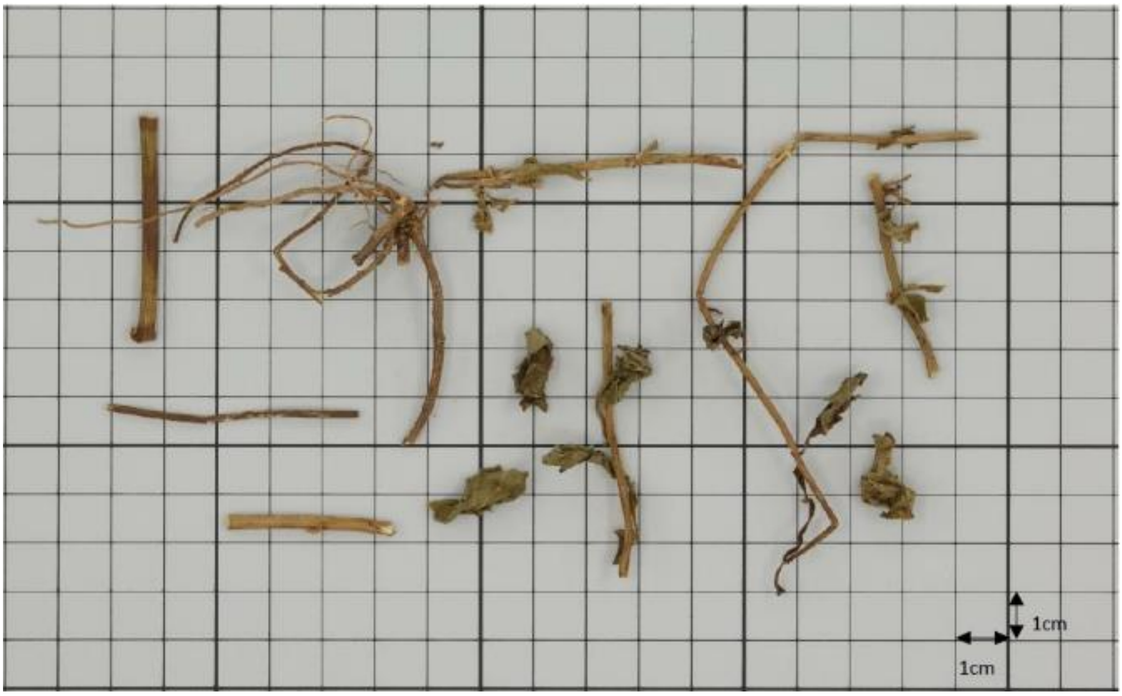

S18 Fig. Sample T5135 was obtained from Kwun Tong. Its major authentication result was Senecionis scandentis Herba.

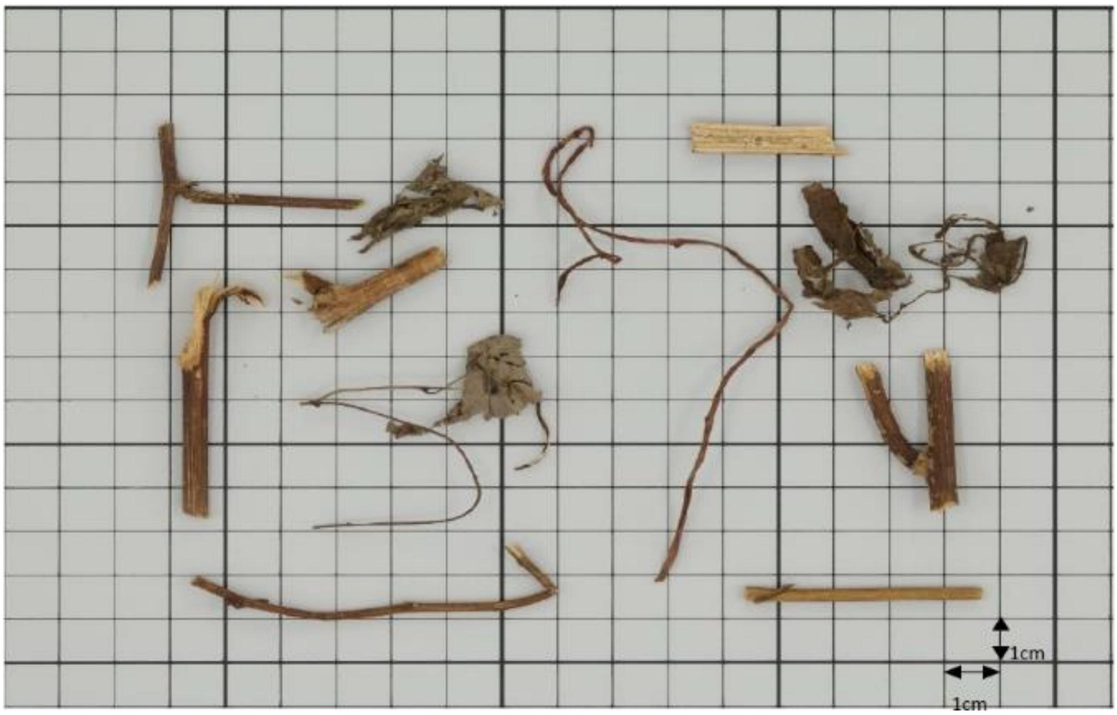

S19 Fig. Sample T5138 was obtained from To Kwa Wan. Its major authentication result was Senecionis scandentis Herba.

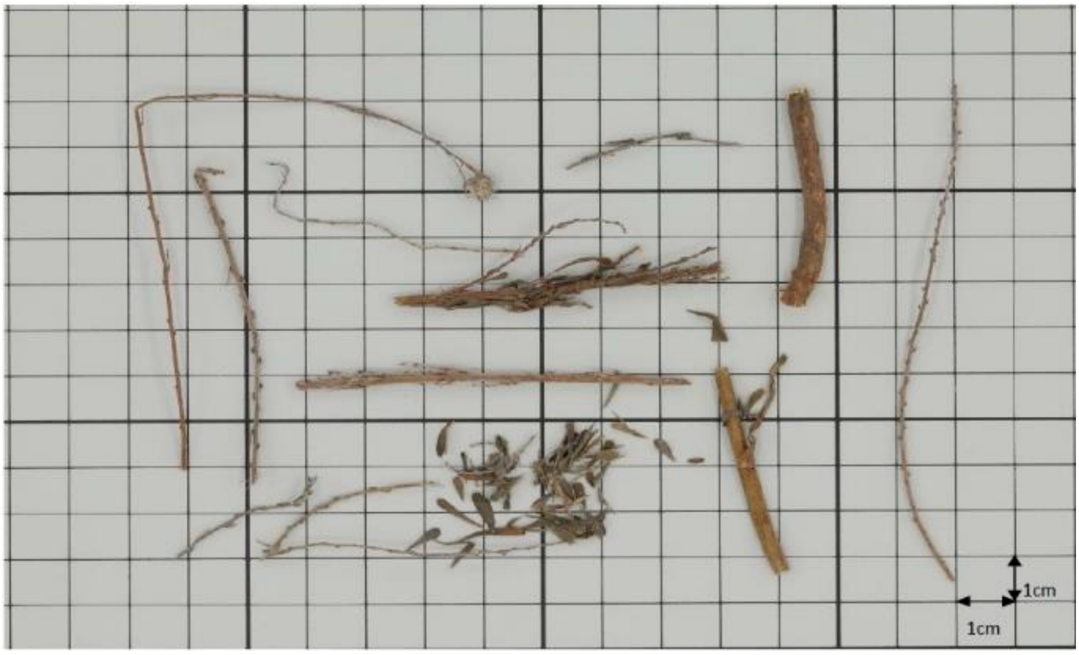

S20 Fig. Sample T5141 was obtained from Wan Chai. Its major authentication result was species belonging to the genus *Lespedeza*.

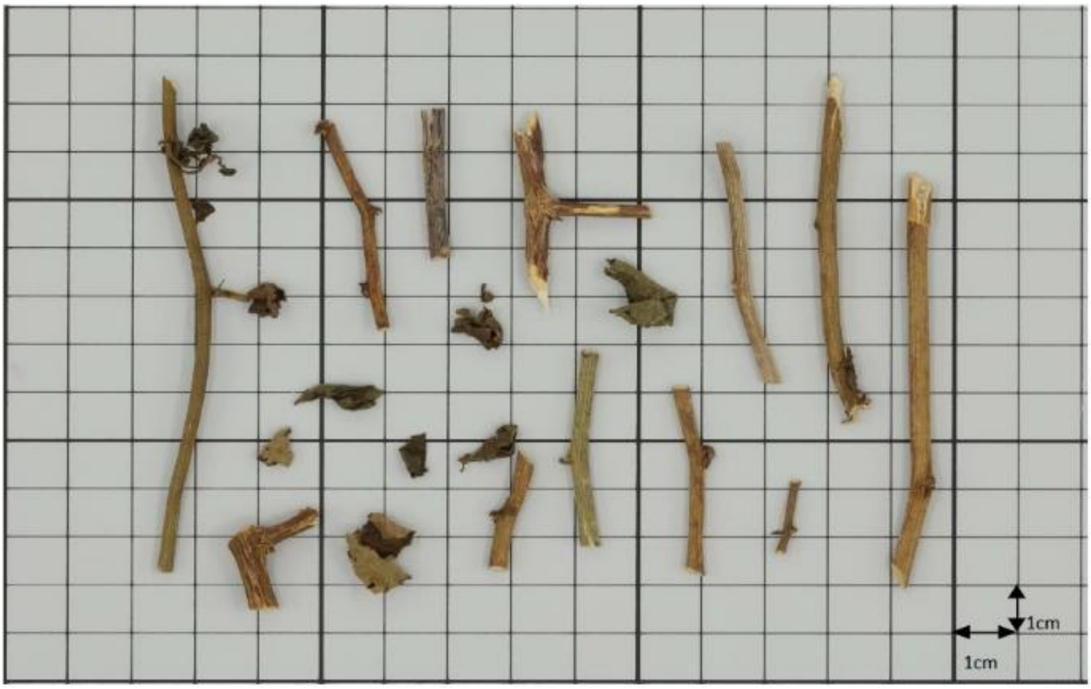

S21 Fig. Sample T5144 was obtained from North Point. Its major authentication result was Senecionis scandentis Herba.

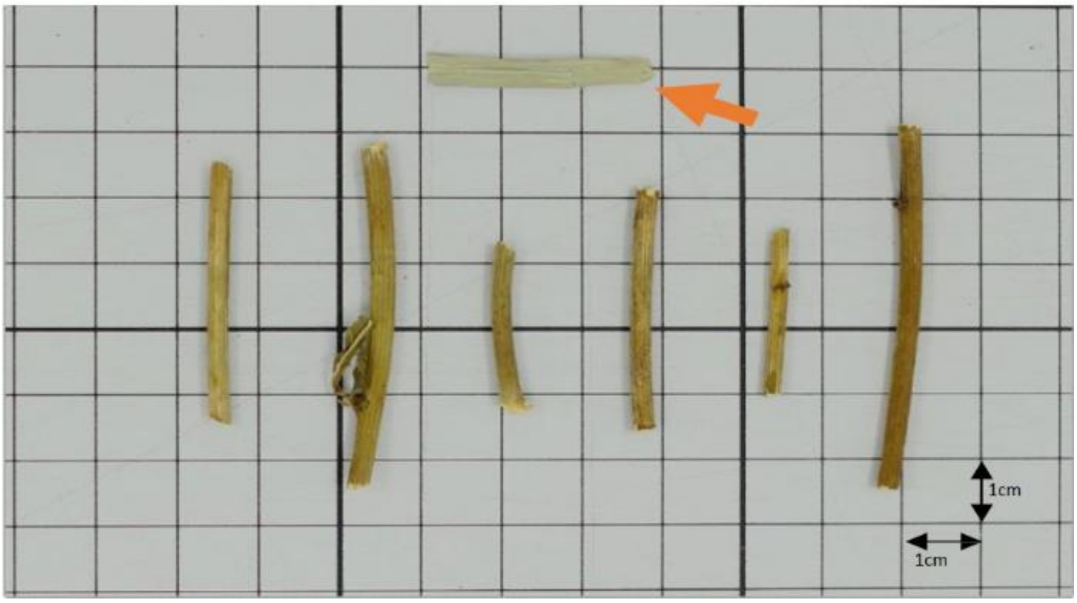

S22 Fig. Sample T5387 was obtained from Tai Po. Its major authentication result was Senecionis scandentis Herba, with a few unidentified species indicated by the arrow.

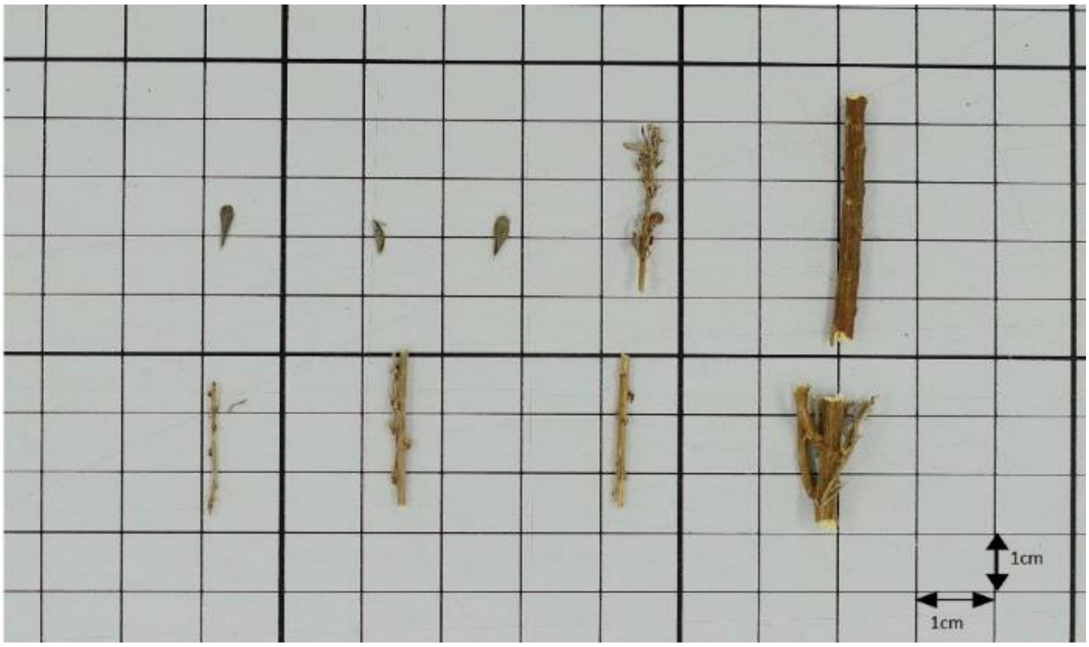

S23 Fig. Sample T5388 was obtained from Jordan. Its major authentication result was species belonging to the genus *Lespedeza*.

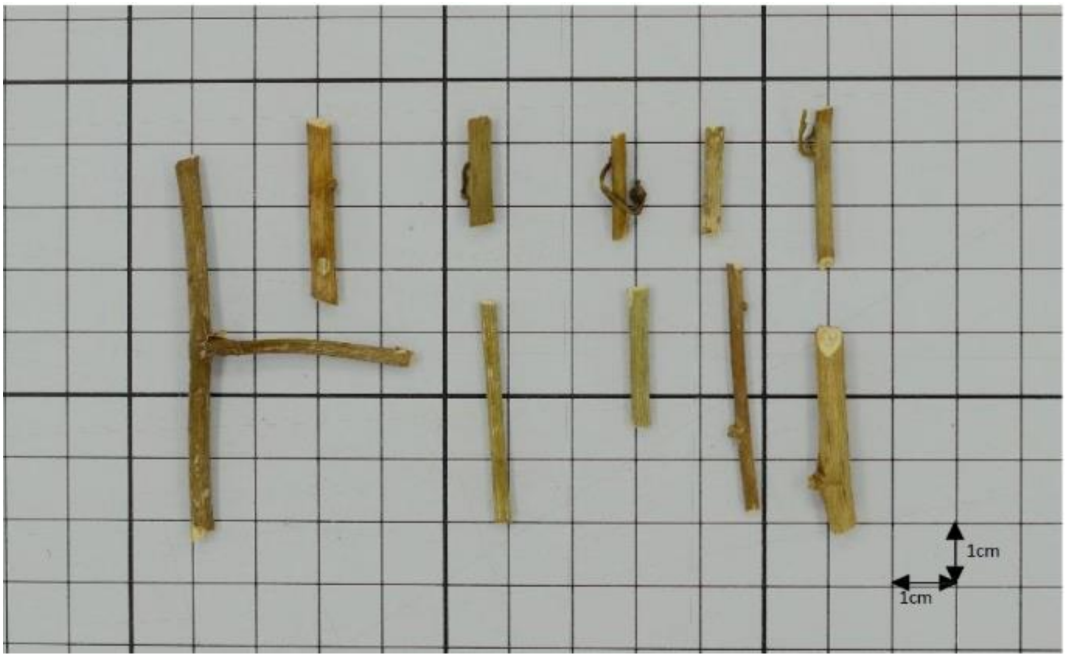

S24 Fig. Sample T5389 was obtained from Yuen Long. Its major authentication result was Senecionis scandentis Herba.

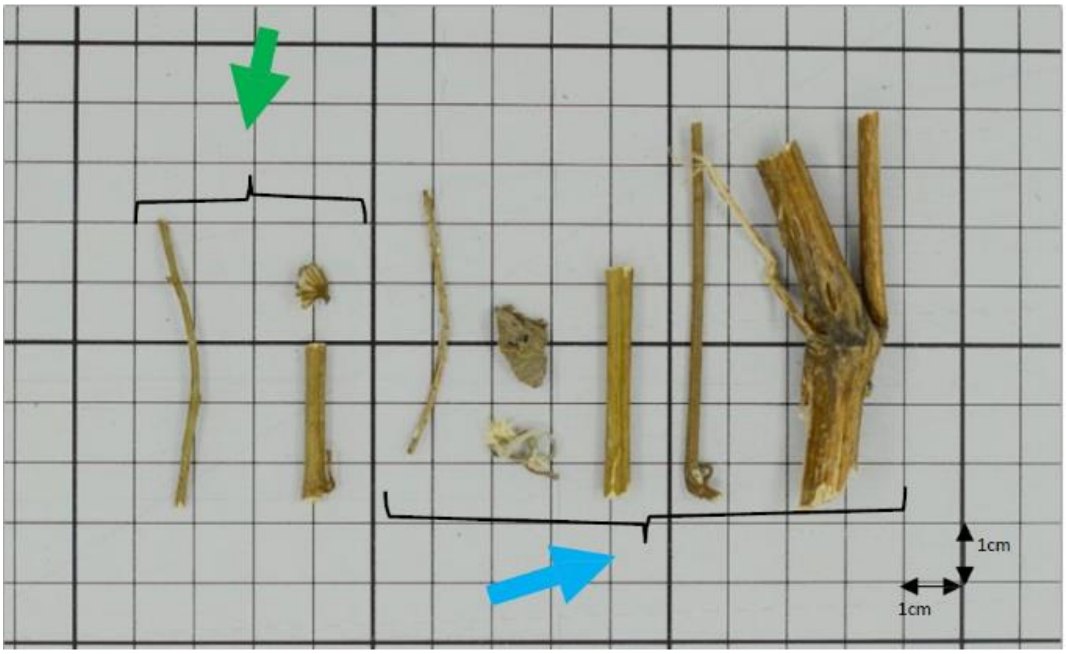

S25 Fig. Sample T5390 was obtained from Tuen Mun. Its major authentication results included *Achyranthes aspera* Linnaeus indicated by blue arrow; and Senecionis scandentis Herba, indicated by the green arrow.

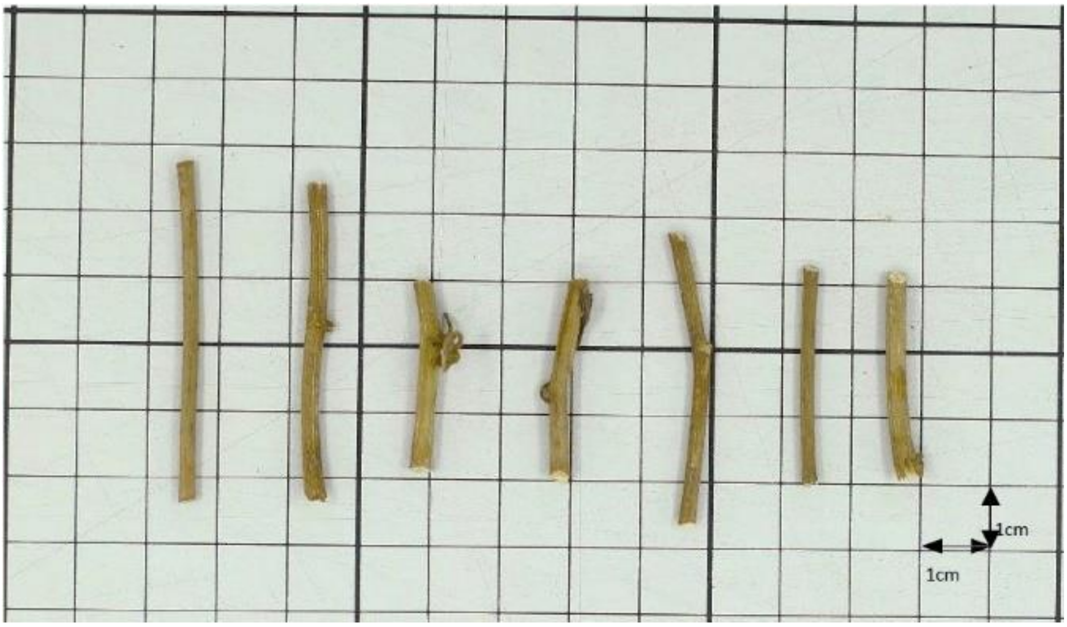

S26 Fig. Sample T5391 was obtained from Tsing Yi. Its major authentication result was Senecionis scandentis Herba.

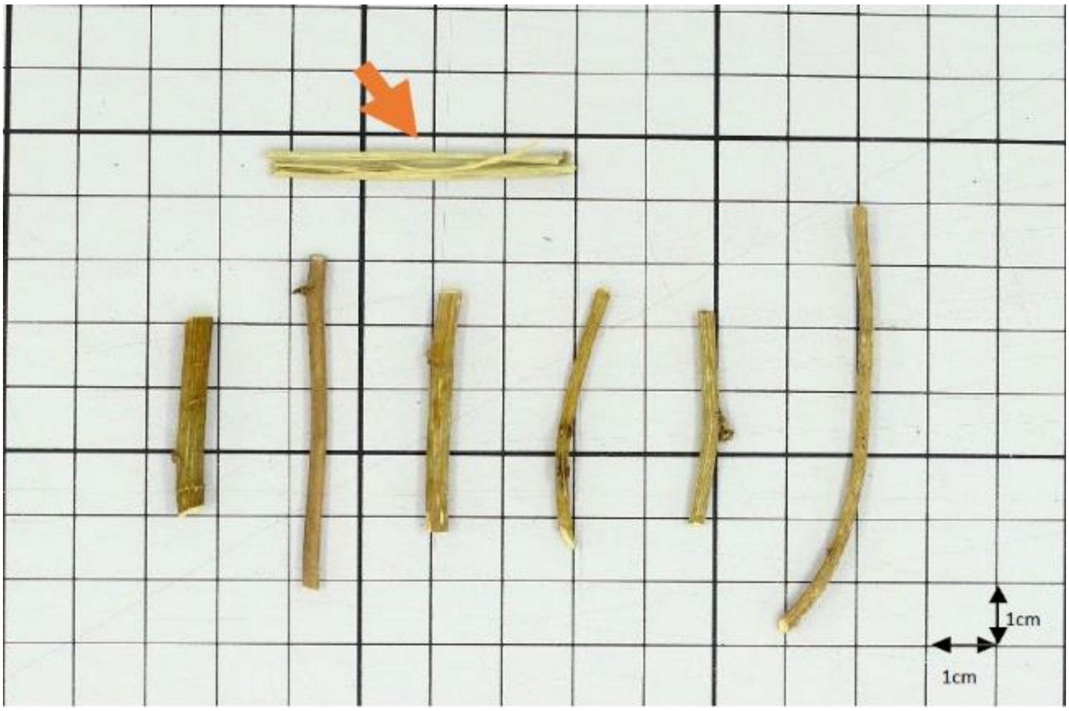

S27 Fig. Sample T5392 was obtained from Sai Kung. Its major authentication result was Senecionis scandentis Herba, with a few unidentified species indicated by the arrow.

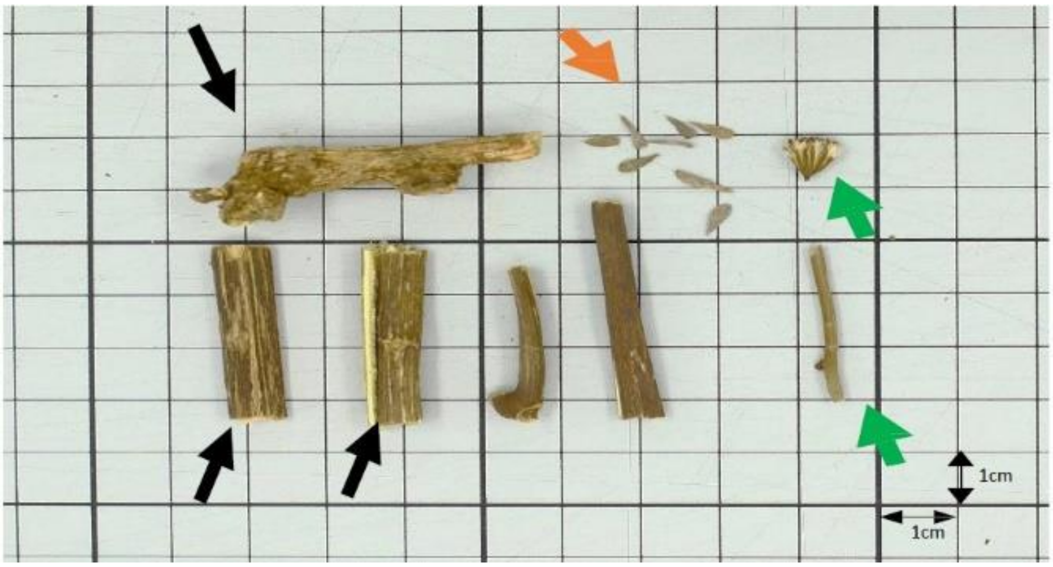

S28 Fig. Sample T5393 was obtained from South District. Its major authentication result was *Achyranthes aspera* Linnaeus indicated by the black arrows; Senecionis scandentis Herba indicated by the green arrows, and a few unidentified species indicated by the orange arrow.

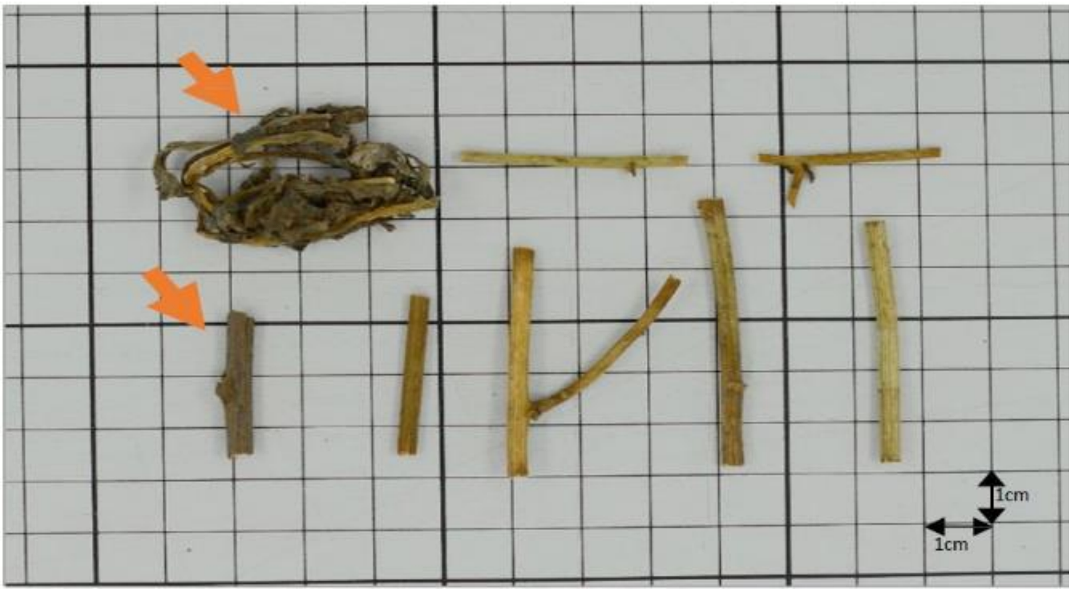

S29 Fig. Sample T5394 was obtained from Tung Chung. Its major authentication result was Senecionis scandentis Herba, with a few unidentified species indicated by the orange arrows.
