# Supplementary material for: Multi-methodological approach for the Quality assessment of Senecionis scandentis Herba (Qianliguang) in the herbal market
Source: PLoS One. 2022 Apr 14;17(4):e0267143. doi: 10.1371/journal.pone.0267143 (PMC9009707; doi:10.1371/journal.pone.0267143)
Supplement: S2 File — (PDF) [file pone.0267143.s002.pdf]

S2 File. PCR amplification protocols

Primer	Primer name	Primer sequence (5’ to 3’)	PCR protocol	DNA locus
rbcL1/rbcLB	rbcL1<br><br>rbcLB	TTG GCA GCA TTY CGA GTA ACT CC<br><br>AAC CYT CTT CAA AAA GGT C	Initial denaturation:<br>95 °C for 2.5 min<br>10 cycles of<br>[30 s at 95 °C denaturation,30 s at 52 °C annealing, 30 s at 72 °C extension]<br>25 cycles of<br>[30 s at 88 °C denaturation, 30 s at 52 °C annealing, 30 s at 72 °C extension] Final extension:<br>60 °C for 10 min<br>Enzyme deactivation:<br>4 °C for 5 min	*rbcL*
ITSp3/ITSu4<br>ITSu3/ITSu4	ITSp3<br><br>ITSu3<br><br>ITSu4	YGA CTC TCG GCA ACG GAT A<br><br>CAW CGA TGA AGA ACG YAG C<br><br>RGT TTC TTT TCC TCC GCT TA	Initial Denaturation:<br>94 °C for 4 min<br>40 cycles of<br>[30 s at 94 °C denaturation, 40 s at 50 °C annealing, 1 min at 72 °C extension] Final extension:<br>72 °C for 10 min<br>Enzyme deactivation:<br>4 °C for 5 min	*ITS2*

Species specific primers *ITS2*	SSP-F<br><br>SSP-R	ACG ATT AGT GGT GGT TGT CAA G<br><br>TAA ACT CAG CGG GTA GTC C	Initial Denaturation:<br>94◦C for 10 s<br>40 cycles of<br>[20 s at 94◦C denaturation, 20 s at 58◦C annealing, 20 s for 72◦C extension]<br>Final extension:<br>72◦C for 20 s<br>Enzyme deactivation:<br>4 °C for 5 min	
psbAF/trnHR	psbAF<br><br>trnHR	GTT ATG CAT GAA CGT AAT GCT C<br><br>CGC GCA TGG TGG ATT CAC AAT CC	Initial Denaturation:<br>94 °C for 5 min<br>30 cycles of<br>[1 min at 94 °C denaturation, 1 min at 56 °C annealing, 1.5 min at 72 °C extension]<br>Final extension:<br>72 °C for 7 min<br>Enzyme deactivation:<br>4 °C for 5 min	*psbA-trnH*
