# Supplementary material for: Multi-methodological approach for the Quality assessment of Senecionis scandentis Herba (Qianliguang) in the herbal market
Source: PLoS One. 2022 Apr 14;17(4):e0267143. doi: 10.1371/journal.pone.0267143 (PMC9009707; doi:10.1371/journal.pone.0267143)
Supplement: S3 File — (PDF) [file pone.0267143.s003.pdf]

S3 File. Species-specific primer design

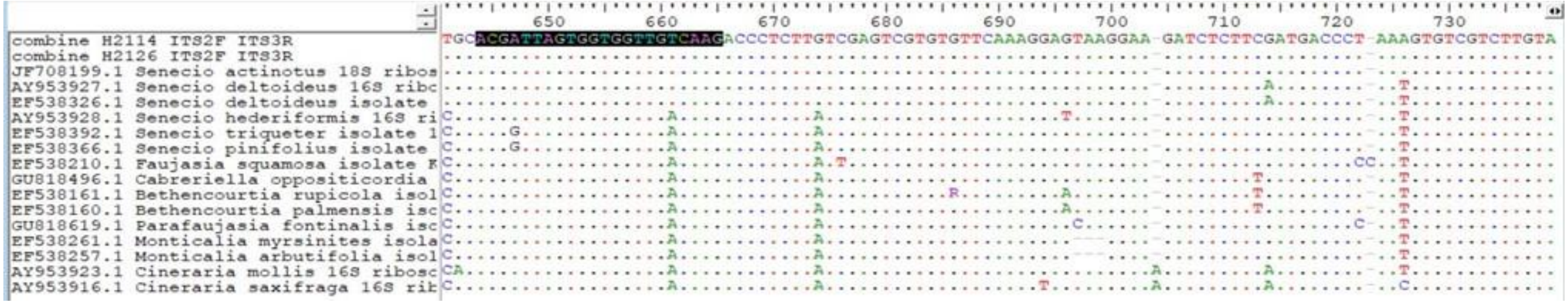

S30 Fig. Sequence alignment. Forward primer highlighted in black color. The first two rows are the sequences of biological standards Senecio scandens coded H2114 and H2126. The sequences below were downloaded from GenBank and with highest similarity. They were used for sequence alignment and primer design. Major differences between standards and other species can be identified at positions 661, 674, and 726.

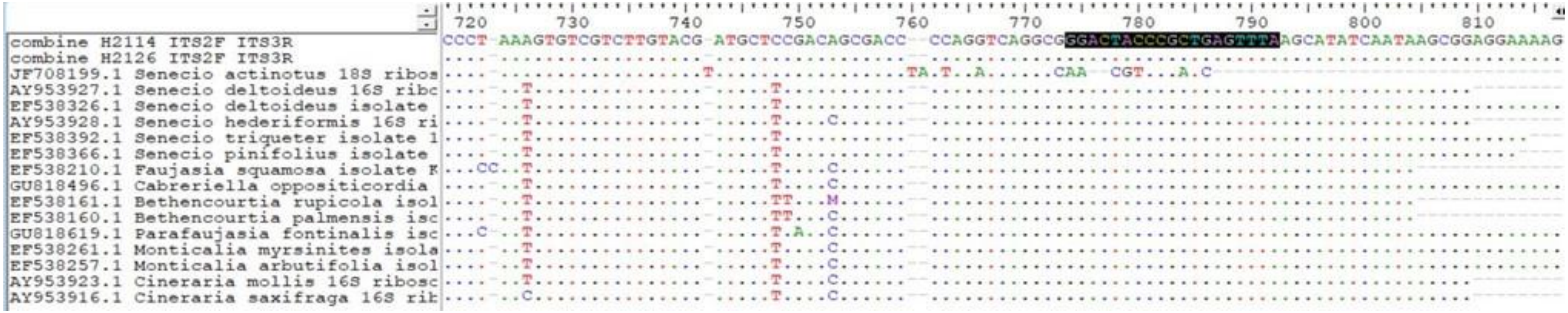

S31 Fig. Sequence alignment. Sequence where reverse primer is derived from is highlighted in black color. The differences between sequences of biological standards in rows 1 and 2, and sequence of Senecio actinotus in row 3 can be identified by the differences of A, T, C and G.
