# Supplementary material for: Multi-methodological approach for the Quality assessment of Senecionis scandentis Herba (Qianliguang) in the herbal market
Source: PLoS One. 2022 Apr 14;17(4):e0267143. doi: 10.1371/journal.pone.0267143 (PMC9009707; doi:10.1371/journal.pone.0267143)
Supplement: S4 File — (PDF) [file pone.0267143.s004.pdf]

S4 File. Sequence alignment at *ITS2*, *psbA-trnH*, and *rbcL* DNA regions

1a. Sequence alignment at the *ITS2* region (primer ITSp3/ITSsu4)

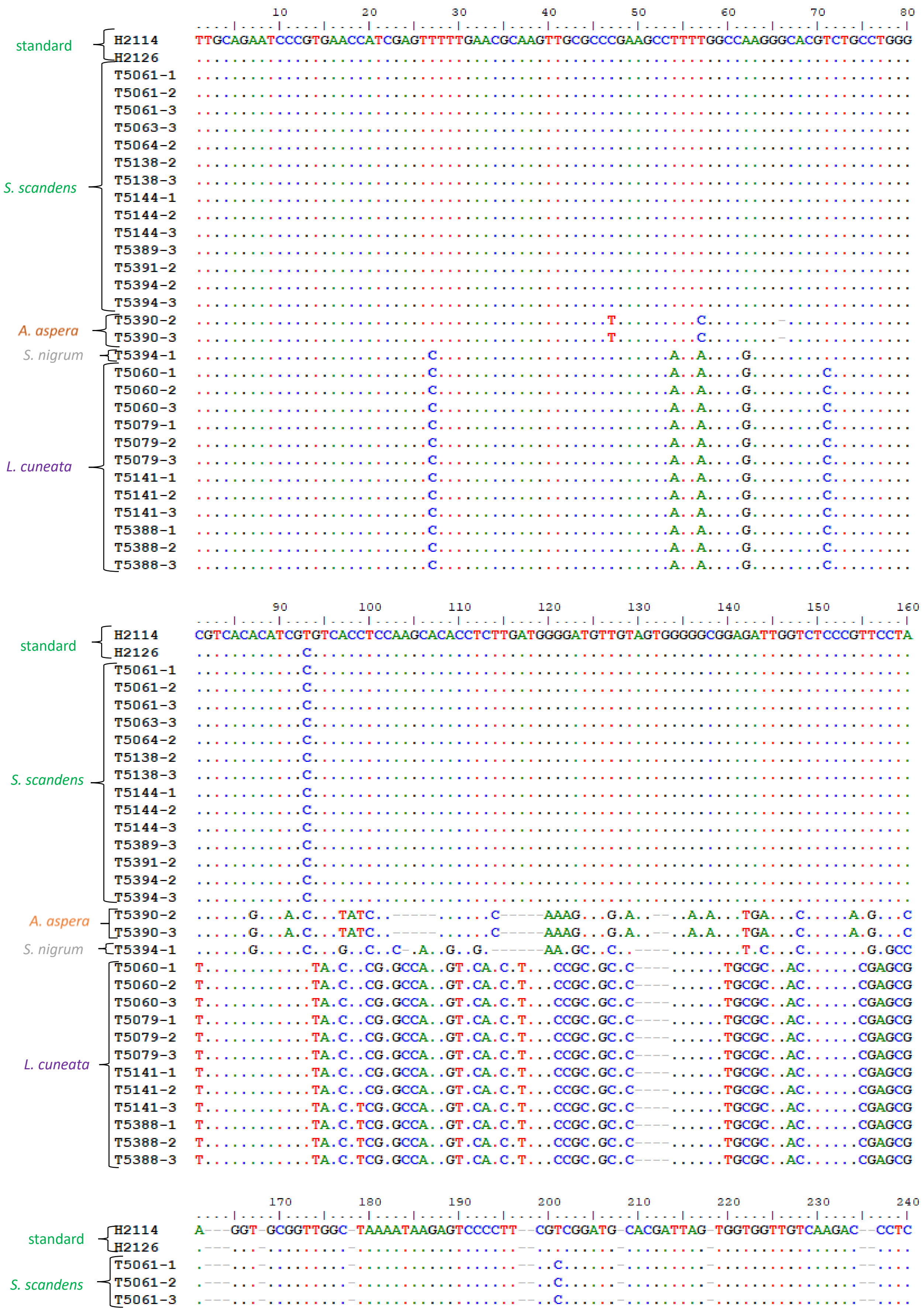

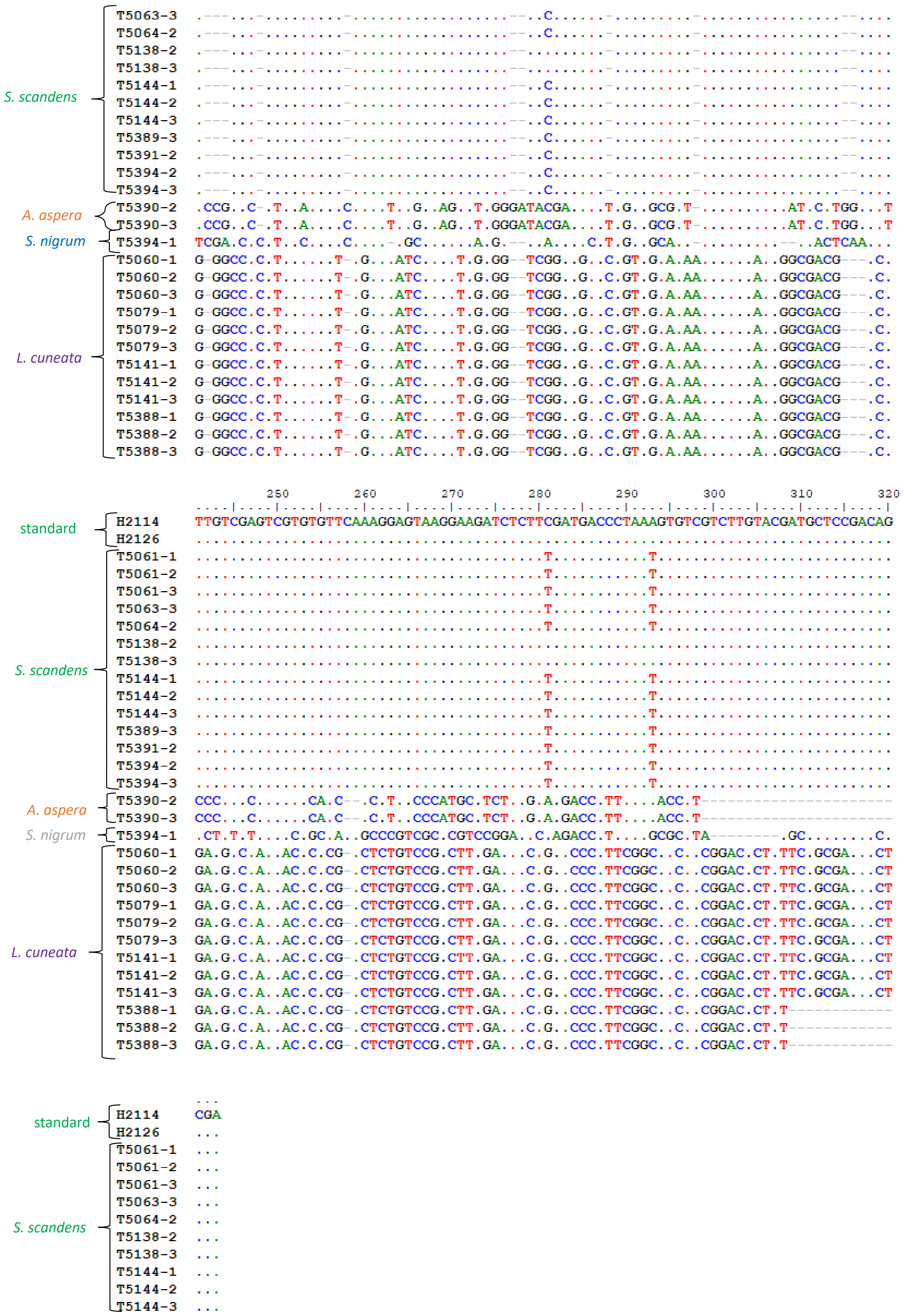
S. scandens
A. aspera
S. nigrum
L. cuneata
standard
H2114
H2126
T5060-1
T5060-2
T5060-3
T5061-1
T5061-2
T5061-3
T5063-3
T5064-2
T5079-1
T5079-2
T5079-3
T5138-2
T5138-3
T5141-1
T5141-2
T5141-3
T5144-1
T5144-2
T5144-3
T5388-1
T5388-2
T5388-3
T5389-3
T5390-2
T5390-3
T5391-2
T5394-1
T5394-2
T5394-3
250
260
270
280
290
300
310
320
TTGTCGAGTCGTGTGTTCAAAGGAGTAAGGAAGATCTCTTCGATGACCCTAAAGTGTCGTCTTGTACGATGCTCCGACAG
CGA

1b. Sequence alignment at the *ITS2* region (primer ITSu3/ITSsu4)

```
                            10        20        30        40        50        60        70        80
                    ....|....|....|....|....|....|....|....|....|....|....|....|....|....|....|....|
standard     H2114  CCATCGAGTTTTTGAACGCAAGTTGCGCCCGAAGCCTTTTGGCCAAGGGCACGTCTGCCTGGGCGTCACACATCGTGTCA
             H2126  ...........................................................................C....
S. scandens  T5064-1 ...........................................................................C....
             T5135-1 ...........................................................................C....
             T5135-2 ...........................................................................C....
             T5135-3 ...........................................................................C....
             T5392-1 ...........................................................................C....

                            90       100       110       120       130       140       150       160
                    ....|....|....|....|....|....|....|....|....|....|....|....|....|....|....|....|
standard     H2114  CCTCCAAGCACACCTCTTGATGGGGATGTTGTAGTGGGGGCGGAGATTGGTCTCCCGTTCCTAAGGTGCGGTTGGCTAAA
             H2126  ................................................................................
S. scandens  T5064-1 ................................................................................
             T5135-1 ................................................................................
             T5135-2 ................................................................................
             T5135-3 ................................................................................
             T5392-1 ................................................................................

                           170       180       190       200       210       220       230       240
                    ....|....|....|....|....|....|....|....|....|....|....|....|....|....|....|....|
standard     H2114  ATAAGAGTCCCCTTCGTCGGATGCACGATTAGTGGTGGTTGTCAAGACCCTCTTGTCGAGTCGTGTGTTCAAAGGAGTAA
             H2126  ................................................................................
S. scandens  T5064-1 ................C...............................................................
             T5135-1 ................C...............................................................
             T5135-2 ................C...............................................................
             T5135-3 ................C...............................................................
             T5392-1 ................C...............................................................

                           250       260       270       280       290
                    ....|....|....|....|....|....|....|....|....|....|....|..
standard     H2114  GGAAGATCTCTTCGATGACCCTAAAGTGTCGTCTTGTACGATGCTCCGACAGCGACC
             H2126  .........................................................
S. scandens  T5064-1 ............T...........T................................
             T5135-1 ............T...........T................................
             T5135-2 ............T...........T................................
             T5135-3 ............T...........T................................
             T5392-1 ............T...........T................................
```

1c. Sequence alignment at the *ITS2* region (*Senecio scandens* Buch.-Ham. ex D.Don specific primers)

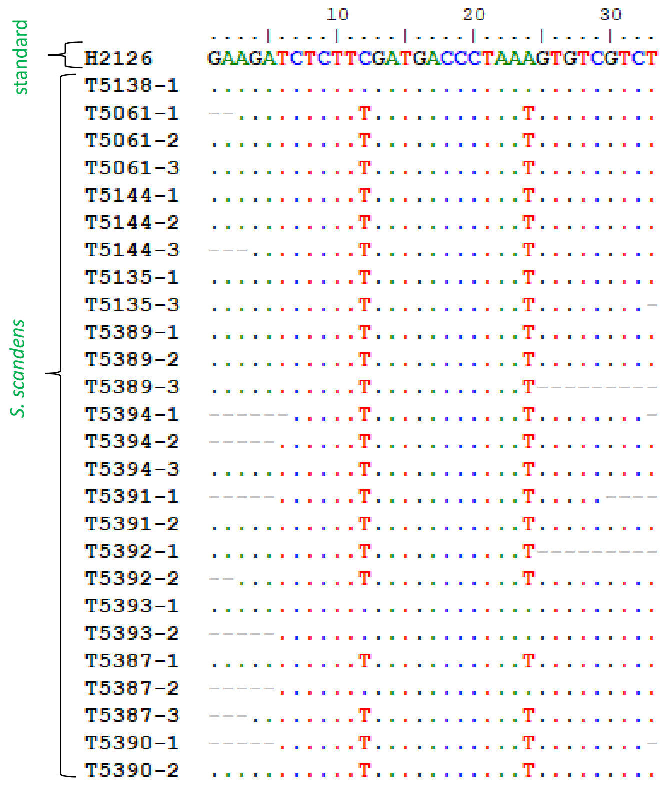

2. Sequence alignment at the *psbA-trnH* region

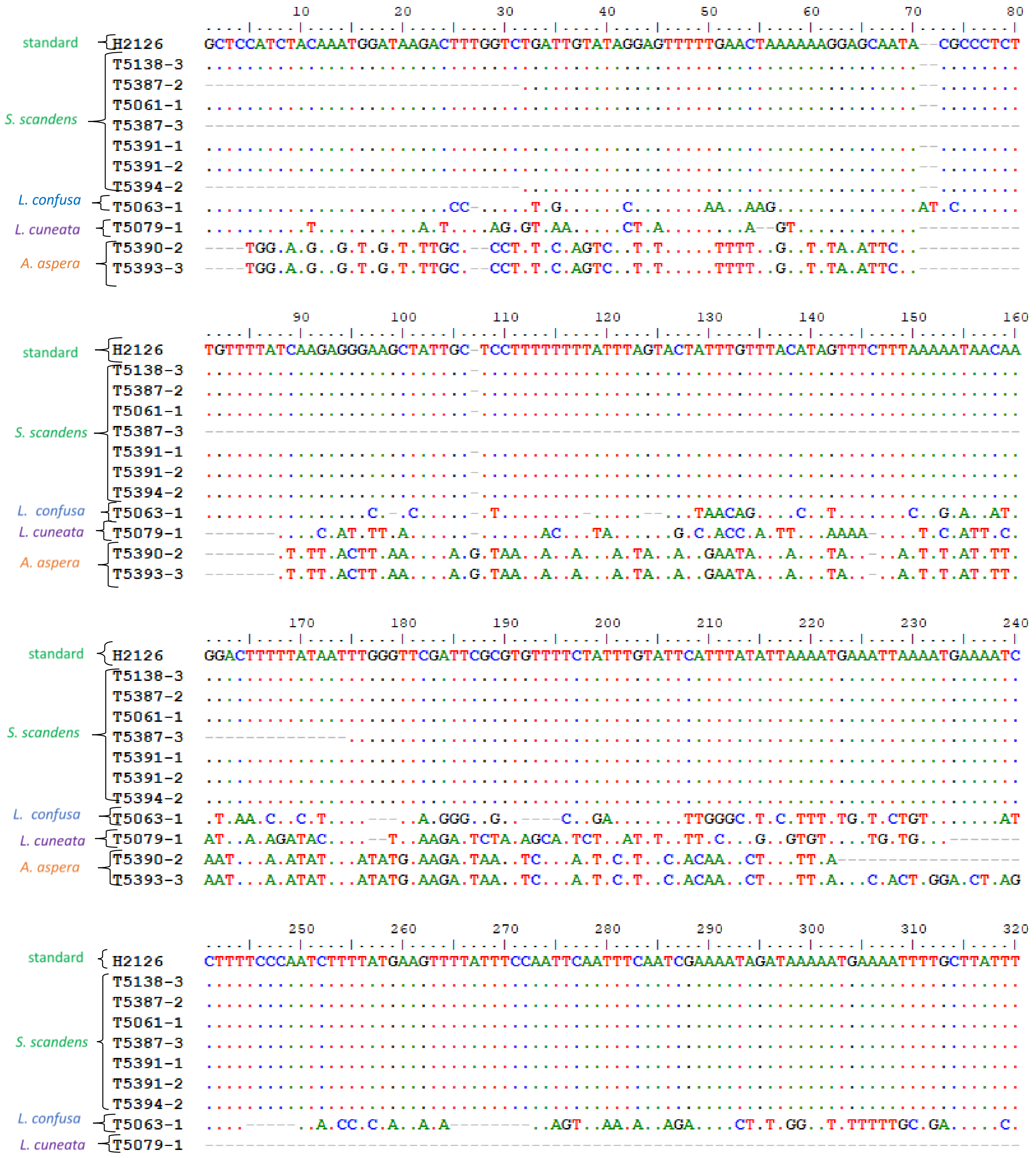

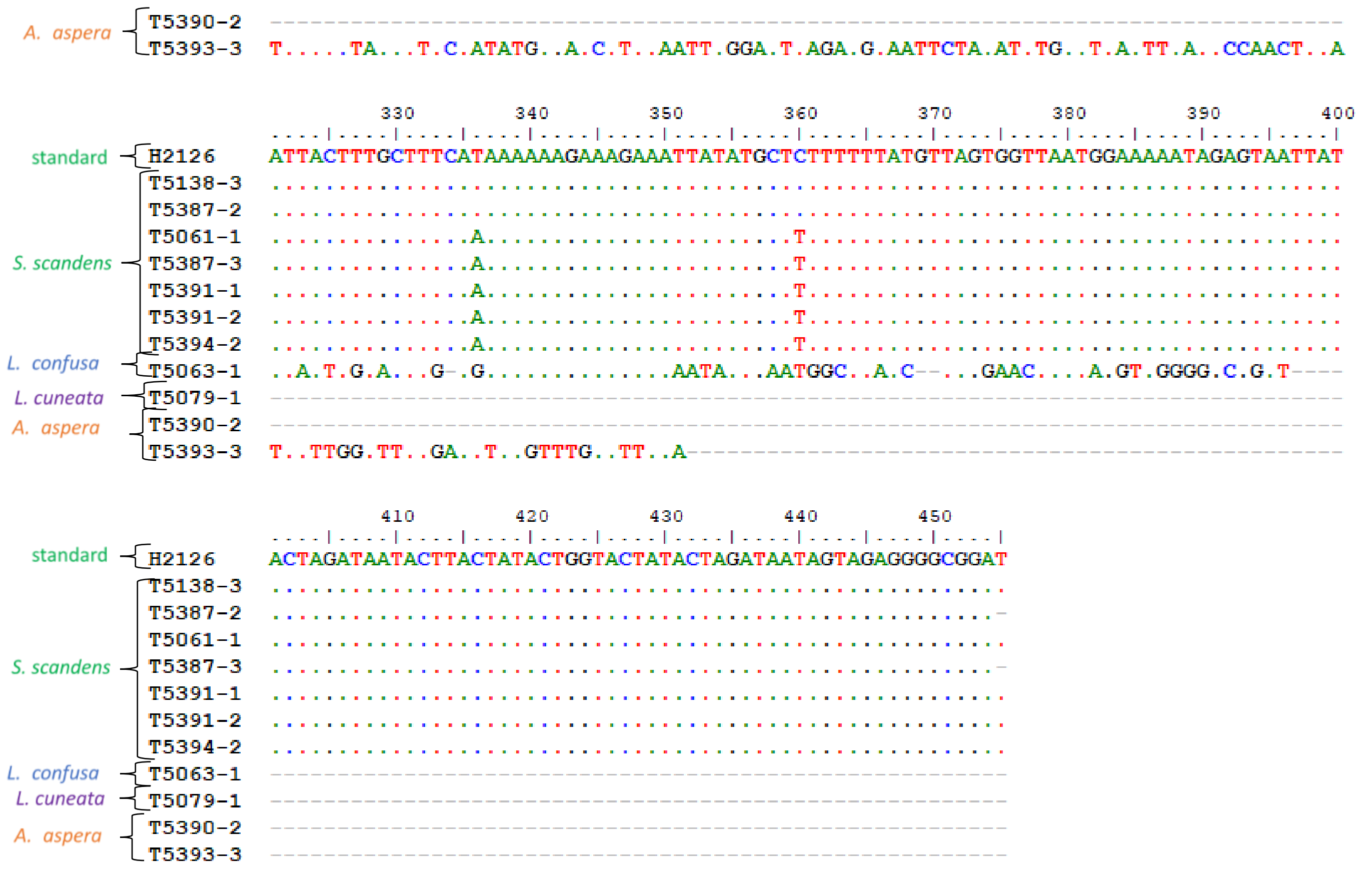

A. aspera
T5390-2
T5393-3
330
340
350
360
370
380
390
400
standard
H2126
T5138-3
T5387-2
T5061-1
S. scandens
T5387-3
T5391-1
T5391-2
T5394-2
L. confusa
T5063-1
L. cuneata
T5079-1
A. aspera
T5390-2
T5393-3
410
420
430
440
450
standard
H2126
T5138-3
T5387-2
T5061-1
S. scandens
T5387-3
T5391-1
T5391-2
T5394-2
L. confusa
T5063-1
L. cuneata
T5079-1
A. aspera
T5390-2
T5393-3

3. Sequence alignment at the *rbcL* region

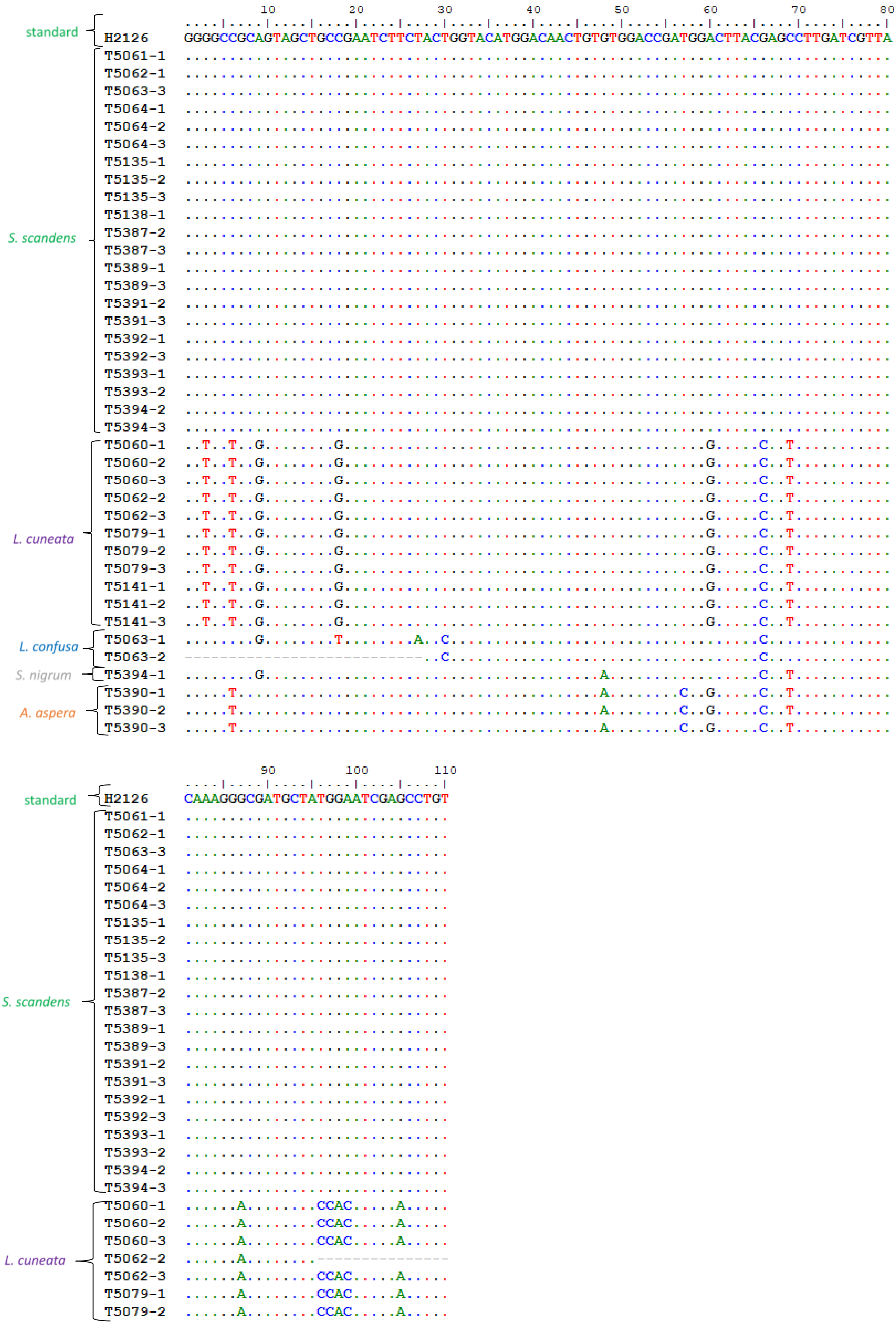

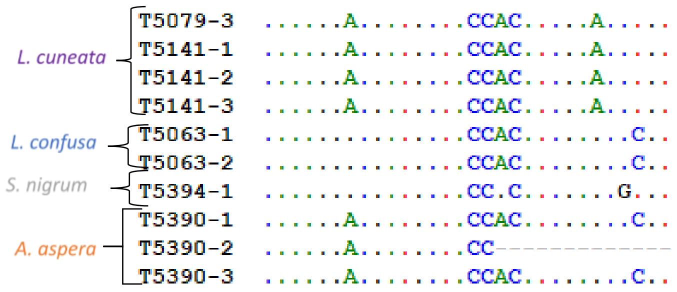
L. cuneata
L. confusa
S. nigrum
A. aspera
T5079-3 ......A........CCAC....A.....
T5141-1 ......A........CCAC....A.....
T5141-2 ......A........CCAC....A.....
T5141-3 ......A........CCAC....A.....
T5063-1 ...............CCAC.......C..
T5063-2 ...............CCAC.......C..
T5394-1 ...............CC.C......G...
T5390-1 ......A........CCAC.......C..
T5390-2 ......A........CC
T5390-3 ......A........CCAC.......C..
