# Supplementary material for: Multi-methodological approach for the Quality assessment of Senecionis scandentis Herba (Qianliguang) in the herbal market
Source: PLoS One. 2022 Apr 14;17(4):e0267143. doi: 10.1371/journal.pone.0267143 (PMC9009707; doi:10.1371/journal.pone.0267143)
Supplement: S5 File — (DOCX) [file pone.0267143.s005.docx]

**S5 File. DNA concentration and purity of samples**

| **Sample** | **Extraction no.** | **DNA concentration (μg/μl)** | **DNA purity (A260/A280)** |
| --- | --- | --- | --- |
| T5060 | T5060-1 | 18.1 | 1.95 |
|  | T5060-2 | 7.7 | 1.94 |
|  | T5060-3 | 24.9 | 1.78 |
| T5061 | T5061-1 | 27.2 | 1.96 |
|  | T5061-2 | 10.9 | 1.54 |
|  | T5061-3 | 14.5 | 1.74 |
| T5062 | T5062-1 | 18.6 | 1.67 |
|  | T5062-2 | 27.8 | 1.81 |
|  | T5062-3 | 17.5 | 1.78 |
| T5063 | T5063-1 | 5.9 | 1.41 |
|  | T5063-2 | 4.2 | 1.37 |
|  | T5063-3* | 26.2 | 1.32 |
| T5064 | T5064-1 | 7.1 | 1.49 |
|  | T5064-2 | 9.7 | 1.54 |
|  | T5064-3 | 74.7 | 1.77 |
| T5079 | T5079-1 | 4.8 | 1.8 |
|  | T5079-2 | 8.4 | 1.64 |
|  | T5079-3 | 9.7 | 1.8 |
| T5135 | T5135-1* | 2.6 | 1.41 |
|  | T5135-2* | 2.2 | 1.41 |
|  | T5135-3* | 1.6 | 1.6 |
| T5138 | T5138-1 | 15.9 | 1.72 |
|  | T5138-2 | 20.3 | 1.84 |
|  | T5138-3 | 20.5 | 1.89 |
| T5141 | T5141-1 | 7.2 | 1.9 |
|  | T5141-2 | 51.1 | 2.01 |
|  | T5141-3 | 20.4 | 1.95 |
| T5144 | T5144-1 | 9.2 | 1.65 |
|  | T5144-2 | 8.2 | 1.62 |
|  | T5144-3 | 12.1 | 1.87 |
| T5387 | T5387-1 | 3.8 | 1.7 |
|  | T5387-2 | 14.6 | 1.72 |
|  | T5387-3 | 26.4 | 1.79 |
| T5388 | T5388-1* | 148.2 | 1.91 |
|  | T5388-2* | 143.4 | 1.87 |
|  | T5388-3* | 147.1 | 1.65 |
| T5389 | T5389-1 | 5.4 | 1.77 |
|  | T5389-2 | 32.4 | 1.66 |
|  | T5389-3 | 14.9 | 1.91 |
| T5390 | T5390-1 | 6.9 | 1.57 |
|  | T5390-2 | 38.6 | 1.68 |
|  | T5390-3 | 14.3 | 1.56 |
| T5391 | T5391-1 | 10.6 | 1.58 |
|  | T5391-2 | 19.5 | 1.8 |
|  | T5391-3 | 13.8 | 1.49 |
| T5392 | T5392-1 | 5.4 | 2.05 |
|  | T5392-2 | 13.4 | 1.32 |
|  | T5392-3 | 4.7 | 1.71 |
| T5393 | T5393-1 | 8.5 | 1.38 |
|  | T5393-2 | 15.8 | 1.71 |
|  | T5393-3 | 18.3 | 1.55 |
| T5394 | T5394-1 | 8 | 1.88 |
|  | T5394-2 | 9.1 | 1.72 |
|  | T5394-3 | 11.3 | 1.77 |
| H2114^ | / | 15 | 2.1 |
| H2126^ | /* | 1.3 | 1.83 |

Note: “*” indicates samples have been diluted ten-fold to improve PCR amplification success.

“^” indicates fresh leaves of Qianliguang for setting DNA standard.
