# Supplementary material for: Multi-methodological approach for the Quality assessment of Senecionis scandentis Herba (Qianliguang) in the herbal market
Source: PLoS One. 2022 Apr 14;17(4):e0267143. doi: 10.1371/journal.pone.0267143 (PMC9009707; doi:10.1371/journal.pone.0267143)
Supplement: S6 File — (PDF) [file pone.0267143.s006.pdf]

S6 File. Chromatographs of HPLC-UV for the quantification of hyperoside

1. Raw data of the calibration curve of hyperoside

Injection quantity (ug)	0.4	0.8	1.2	1.6
Peak area (1st injection)	784.40894	1619.30872	2456.54175	3262.14502
Peak area (2nd injection)	781.59448	1573.98474	2349.9812	3312.80225
Peak area (3rd injection)	803.58582	1569.67358	2399.13232	3363.62744
**Mean**	789.86308	1587.65568	2401.88509	3312.858237
**Standard deviation**	11.96726749	27.49695858	53.33358239	50.74123317

Note: Injection quantity (ug) = injection volume (40 µL) * stock concentrations of 10 µg mL^{-1}, 20 µg mL^{-1}, 30 µg mL^{-1}, and 40 µg mL^{-1}.

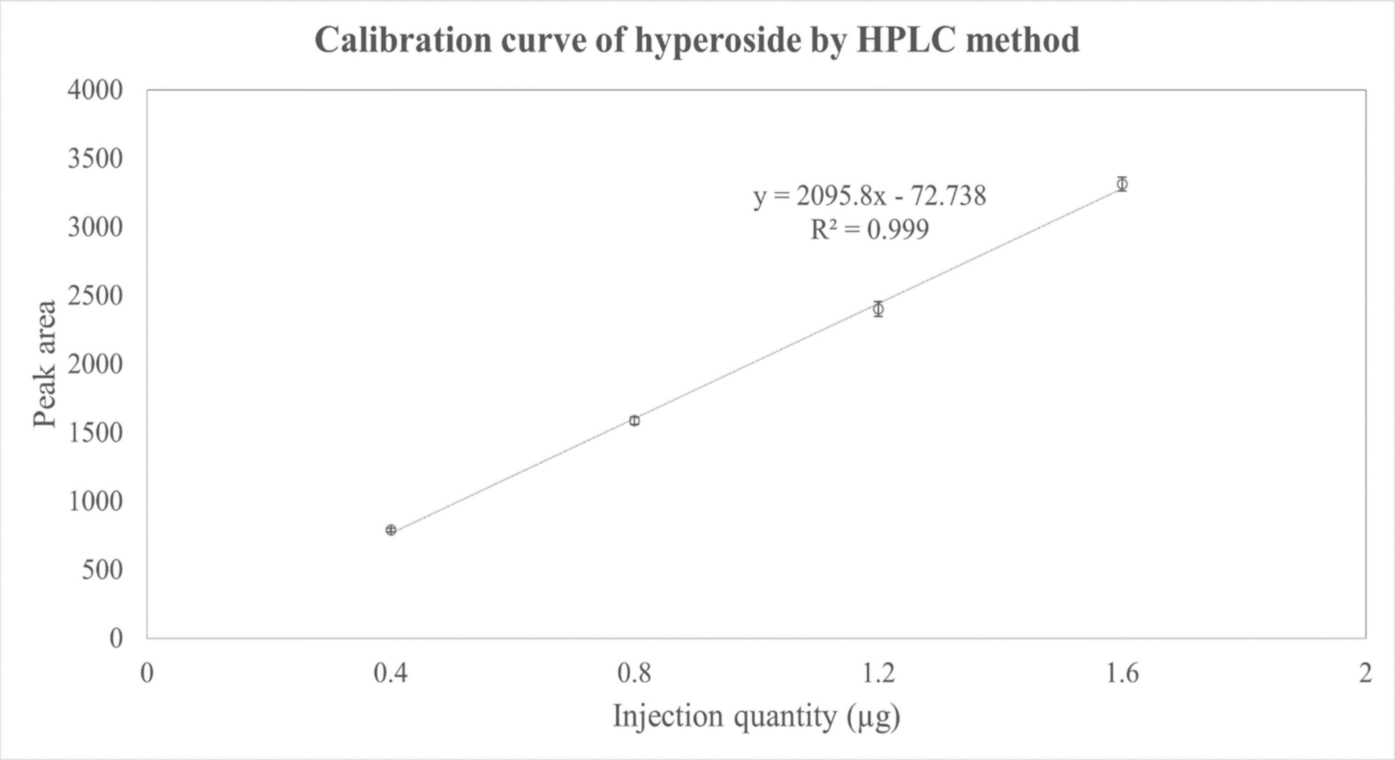

S32 Fig. Standard curve of hyperoside. Signal was detected at 360 nm. Injection quantity (ug) = injection volume (40 µL) * stock concentration of 10 µg mL^{-1}, 20 µg mL^{-1}, 30 µg mL^{-1}, and 40 µg mL^{-1}.

2. Identification of Hyperoside peak

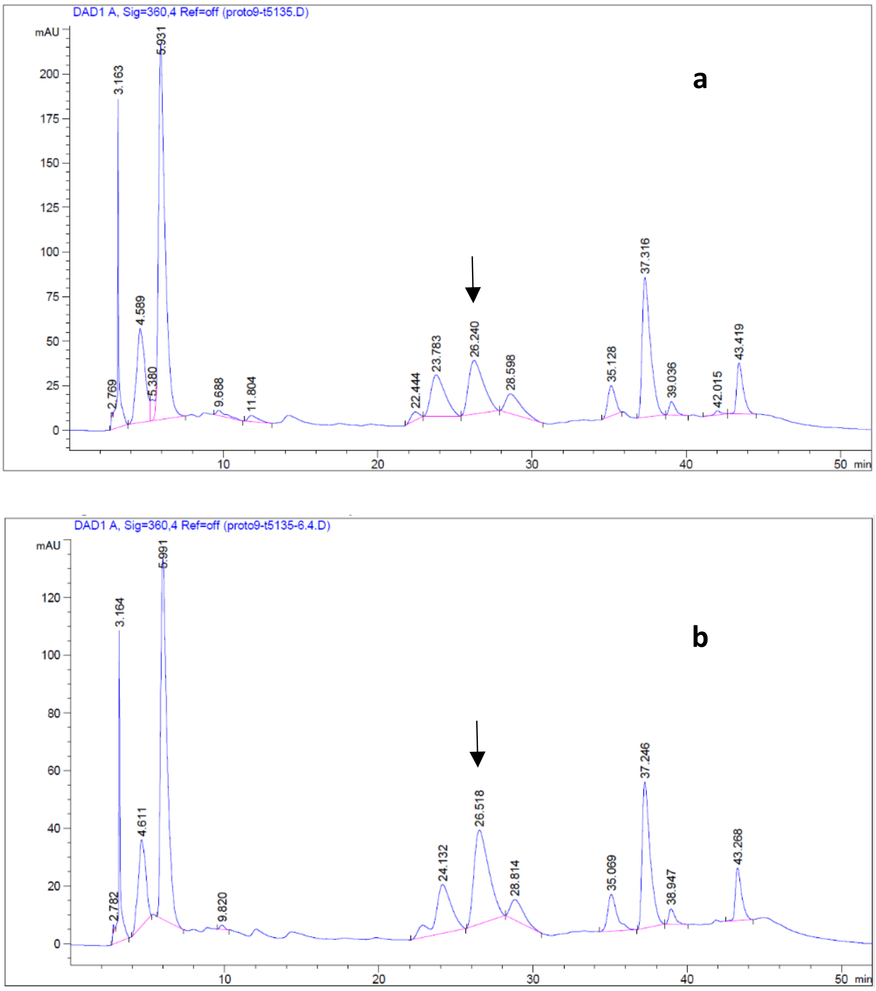

S33 Fig. Comparison of the elution profiles of T5135 with the mixture of T5135 and hyperoside. (a) Original sample elution profile of T5135, (b) S33b Fig. Elution profile of mixture of hyperoside and T5135 at a ratio of 4 to 6.

3. Elution profiles of three standard samples of ChP 2020 standard

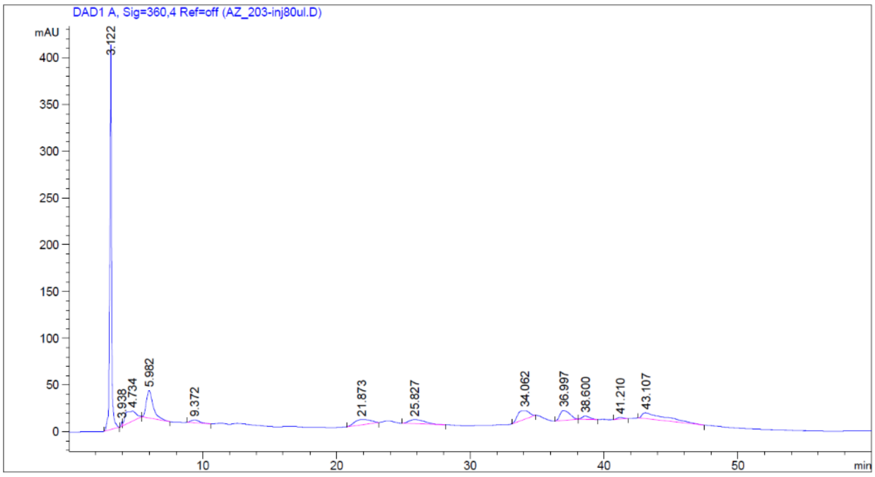

S34 Fig. Elution profile of AZ22011202.

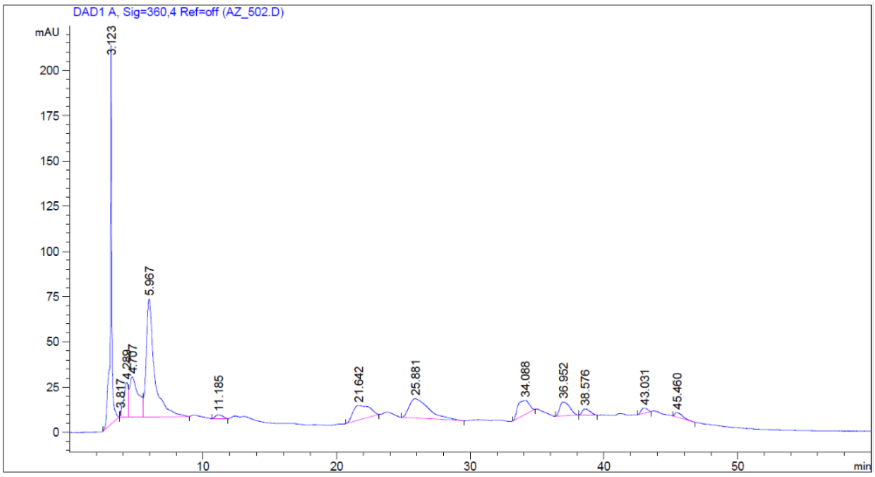

S35 Fig. Elution profile of AZ21110503.

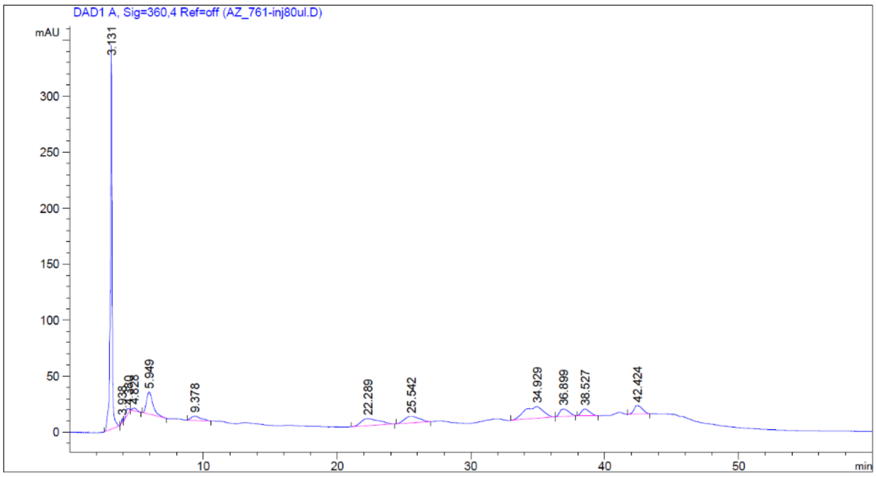

S36 Fig. Elution profile of AZ21110761.

4. Sample elution profiles

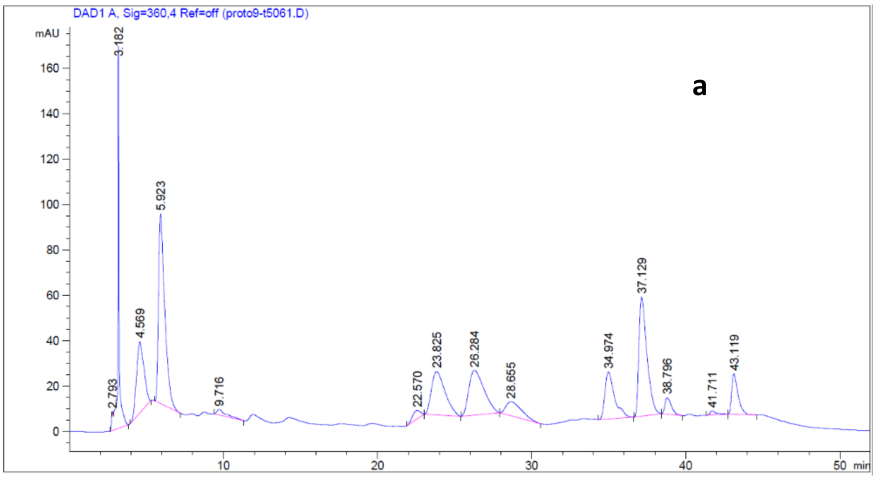

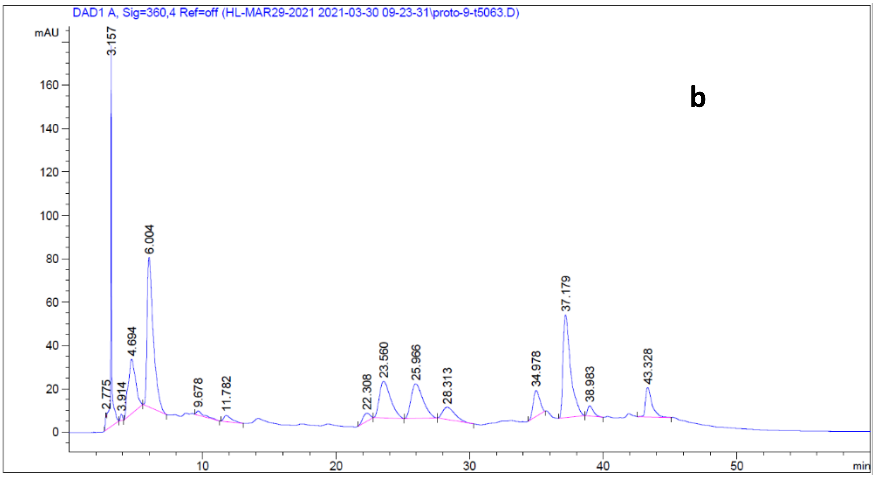

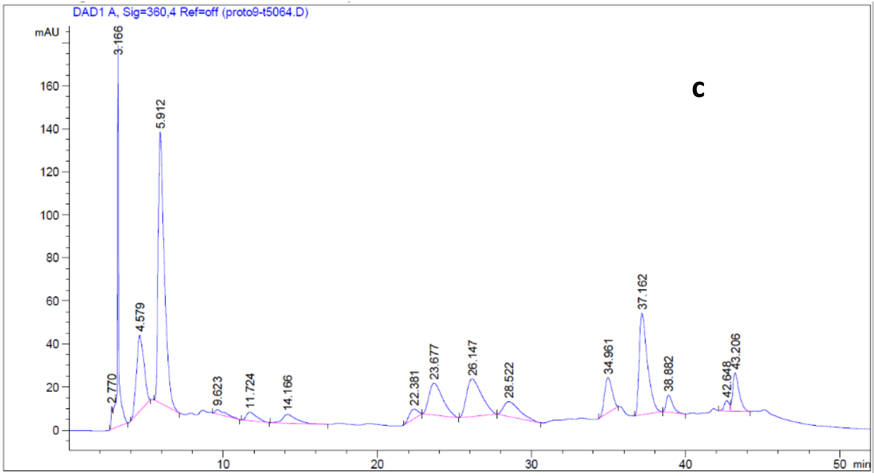

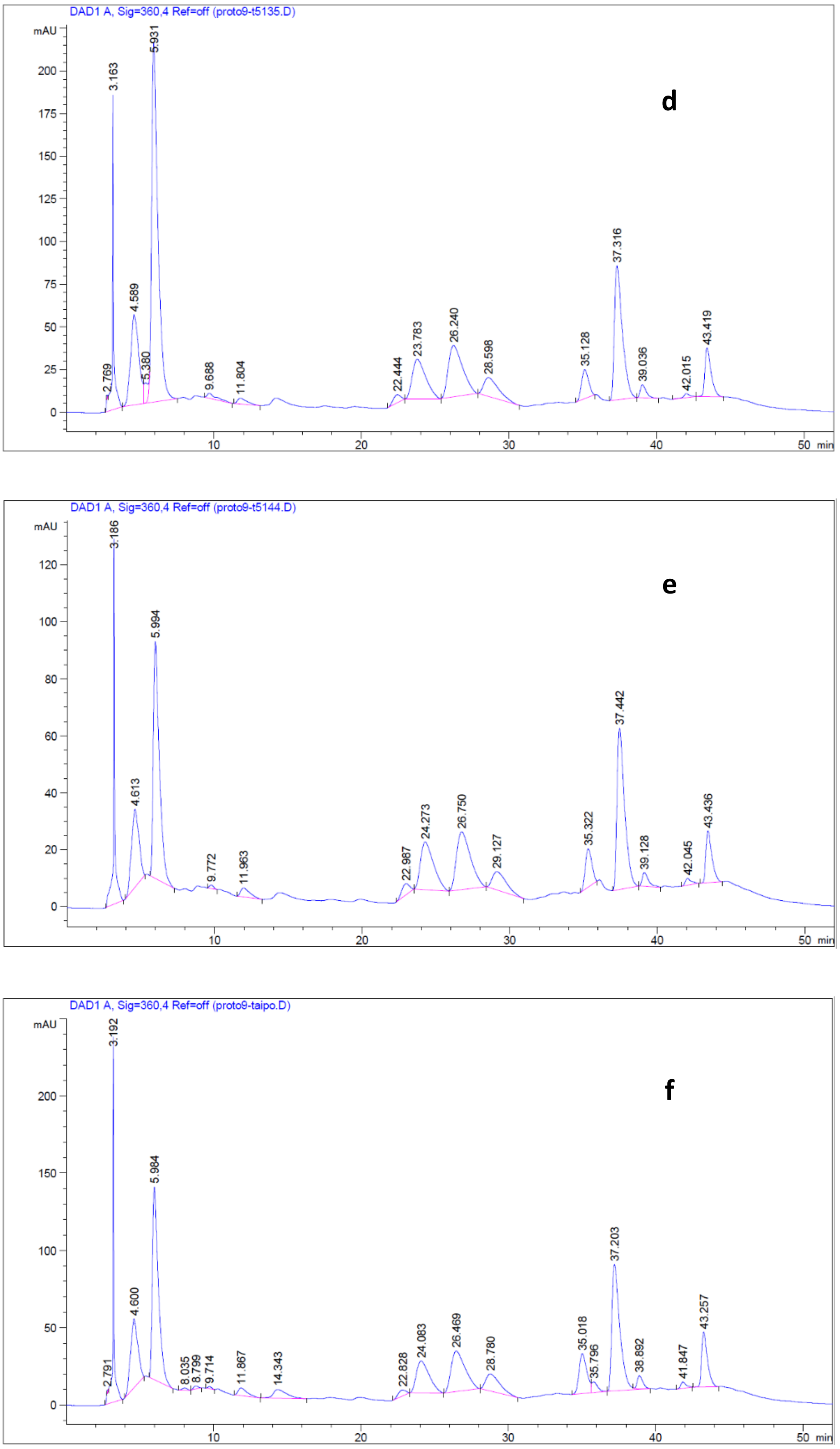
DAD1 A, Sig=360,4 Ref=off (proto9-t5135.D)
d
mAU
3.163
5.931
4.589
5.380
2.769
9.688
11.804
22.444
23.783
26.240
28.598
35.128
37.316
39.036
42.015
43.419
min
DAD1 A, Sig=360,4 Ref=off (proto9-t5144.D)
e
3.186
5.994
4.613
9.772
11.963
22.987
24.273
26.750
29.127
35.322
37.442
39.128
42.045
43.436
DAD1 A, Sig=360,4 Ref=off (proto9-taipo.D)
f
3.192
5.984
4.600
2.791
8.035
8.799
9.714
11.867
14.343
22.828
24.083
26.469
28.780
35.018
35.796
37.203
38.892
41.847
43.257

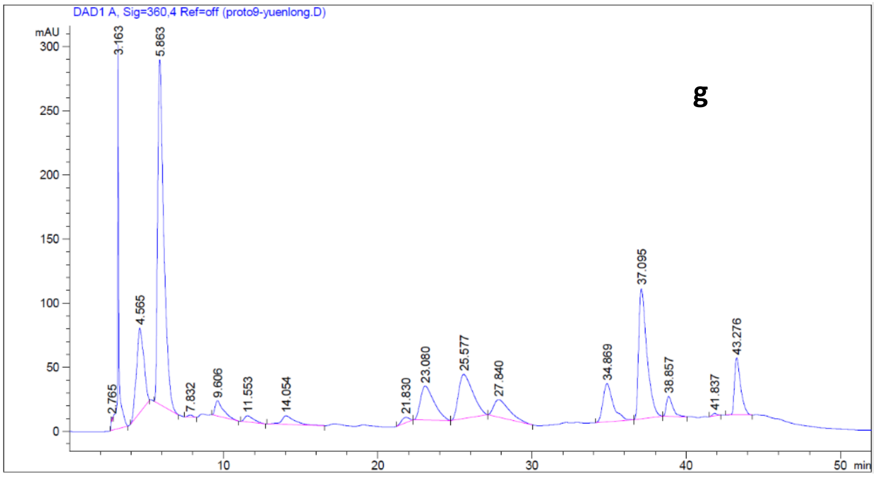
DAD1 A, Sig=360,4 Ref=off (proto9-yuenlong.D)
mAU
g
3.163
5.863
4.565
2.765
7.832
9.606
11.553
14.054
21.830
23.080
25.577
27.840
34.869
37.095
38.857
41.837
43.276
min

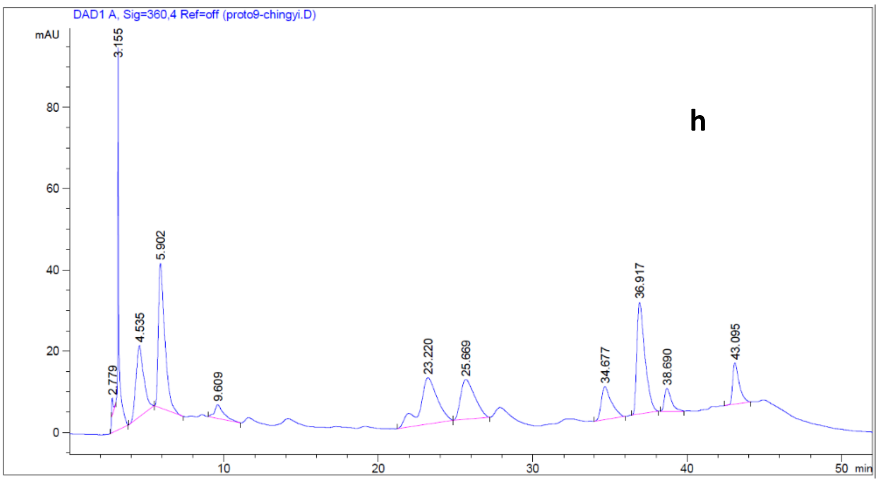
DAD1 A, Sig=360,4 Ref=off (proto9-chingyi.D)
mAU
h
3.155
5.902
4.535
2.779
9.609
23.220
25.669
34.677
36.917
38.690
43.095
min

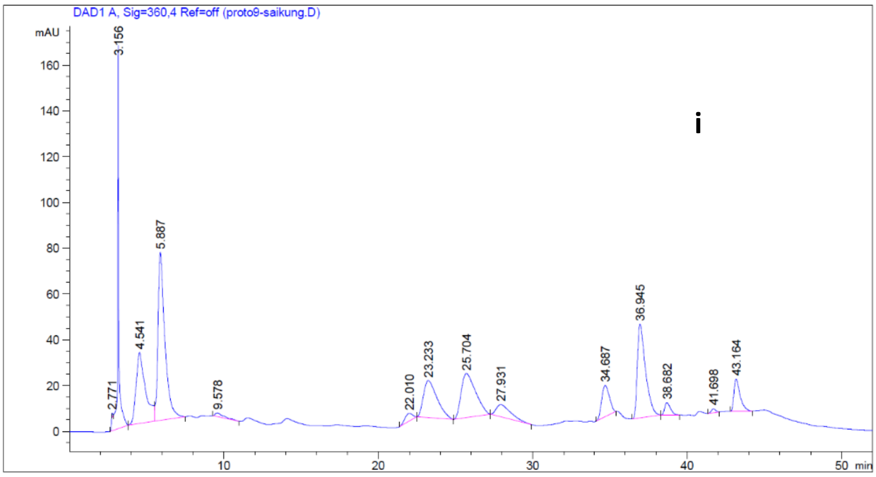
DAD1 A, Sig=360,4 Ref=off (proto9-saikung.D)
mAU
i
3.156
5.887
4.541
2.771
9.578
22.010
23.233
25.704
27.931
34.687
36.945
38.682
41.698
43.164
min

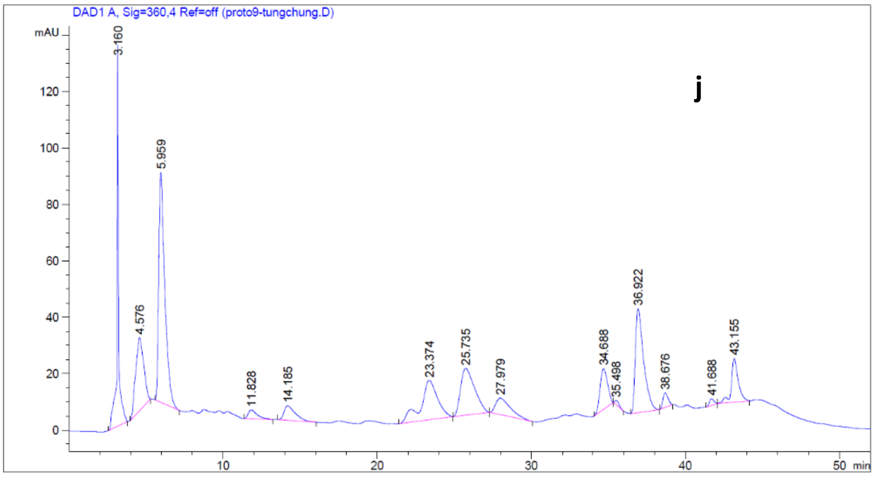

S37 Fig. Elution profiles of samples Senecio scandens Buch.-Ham. ex D.Don (≤2% adulterations) detected at UV 360 nm. (a) T5061 elution profile, (b) T5063 elution profile, (c) T5064 elution profile, (d) T5135 elution profile, (e) T5144 elution profile, (f) T5387 elution profile, (g) T5389 elution profile, (h) T5391 elution profile, (i) T5392 Elution profile, (j) T5394 Elution profile.

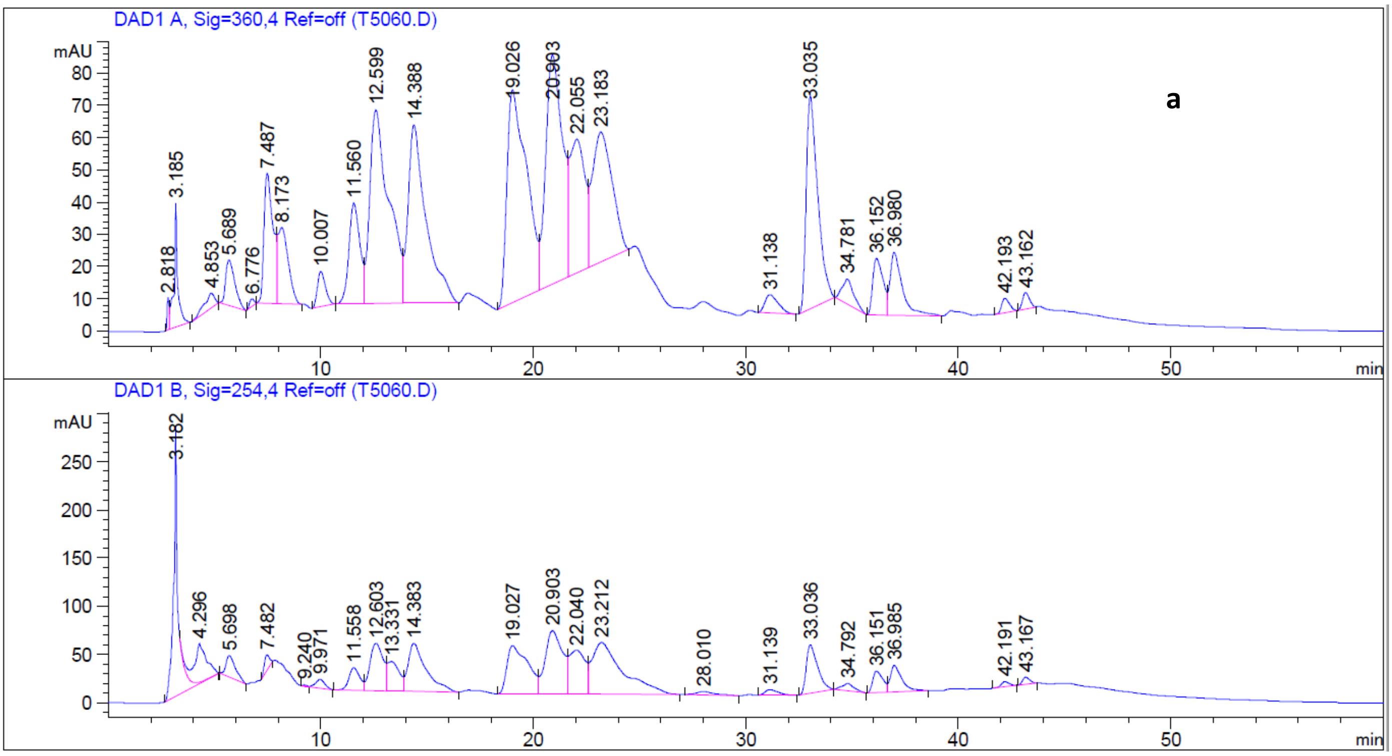
DAD1 A, Sig=360,4 Ref=off (T5060.D)
a
mAU
min
2.818
3.185
4.853
5.689
6.776
7.487
8.173
10.007
11.560
12.599
14.388
19.026
20.903
22.055
23.183
31.138
33.035
34.781
36.152
36.980
42.193
43.162
DAD1 B, Sig=254,4 Ref=off (T5060.D)
3.182
4.296
5.698
7.482
9.240
9.971
11.558
12.603
13.331
14.383
19.027
20.903
22.040
23.212
28.010
31.139
33.036
34.792
36.151
36.985
42.191
43.167

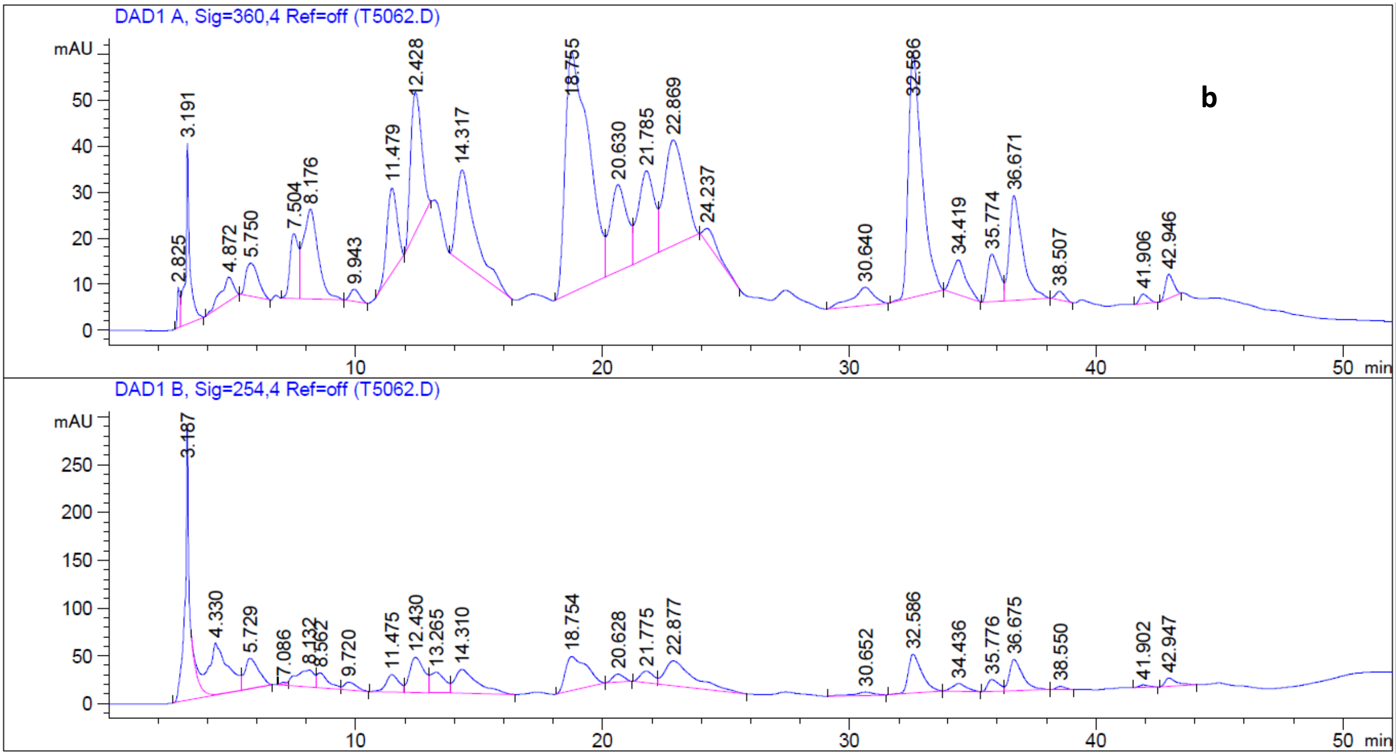
DAD1 A, Sig=360,4 Ref=off (T5062.D)
b
mAU
min
2.825
3.191
4.872
5.750
7.504
8.176
9.943
11.479
12.428
14.317
18.755
20.630
21.785
22.869
24.237
30.640
32.586
34.419
35.774
36.671
38.507
41.906
42.946
DAD1 B, Sig=254,4 Ref=off (T5062.D)
3.187
4.330
5.729
7.086
8.132
8.562
9.720
11.475
12.430
13.265
14.310
18.754
20.628
21.775
22.877
30.652
32.586
34.436
35.776
36.675
38.550
41.902
42.947

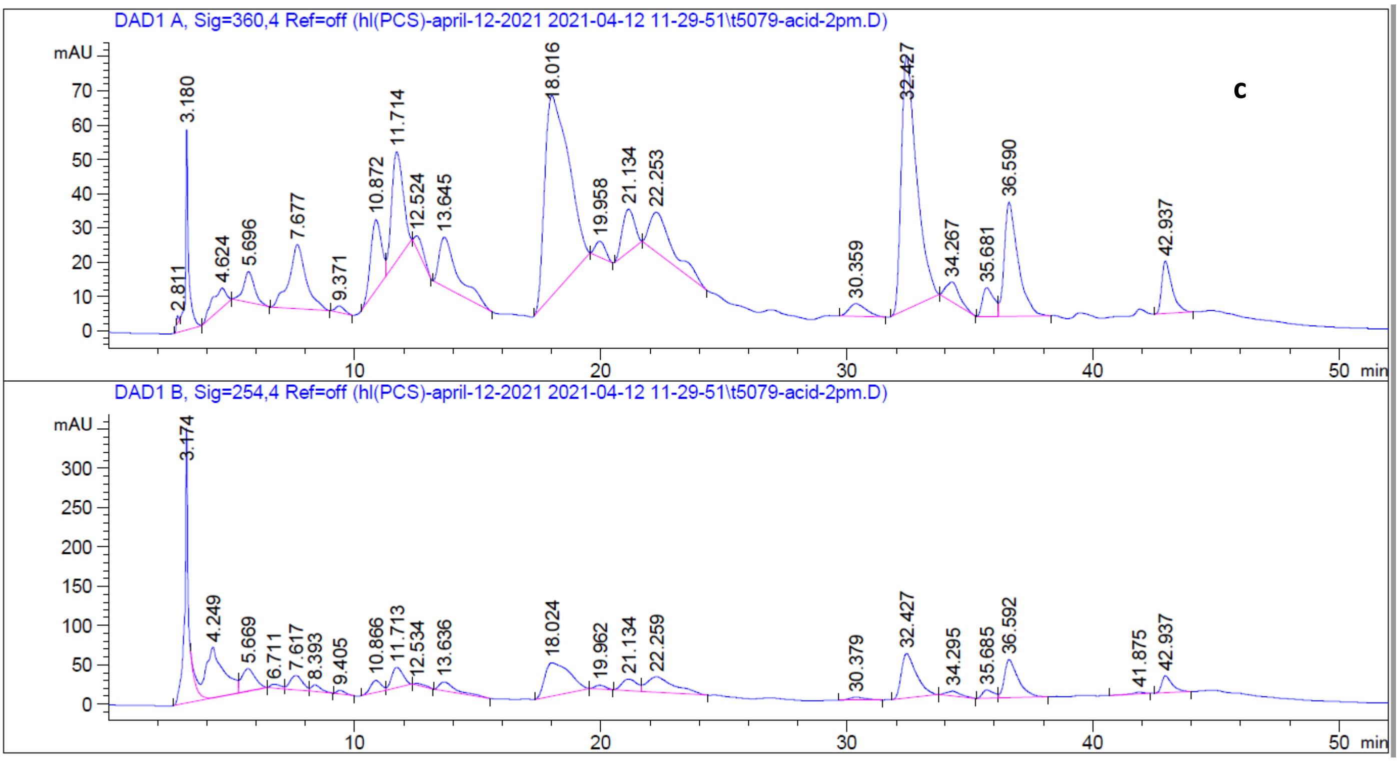
DAD1 A, Sig=360,4 Ref=off (hl(PCS)-april-12-2021 2021-04-12 11-29-51\t5079-acid-2pm.D)
c
DAD1 B, Sig=254,4 Ref=off (hl(PCS)-april-12-2021 2021-04-12 11-29-51\t5079-acid-2pm.D)
mAU
min

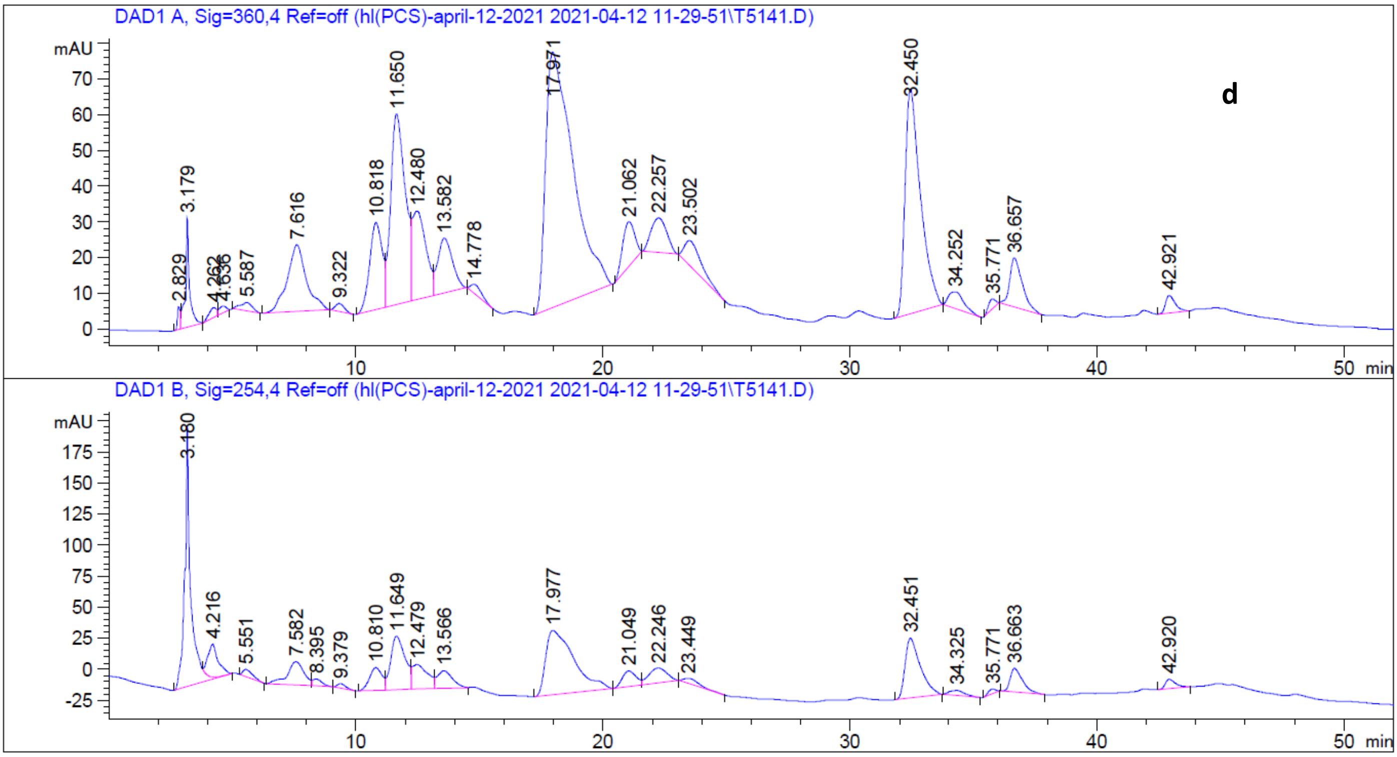
DAD1 A, Sig=360,4 Ref=off (hl(PCS)-april-12-2021 2021-04-12 11-29-51\T5141.D)
d
DAD1 B, Sig=254,4 Ref=off (hl(PCS)-april-12-2021 2021-04-12 11-29-51\T5141.D)
mAU
min

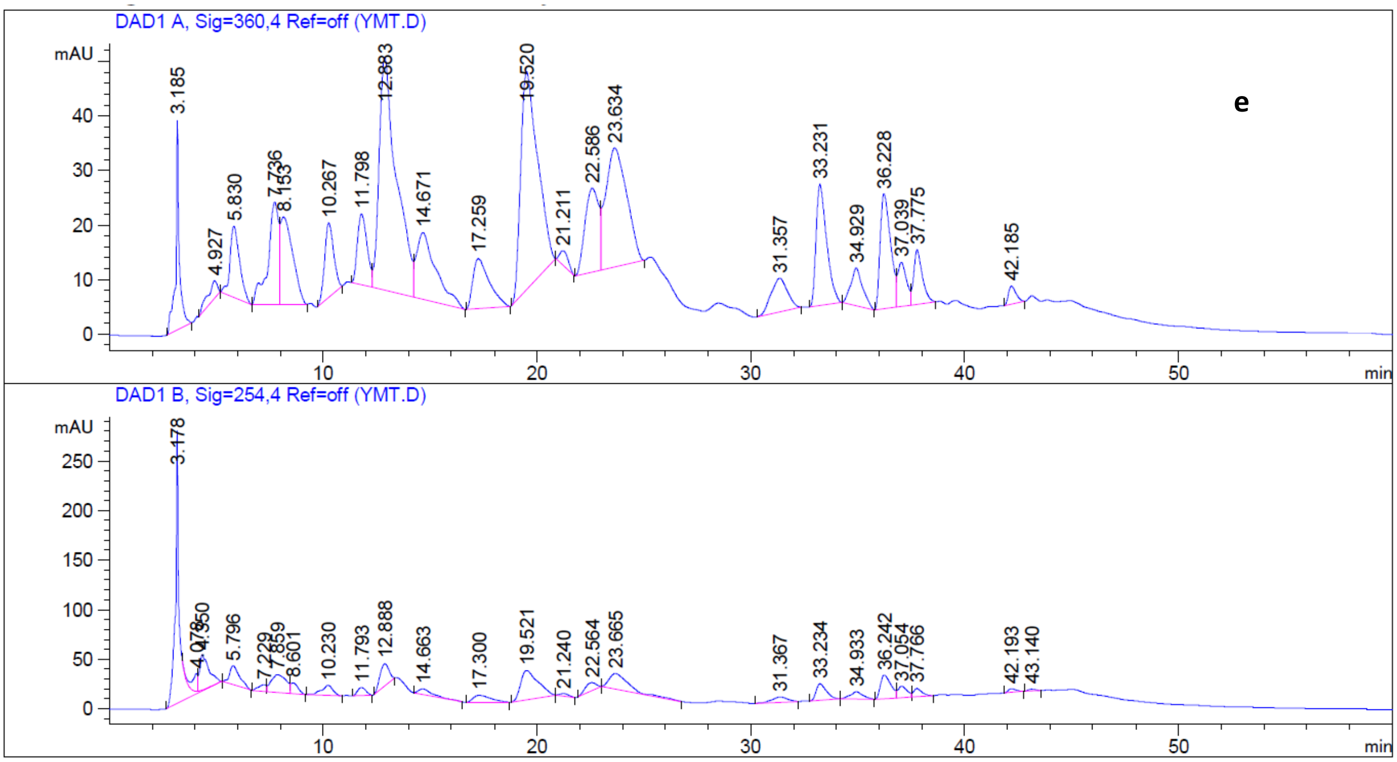

S38 Fig. Elution profiles of samples *Lespedeza*, at UV 360 nm and 254 nm. (a) T5060 elution profile, (b) T5062 elution profile, (c) T5079 elution profile, (d) T5141 elution profile, (e) T5388 elution profile.

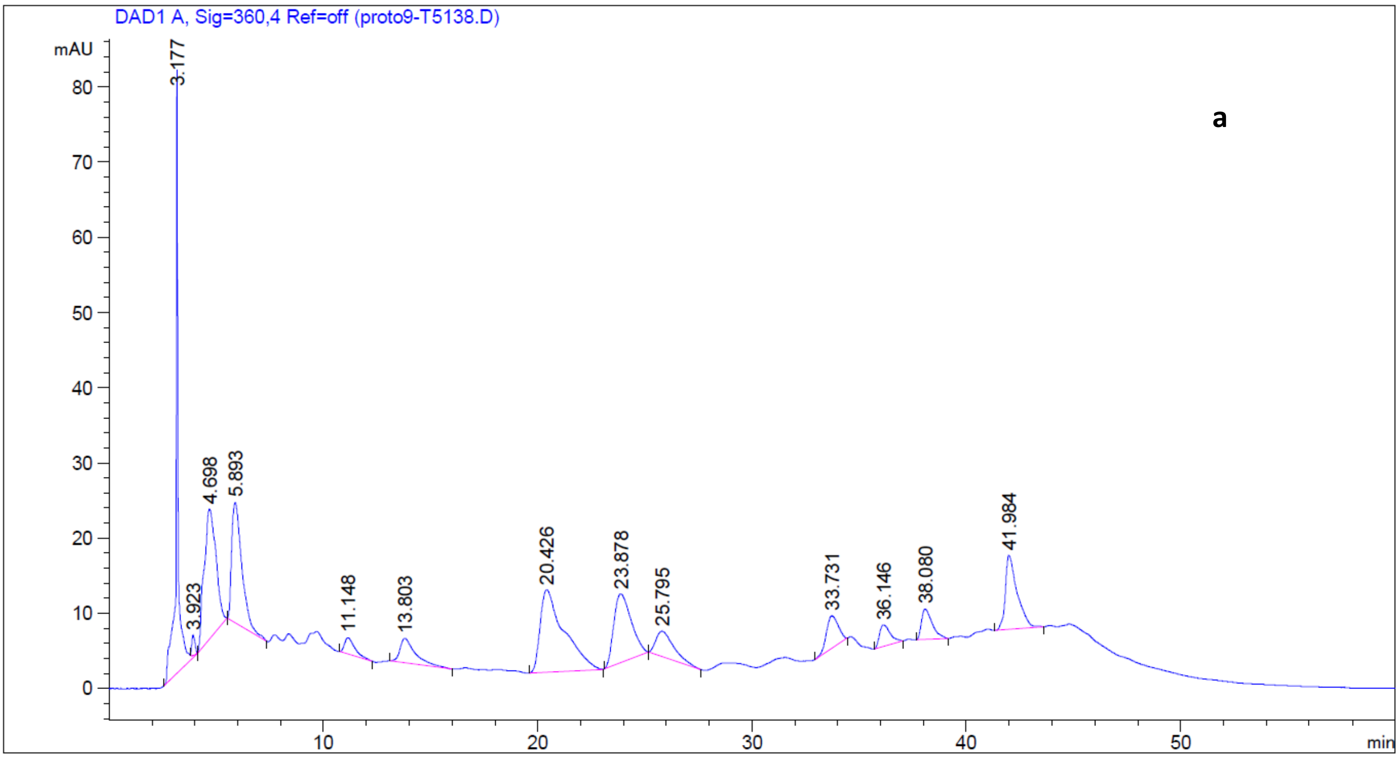
DAD1 A, Sig=360,4 Ref=off (proto9-T5138.D)
mAU
a
3.177
3.923
4.698
5.893
11.148
13.803
20.426
23.878
25.795
33.731
36.146
38.080
41.984
min

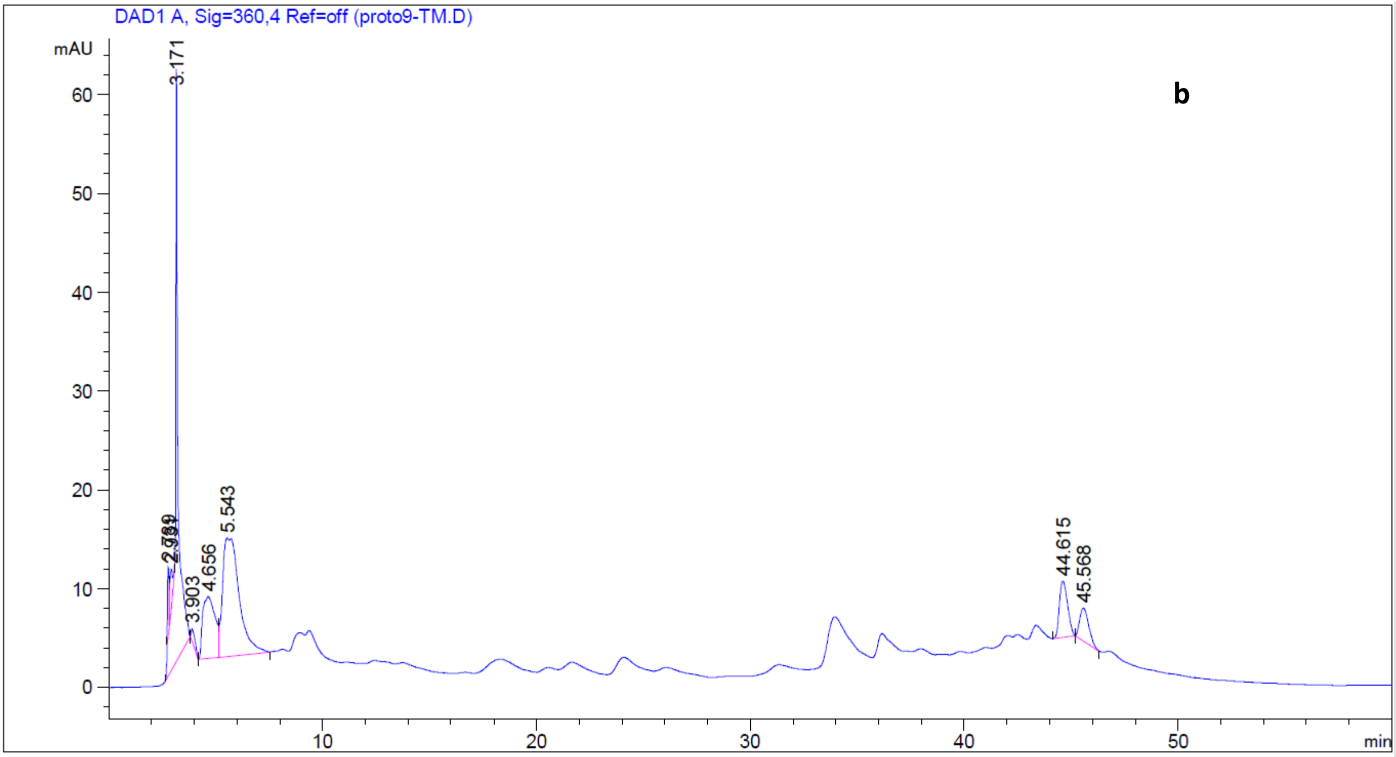
DAD1 A, Sig=360,4 Ref=off (proto9-TM.D)
mAU
b
3.171
3.903
4.656
5.543
44.615
45.568
min

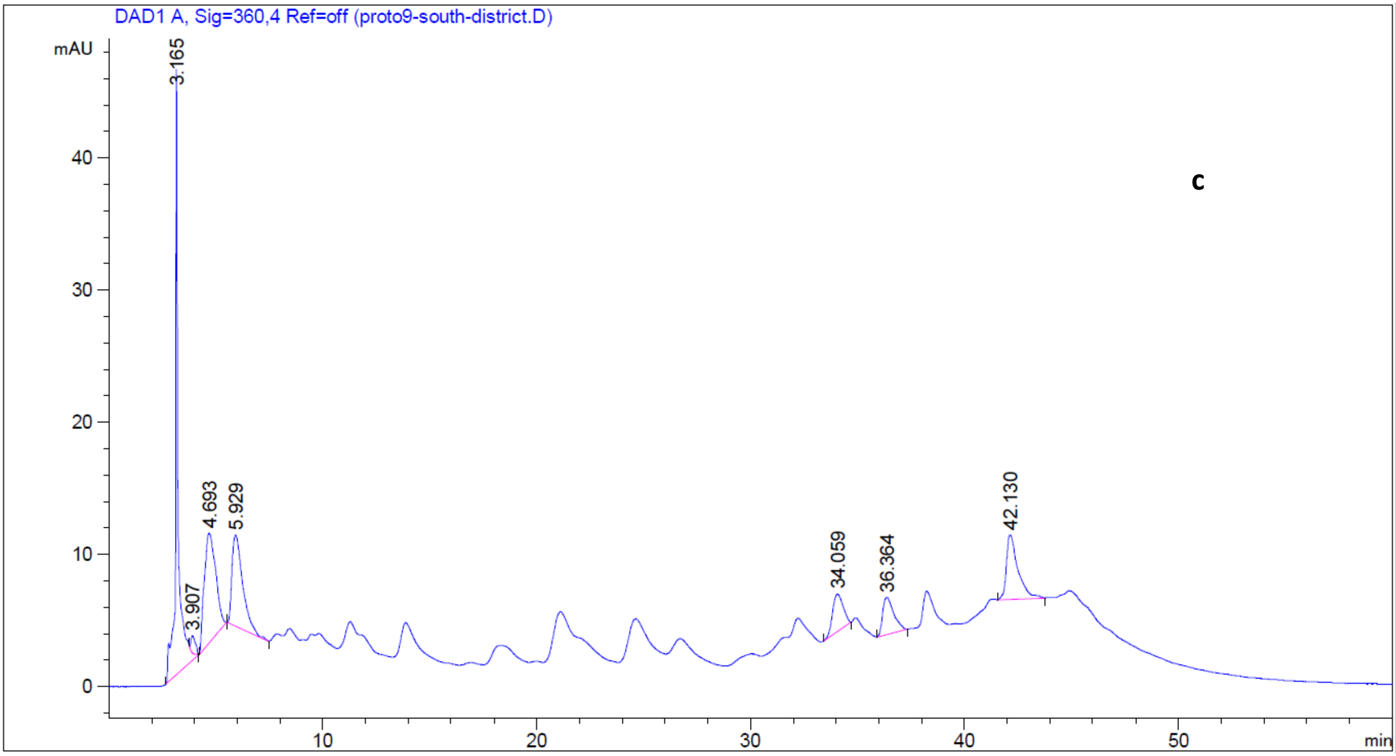

S39 Fig. Elution profiles of samples adulterated with Achyranthes aspera Linnaeus, at UV 360 nm. (a) T5138 elution profile, (b) T5390 elution profile, (c) T5393 elution profile.
