# Supplementary material for: Multi-methodological approach for the Quality assessment of Senecionis scandentis Herba (Qianliguang) in the herbal market
Source: PLoS One. 2022 Apr 14;17(4):e0267143. doi: 10.1371/journal.pone.0267143 (PMC9009707; doi:10.1371/journal.pone.0267143)
Supplement: S7 File — (PDF) [file pone.0267143.s007.pdf]

S7 File. Results of UPLC-MS

1. The UPLC-MS results of chemical standard mixture

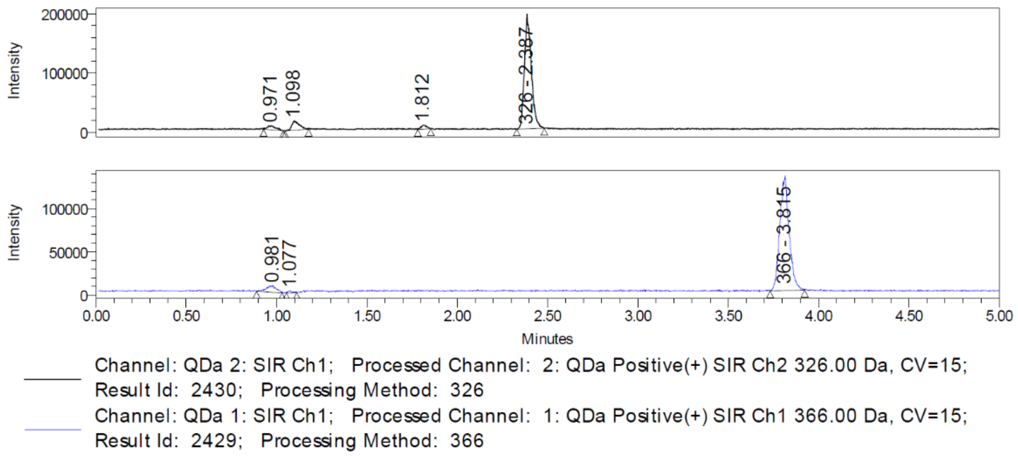

S40 Fig. UPLC-MS of chemical standard mixture adonifoline and crotaline

2. The percentages of adonifoline in three standard samples

Standard sample	Percentage
AZ22011202	0.000462%
AZ21110503	0.000119%
AZ22021761	0

Note: Adonifoline content in the three standard samples met the requirement as stated in Chinese Pharmacopoeia 2020.

3. The elution profiles of three standard samples

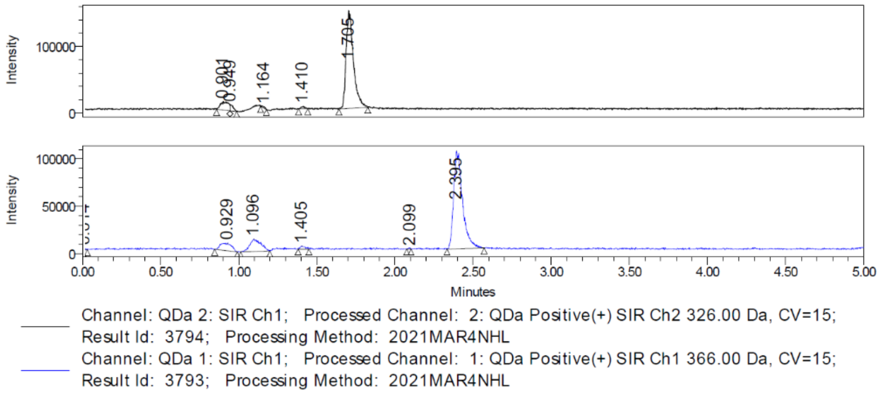

S41 Fig. UPLC-MS of standard sample AZ22011202

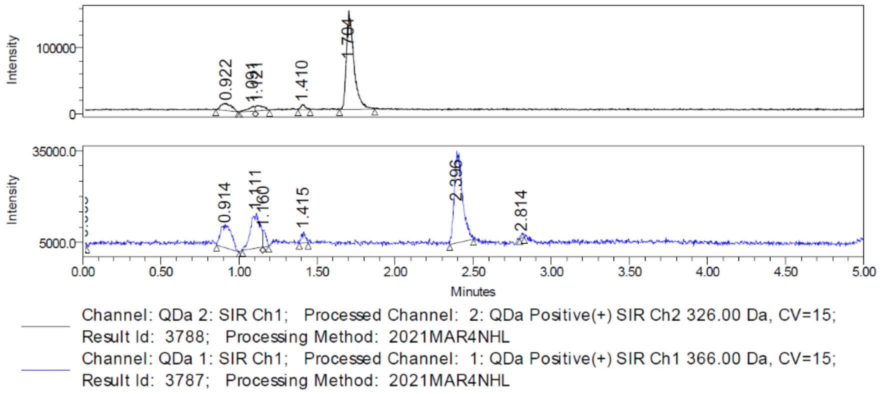

S42 Fig. UPLC-MS of standard sample AZ21110503

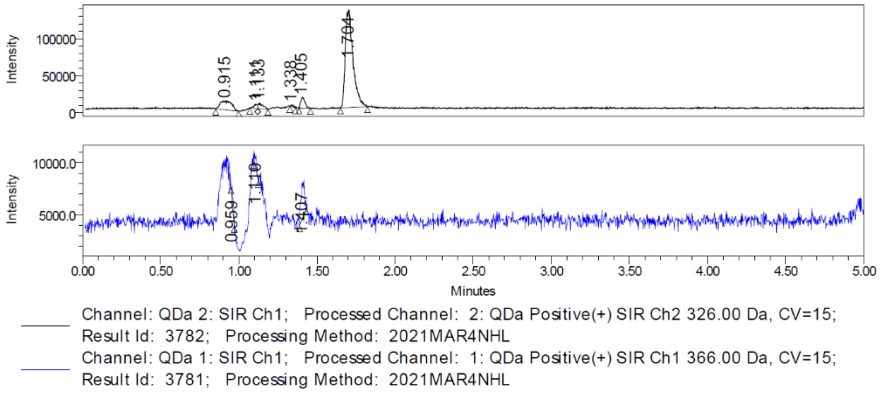

S43 Fig. UPLC-MS of standard sample AZ22021761

4. The UPLC-MS results of samples containing mainly *S. scandens*

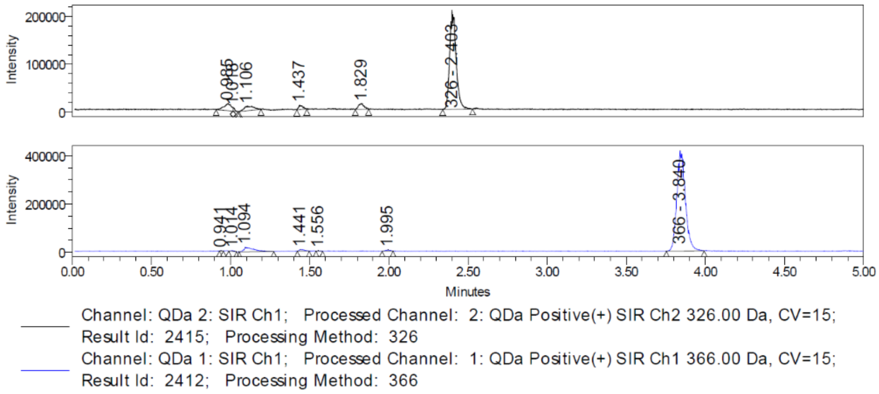

S44 Fig. UPLC-MS of T5061

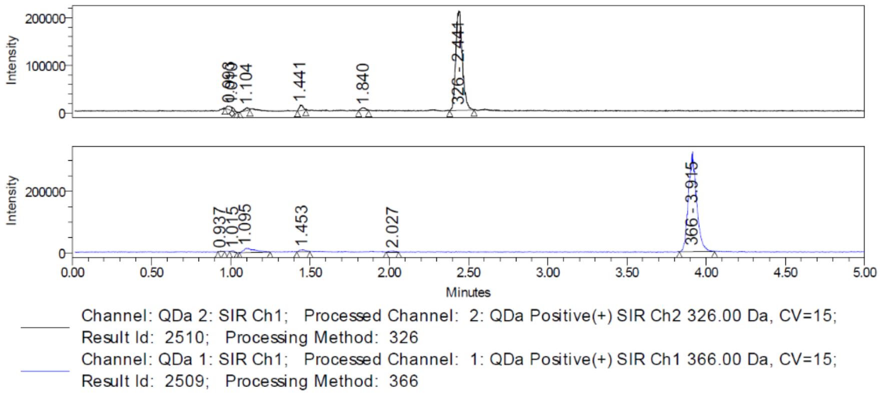

S45 Fig. UPLC-MS of T5063

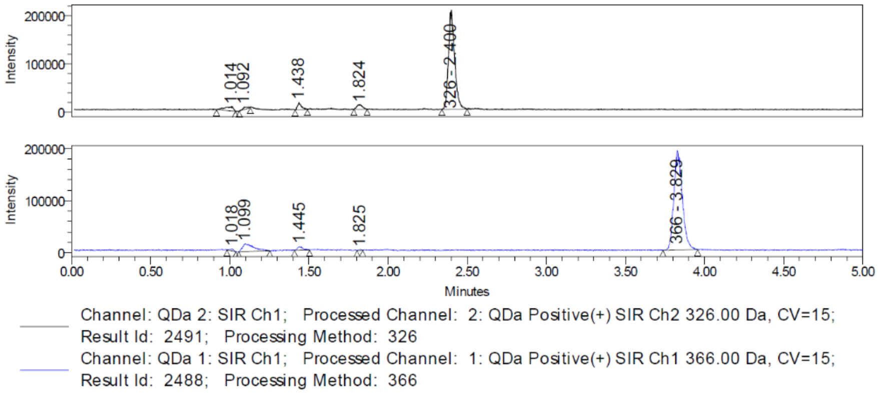

S46 Fig. UPLC-MS of T5064

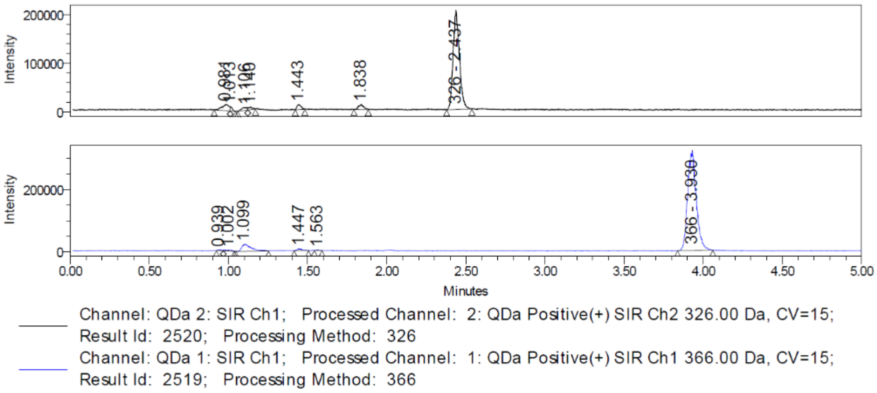

S47 Fig. UPLC-MS of T5135

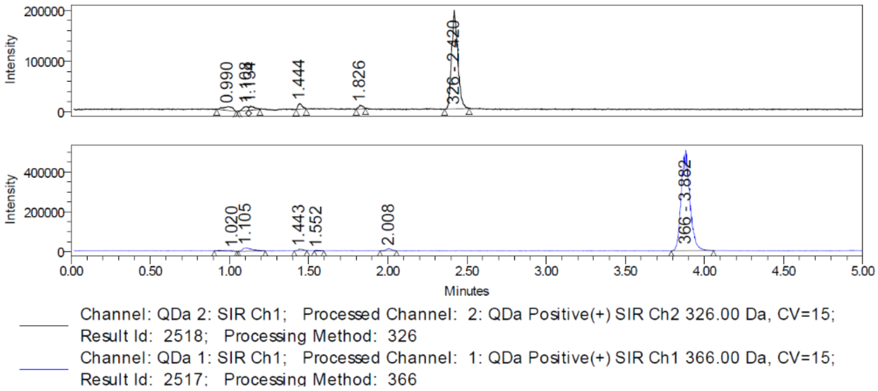

S48 Fig. UPLC-MS of T5144

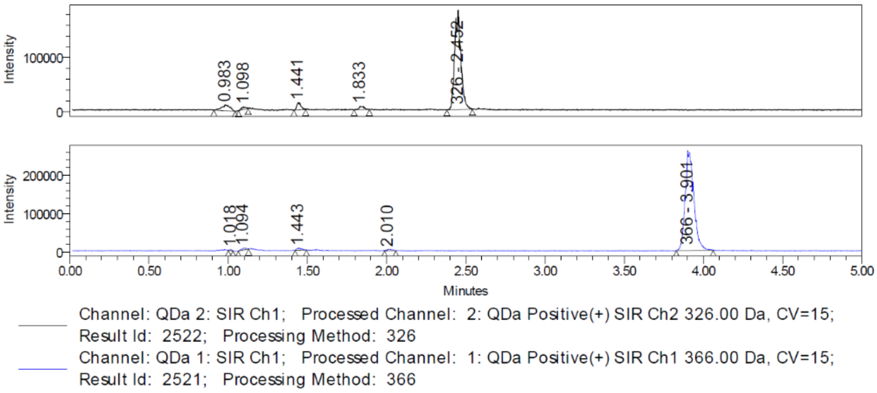

S49 Fig. UPLC-MS of T5387

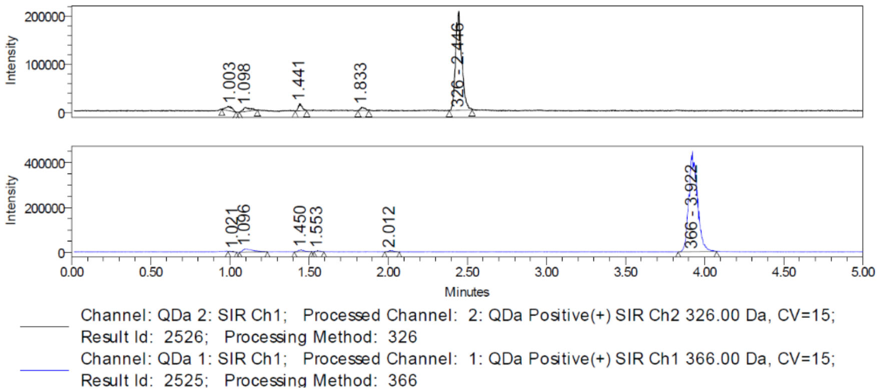

S50 Fig. UPLC-MS of T5389

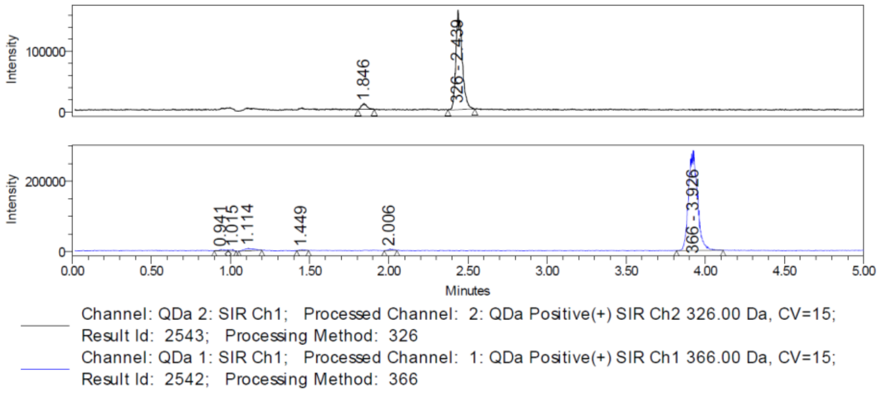

S51 Fig. UPLC-MS of T5391

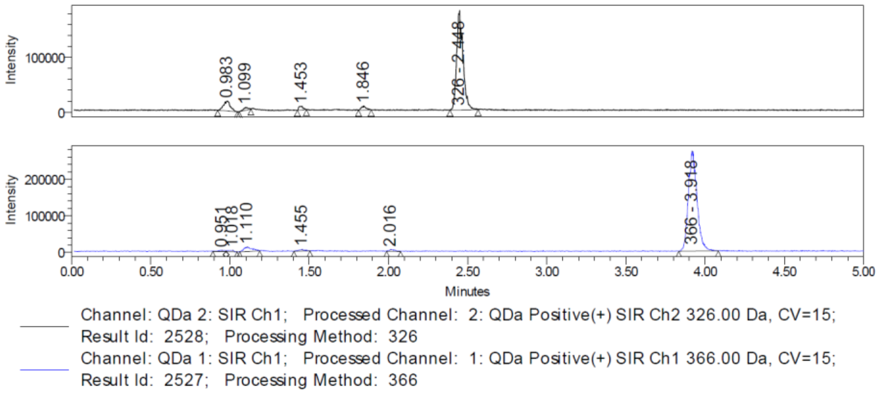

S52 Fig. UPLC-MS of T5392

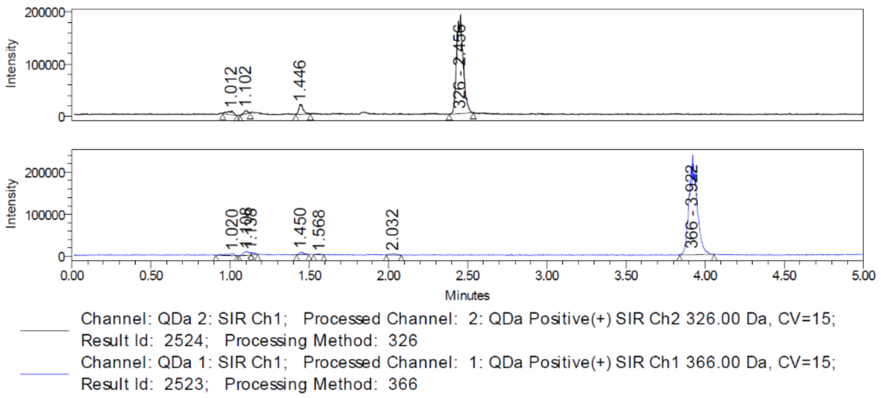

S53 Fig. UPLC-MS of T5394

5. The UPLC-MS results of samples with high percentages of adulterants

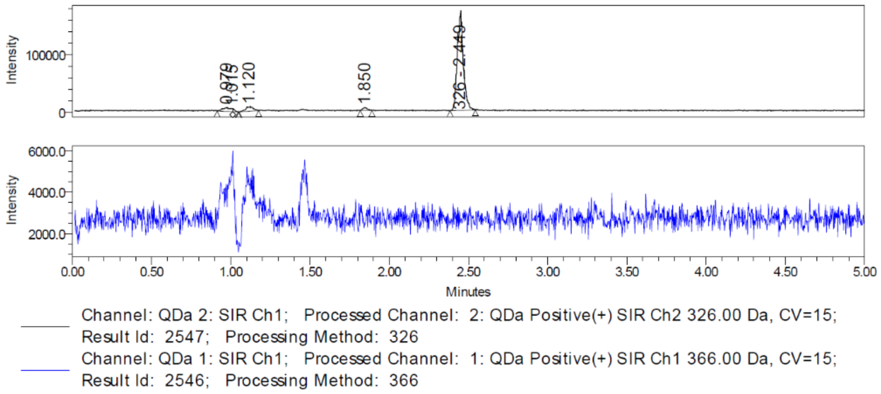

S54 Fig. UPLC-MS of T5060

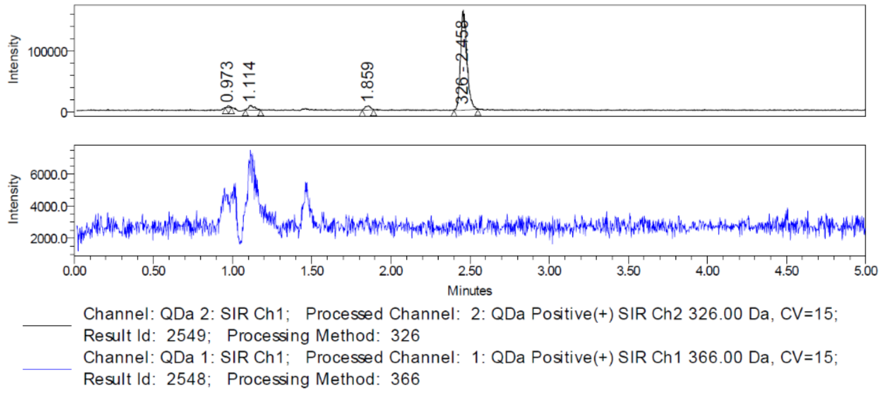

S55 Fig. UPLC-MS of T5062

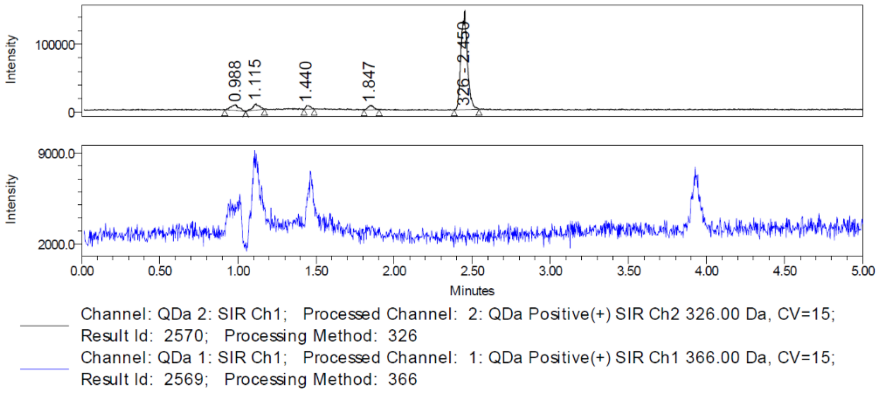

S56 Fig. UPLC-MS of T5079

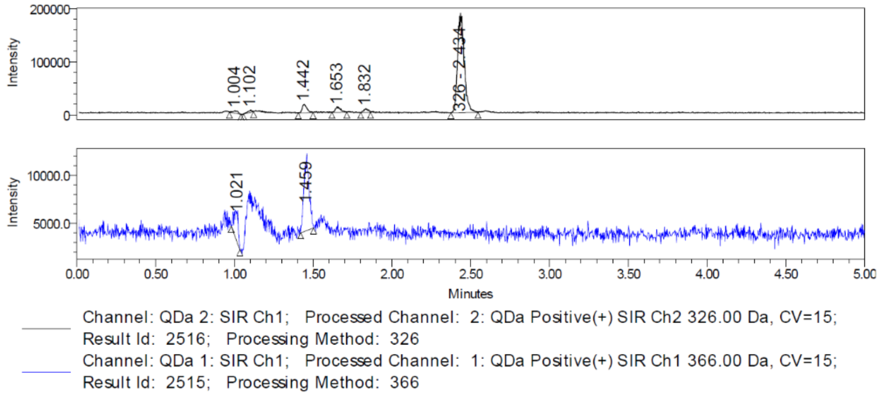

S57 Fig. UPLC-MS of T5138

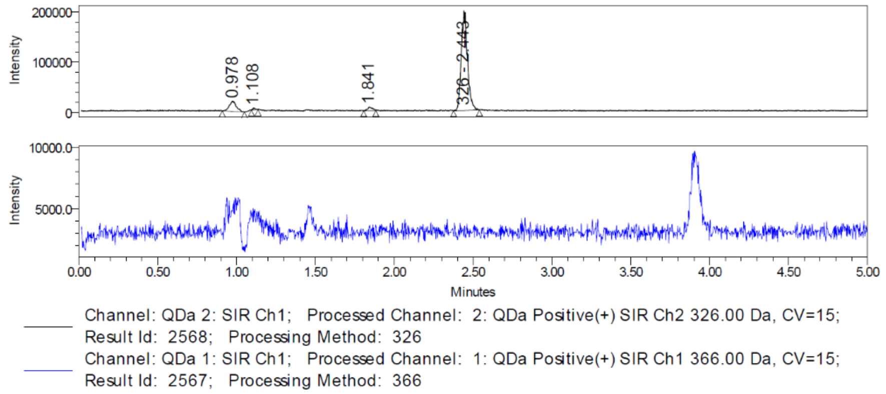

S58 Fig. UPLC-MS of T5141

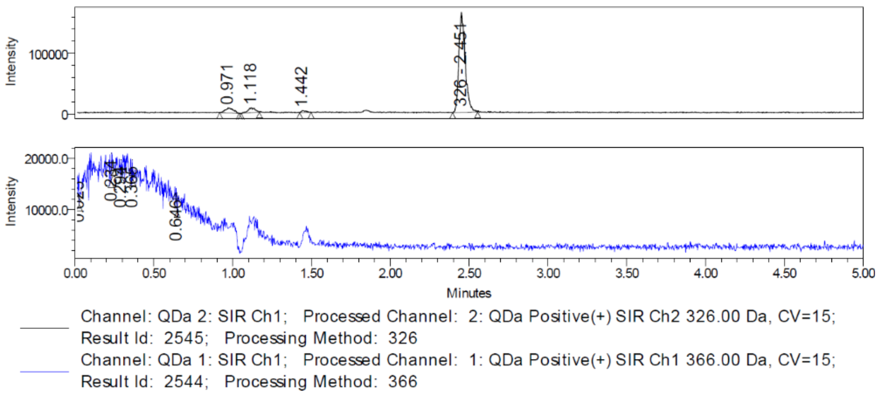

S59 Fig. UPLC-MS of T5388

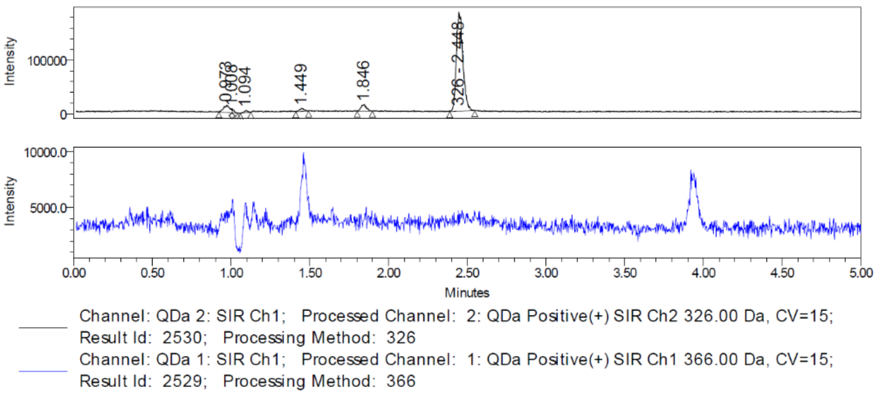

S60 Fig. UPLC-MS of T5390

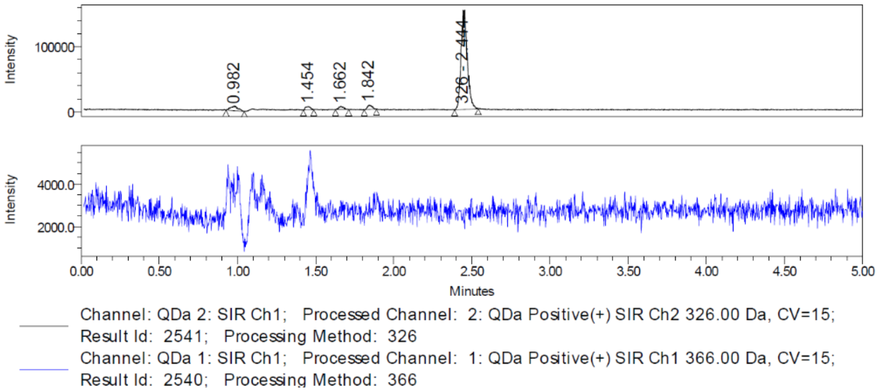

S61 Fig. UPLC-MS of T5393
