# Supplementary material for: Correction: Multi-methodological approach for the Quality assessment of Senecionis scandentis Herba (Qianliguang) in the herbal market
Source: PLoS One. 2025 Jul 29;20(7):e0329396. doi: 10.1371/journal.pone.0329396 (PMC12306740; doi:10.1371/journal.pone.0329396)
Supplement: S2 File — (PDF) [file pone.0329396.s002.pdf]

PLOS ONE

RESEARCH ARTICLE

Multi-methodological approach for the Quality assessment of Senecionis scandentis Herba (Qianliguang) in the herbal market

Hiu-Lam Ngai[1], Xiao Yang[2], Adrian Jun Chu[2], Rachel Harper[2], Alice B. J. E. Jacobsen[2], David Tai-Wai Lau[3], Ho-Yin Yu[4], Hung-Kay Lee[4]*, Pang-Chui Shaw[1,5]*

1 School of Life Sciences, The Chinese University of Hong Kong, Hong Kong, China, **2** Department of Microbiology, The Chinese University of Hong Kong Kong, Hong Kong, China, **3** Siu-Ying Hu Herbarium, School of Life Sciences, The Chinese University of Hong Kong, Hong Kong, China, **4** Department of Chemistry, The Chinese University of Hong Kong, Hong Kong, China, **5** LDS YYC R&D Centre for Chinese Medicine and Institute of Chinese Medicine, The Chinese University of Hong Kong, Hong Kong, China

* hklee@cuhk.edu.hk (H-KL); pcshaw@cuhk.edu.hk (P-CS)

Check for updates

Abstract

We set forth to assess the quality of an herbal medicine sold in Hong Kong called Qianliguang by employing a multi-methodological approach. The quality is set by its identity, chemical composition, and bioactivities, among others. Qianliguang (Senecionis scandentis Herba, *Senecio scandens* Buch.-Ham. ex D.Don) has known antibacterial properties. However, it is poisonous and overconsumption can result in liver damage. Eighteen Qianliguang samples were purchased from herbal shops at various districts in Hong Kong. Samples were first authenticated organoleptically. DNA barcoding at the *psbA-trnH*, *ITS2*, and *rbcL* loci was then conducted to confirm the species. HPLC-UV was performed to screen for the presence of the chemical compounds and to quantify the flavonoid hyperoside. UPLC-MS was used to quantify the amount of the toxic pyrrolizidine alkaloid (PA) adonifoline. Microdilution assay was performed to show the antibacterial effect on *Streptococcus aureus* and *S*. *pneumoniae*. Results showed that five samples were found to be substituted by species belonging to the genus *Lespedeza*; four samples were mixtures containing not only Qianliguang but also *Achyranthes aspera* L., *Lonicera confusa* DC., or *Solanum nigrum* L. HPLC-UV showed that only ten contained enough hyperoside to meet the standard requirement. In addition, nine samples had adonifoline that exceeded the toxicity standard requirement. In the microdilution assay, samples containing Qianliguang showed inhibition on *S*. *aureus* and *S*. *pneumoniae*, while among the five *Lespedeza* sp. samples the antibacterial effects on *S*. *aureus* were not detectable; only one sample showed inhibition to *S*. *pneumoniae*. Our study illustrated the necessity of using a multi-methodological approach for herbal medicine quality assessment. We also showed that Qianliguang samples in the Hong Kong market were either toxic or adulterated. It is therefore essential to improve the quality control of Qianliguang and probably other herbs in the herbal market.

OPEN ACCESS

Citation: Ngai H-L, Yang X, Chu AJ, Harper R, Jacobsen ABJE, Lau DT-W, et al. (2022) Multi-methodological approach for the Quality assessment of Senecionis scandentis Herba (Qianliguang) in the herbal market. PLoS One 17(4): e0267143. https://doi.org/10.1371/journal.pone.0267143

Editor: Branislav T. Šiler, Institute for Biological Research, University of Belgrade, SERBIA

Received: January 6, 2022

Accepted: April 1, 2022

Published: April 14, 2022

Copyright: © 2022 Ngai et al. This is an open access article distributed under the terms of the Creative Commons Attribution License, which permits unrestricted use, distribution, and reproduction in any medium, provided the original author and source are credited.

Data availability statement: All relevant data are within the manuscript and its Supporting Information files. The samples stored at the Institute of Chinese Medicine, The Chinese University of Hong Kong are available for reuse by others wishing to replicate the findings obtained in this study.

Funding: The authors received no specific funding for this work.

Competing interests: The authors have declared that no competing interests exist.

Introduction

Identity, chemical composition, and bioactivities are important indicators of herbal product quality. For achieving pharmaceutical efficacy it is essential to have the correct herb with the appropriate bioactivities. In our study we aim to demonstrate the necessity of adopting a multi-methodological approach on the evaluation of herbal quality by the exemplification of the quality assessment of Qianliguang in the Hong Kong market. Qianliguang was chosen as a target for this study because it could be easily obtained from the Hong Kong market despite its potential toxicity. So far there is no existing study on the authenticity and quality of Qianliguang in Hong Kong market, drawing onto the necessity of this investigation.

Qianliguang (Senecionis scandentis Herba) is a dried herb of *Senecio scandens* Buch.-Ham. ex D.Don [1]. It is mainly used for treating bacterial infections as it has a high flavonoid content [2,3]. It also has numerous phytochemicals, including alkaloids, phenolic acids, terpenes, volatile oils, carotenoids, and other trace elements [3]. However, its potential toxicity may be hepatotoxic and carcinogenic [4]. Its toxicity was attributed to the production of toxic metabolic pyrroles in the liver metabolism of pyrrolizidine alkaloids (PAs) [5].

Senecio plants have been used medicinally in different countries [1,6,7]. Adverse events associated with the use of these plants have been reported [6,7]. In South Africa, cases of infant mortality associated with the administration of an unidentified *Senecio* species that substituted the non-PA-producing *Senecio coronatus* have been reported [6]. The toxicity was caused by a high content of retrorsine-N-oxide [6]. In Europe, the consumption of a *Senecio-vulgaris*-containing herbal tea product resulted in several deaths [7]. *Senecio vulgaris* contained highly concentrated PAs including senecionine, seneciphylline, integerrimine, and retrorsine [8].

In Chinese medicine, Qianliguang is often used as a single pharmaceutical agent, or as a component of the hundreds of proprietary drugs such as Qianliguang tablets and Qianbai Biyan tablets [1].

Chinese Pharmacopoeia has provided the standard requirements for Qianliguang. Among these, the flavonoid hyperoside at 0.03% was established by Xiong and colleagues [9]. The toxic limit of adonifoline, a retronecine-type pyrrolizidine alkaloid at 0.004% was established [1]. The administration dose is 15 to 30 g [1]. Current analytical methods have been applied to assess Qianliguang's marker compounds and toxic PAs by HPLC-UV and HPLC-MS/MS [1,10–12]. These methods are specific but only focus on the concerned chemicals. Studies on other herbs have demonstrated the necessity of using multi-methods to ensure comprehensive evaluation [13,14].

It is important to ensure that the quality of Qianliguang on the market is meeting the standard for medical use. Therefore, we set forth to use Qianliguang to establish the multi-methodology approach for the comprehensive quality assessment. We aim to find the authenticity, bioactive compounds, toxicity, and potential pharmacological activities in samples obtained from eighteen districts in Hong Kong. Our multi-methodological approach allows high reliability and validity and helps inform of the quality of this herb in the Hong Kong market.

Materials and methods

Samples investigated

Samples were collected from eighteen herbal shops in Hong Kong. Each was the dried aerial part of what was claimed to be Qianliguang by the retailers. Each sample weighed 113 grams and was packed into an individually sealed plastic bag. The districts where these shops are located are listed on the website of the Home Affairs Department (https://www.gohk.gov.hk/eng/welcome/index.html). They were coded and stored in the Institute of Chinese Medicine, The Chinese University of Hong Kong (Table 1).

Table 1. Samples collected in this study.

#	Code	District
1	T5060	Shatin
2	T5061	Tsuen Wan
3	T5062	Sheung Shui
4	T5063	Sham Shui Po
5	T5064	Wong Tai Sin
6	T5079	Sheung Wan
7	T5135	Kwun Tong
8	T5138	To Kwa Wan
9	T5141	Wan Chai
10	T5144	North Point
11	T5387	Tai Po
12	T5388	Jordan
13	T5389	Yuen Long
14	T5390	Tuen Mun
15	T5391	Tsing Yi
16	T5392	Sai Kung
17	T5393	South District
18	T5394	Tung Chung

Note: The samples stored at the Institute of Chinese Medicine, The Chinese University of Hong Kong are available for reuse by others wishing to replicate the findings obtained in this study.

https://doi.org/10.1371/journal.pone.0267143.t001

Standard samples

Two pieces of leaves morphologically authenticated to be *Senecio scandens* by Dr. David Lau, Curator of the Shiu-Ying Hu Herbarium of The Chinese University of Hong Kong, were obtained for setting the biological standard for DNA barcoding. Three different lots of Qianliguang of Chinese Pharmacopoeia 2020 standard coded AZ21110503, AZ22011202, and AZ22021761 were obtained from Chengdu Alfa Biotechnology Co. Ltd. for setting the standards for HPLC-UV and UPLC-MS analyses.

Morphological authentication

Morphological authentication was performed by examining the organoleptic characteristics of the samples, followed by a comparison of the characteristics with the description of Qianliguang described in the Chinese Pharmacopeia 2020 [1] and the Hong Kong Baptist University School of Chinese Medicine Database [15]. Images that depict the organoleptic characteristics and morphological identities of samples are shown in the S1 File.

DNA authentication

Extraction. Three separate pieces of herb fragments were picked from each sample pack and then extracted three times to prepare three genomic DNA stocks for PCR amplification at *psbA-trnH*, *ITS2*, or *rbcL* DNA loci, which enabled the reproducibility of the species identification results. Twenty milligrams were cut for each fragment, washed with 75% ethanol to remove surface contaminants, and chopped into fine powder for DNA extraction.

DNA extraction was carried out using the Broad-spectrum Plant Rapid Genomic DNA Kit (Biomed, China) to obtain 50–100 μL of genomic DNA.

Polymerase chain reaction (PCR) amplification and sequence analysis. Universal primers at the *ITS2*, *psbA-trnH* and *rbcL* regions were used in the PCR. Primer sequences are listed in the S2 File. As the Qianliguang DNA may be degraded, species-specific primers were designed for the amplification of short amplicons when sample DNA could not be amplified using universal primers. DNA sequences from the *ITS2* regions were obtained from the NCBI GenBank for the design of species-specific diagnostic primers to differentiate *Senecio scandens* from closely related species based on the polymorphic sites (S3 File). Fifteen DNA sequences from the *ITS2* region were downloaded from GenBank, NCBI for sequence alignment and comparison with the biological standards of *Senecio scandens*. These sequences were of the highest similarity to the two biological standards. Sequences were aligned with ClustalW using BioEdit version 7.0.5.3 (10/28/05), and primers were designed by OligoAnalysis v3.1 (Integrated DNA Technologies, Coralville, IA, USA).

For a PCR setup, a reaction mixture of 30 μl included 3 μl of 10× PCR buffer [75 mM Tris, pH 8.8, 20 mM $(NH_4)_2SO_4$, 1.5 mM $MgCl_2$, 0.01% Tween 20], 2.4 μl of 2.5 mM deoxynucleotide triphosphates, 3 μl of 2.5 mM MgCl2, 1.5 μl of 10 μM of each primer, 1–4 uL of DNA sample, 0.2 μl of 5 U/μL Taq DNA polymerase, and an addition of double distilled Milli-Q (Merck Millipore) Ultrapure Water to compensate the remaining volume. The PCR amplification was performed using Veriti™ Thermal Cycler (Applied Biosystems, USA) or T100™ Thermal Cycler (Bio-Rad, USA). PCR amplification protocols of different time durations and temperatures of denaturation, annealing, and extension for amplification of different DNA loci were implemented (S2 File). DNA of *Hedyotis corymbosa* in a previous experiment was initially used for setting the PCR positive control. DNA of Qianliguang's fresh leaf (H2114 or H2126) was then adopted as the positive control in the subsequent PCR amplifications. A PCR negative control was set up with component mixture.

Gel electrophoresis was then performed for the blue-light visualization of PCR products using 1.5–3% of TAE gel stained with SYBR Safe DNA Gel Stain (Thermo Fisher Scientific). Gel purification was performed using a DNA gel purification kit (Biomed). Sanger sequencing was performed by BGI in Hong Kong. Obtained sequences were compared against available sequences in GenBank using Basic Local Alignment Search Tool nucleotide (BLASTn). Query sequences were identified to species level at 99–100% sequence similarity.

Chemical methods

Chemicals and reagents. The reference standard of hyperoside, crotaline and adonifoline were obtained from Standhill Technology Limited (Hong Kong, China). HPLC-grade methanol and acetonitrile were purchased from Fisher (Massachusetts, USA). Glacial acetic acid and absolute ethanol were obtained from EMSURE® (Darmstadt, Germany). Formic acid was obtained from LiChropur® (Darmstadt, Germany).

HPLC quantification of hyperoside. Two grams of each sample were ground into powder and extracted by heat under reflux using 50 mL of 75% methanol for an hour. The lost fluid was compensated accurately by 75% methanol in a volumetric flask with a 50 mL graduation mark. Two milligrams of hyperoside were mixed with 50 mL 75% methanol to make a standard solution.

HPLC was conducted using an Agilent 1260 LC instrument (Agilent, California, USA) equipped with autosampler, column temperature controller, DAD detector, and

quaternary pump. Forty microliters of each sample solution was injected into the instrument and eluted with an elution profile of 0 to 23 min of 85:15 = 0.2% glacial acetic acid:acetonitrile; 23 to 40 min of 75:25 = 0.2% glacial acetic acid:acetonitrile; and 40 to 55 min of 85:15 = 0.2% glacial acetic acid:acetonitrile, using an Agilent ZORBAX Eclipse XDB-C18 column (4.6 mm × 150 mm, 5 μm) at a column temperature of 25°C and a flow rate of 0.8 $mLmin^{-1}$. Forty microliters of hyperoside solution was injected into the Agilent 1260 LC system and eluted using the same elution protocol. Absorbance of the eluate was monitored at 360 nm. The hyperoside contained in each sample solution was quantitated by comparing peak areas at the elution timepoint of hyperoside. A standard curve plot based on the peak areas produced by the injection of different concentrations of hyperoside was used for the quantification of hyperoside in the sample solution. According to the Chinese Pharmacopoeia, each sample should contain more than 0.03% of hyperoside.

UPLC-MS quantification of adonifoline. An internal standard solution consisting of 0.2 μg crotaline in 1 mL 0.5% formic acid was used for the determination of the calibration factor. For each sample, 0.2 g of herb powder was mixed with 50 ml of 0.5% formic acid and extracted by ultrasonic treatment for 40 min. The solution was filtered and compensated for liquid loss with 0.5% formic acid. Two milliliters of the extract filtrate, 1 mL of internal standard solution, and 2 mL of 0.5% formic acid was added to the graduation mark of a 5 mL volumetric flask to make each of the sample solutions. The reference standard solution consisted of 2 mL of adonifoline solution (0.101 μgmL^{-1}), 1 mL of crotaline solution, and 2 mL of 0.5% formic acid.

Five microliters of each sample solution and 5 μL of reference standard solution were injected into a Waters Xevo G2-XS QTOF mass spectrometer (Waters Co., Milford, MA, USA) connected to the UPLC system through an electrospray ionization (ESI) interface, and eluted with an elution profile of 0.1% formic acid:acetonitrile = 90:10 at a flow rate of 0.3 $mLmin^{-1}$, using an ACQUITY UPLC BEH C18 Column (130Å, 2.1 mm X 150 mm, 1.7 μm or 130Å, 2.1 mm X 100 mm, 1.7 μm). The detection was set at Qda Positive(+) SIR Ch1 366.00 Da and Qda Positive(+) SIR Ch2 326.00 Da, both at CV = 15 for the detection of adonifoline and the internal standard crotaline, respectively. The percentage of adonifoline was quantitated by the equation $A_x/C_x = F^* A_s/C_s$, where A refers to area and C refers to concentration, A_s and C_s are those of internal standard crotaline, and A_x and C_x are those of the sample solution. The response factor F was used for the calculation of the percentage of adonifoline contained in each sample solution compared to the reference standard. According to the Chinese Pharmacopoeia 2020, each sample should contain no more than 0.004% of adonifoline.

Determination of extract antimicrobial activity by broth microdilution assay

Ten grams of each sample were pulverized, followed by heating under reflux for one hour using 100 mL 75% methanol to obtain the extract. The crude extract was then evaporated into residues, lyophilized and resuspended into 2 gmL^{-1} of each extract using 10% DMSO-double distilled water.

The antimicrobial activities of all extracts used in this study were assessed by the broth microdilution method, adapted from guidelines as stipulated by the Clinical & Laboratory Standards Institute (CLSI M100-Ed29) (2021). Test media used were cation-adjusted Mueller-Hinton broth (CA-MHB) (CM0405, Oxoid, Basingstoke, United Kingdom), and Brain Heart Infusion (BHI) (CM1135, Oxoid, Basingstoke, United Kingdom) for streptococcal

strains. Fresh colonies of *Staphylococcus aureus* ATCC® 25923™ and 29213™ were obtained from glycerol stocks plated onto LB agar, and *Streptococcus pneumoniae* ATCC® 49619™ plated onto Columbia horse blood agar (Thermo Fischer, Waltham, Massachusetts, USA). Following overnight incubation at 37°C, each bacterial inoculum was prepared by looping a single colony, resuspending it in fresh medium, and calibrated to 0.5 McFarland with a DensiCheck Plus densitometer (BioMérieux, Marcy-l'Étoile, France), before adjusted to a final concentration of ~5 × 10^5 CFU/mL in each well. Two-fold serial dilutions were performed for the extracts tested on 96-well plates, ranging from 51.2 mg/mL to 0.05 mg/mL. The antibiotic vancomycin (Sigma-Aldrich, St. Louis, Missouri, United States) also served as a positive control, ranging from 64 μg/mL to 0.0625 μg/mL. Extract-free 10% DMSO-double distilled water was also included for each strain to serve as solvent toxicity control, alongside untreated negative control groups.

The final inoculum of each strain was added to plates pre-loaded with serially diluted extracts and control drugs, followed by incubation at 37°C for 20 hrs. Results were recorded and the MIC (minimum inhibitory concentration) for each treatment group was defined as the lowest concentration of antimicrobial compounds used with no visible growth in well plates.

Results

Morphological authentication

Images of sample fragments and organoleptic characteristics of Qianliguang are shown in the S1 File. Fragments without these characteristics were regarded as non-Senecionis-scandentis-Herba.

There were different percentages of adulterations, ranging from 0 to 100%. The percentage of adulterations or substitutions of each sample is shown in Table 2. Five samples were almost completely adulterated by *Lespedeza* sp., with woody and fibrous stems and stiff, flattened bristles showing they were not Qianliguang. Three samples were mixtures with significant percentages of adulterants of 92%, 86%, and 28%. Characteristics of the adulterants are described in the S1 File. A major adulterant found was *Achyranthes aspera*. It had pilose quadrangular stems with slightly enlarged nodes; its total peduncle was angular, stout, hard, densely white appressed or pubescent; and it had sparse flowers of 3–4 mm long. Some samples contained small amounts of adulteration of unidentified species.

Authentication by DNA barcoding

DNA barcoding results at the *ITS2*, *psbA-trnH* loci using BLAST nucleotide revealed the species identities (Table 2). Nine samples were identified to be Qianliguang. Five samples were *Lespedeza* sp. Four samples were mixtures containing both Qianliguang and other plants, including *Achyranthes aspera*, *Lonicera confusa*, or *Solanum nigrum*. Results of the sequence alignment are shown in the S4 File. The results of DNA concentration and purity as A260/A280 are shown in the S5 File.

The overall authentication results are summarized in Table 2.

HPLC-UV quantification of hyperoside

The HPLC method was based on Chinese Pharmacopoeia 2020 [1] with slight modification for separating the major chemical peaks. Hyperoside was detected at around 26.0–27.0 min (S6 File).

Table 2. Summary of results.

Code	DNA loci	DNA barcode identification	Morphological identification	% Adulterant determined by mass (g) morphologically	% Hyperoside	% Adonifoline	MIC (mg/mL)		
							SAUR 25923	SAUR 29213	SPNE 49619
T5060	*ITS2*	*Lespedeza* KY174540.1; KY174742.1	*Lespedeza* sp.	100% [a]	0	0	>51.2	>51.2	>51.2
T5061	*ITS2*	*Senecio scandens*: MH711703.1	*Senecio scandens*	2.0% [b]	0.041%	0.00728%	25.6	25.6	0.8
T5062	*rbcL*	*Lespedeza* KY174540.1; KY174742.1	*Lespedeza* sp.	100% [a]	0	0	>51.2	>51.2	>51.2
T5063	*ITS2*	*Senecio scandens*: MH711703.1	*Senecio scandens*	0.5% [b]	0.030%	0.00516%	>51.2	25.6	3.2
T5064	*ITS2*	*Senecio scandens*: MH711703.1	*Senecio scandens*	0.2% [b]	0.036%	0.00353%	25.6	12.8	1.6
T5079	*ITS2*	*Lespedeza* KY174540.1; KY174742.1	*Lespedeza* sp.	100%[a]	0	0	>51.2	>51.2	>51.2
T5135	*ITS2*	*Senecio scandens*: MH711703.1	*Senecio scandens*	0.1% [b]	0.062%	0.00577%	25.6	25.6	0.8
T5138	*ITS2*	*Senecio scandens*: AF459932.1	*Senecio scandens* & *Achyranthes aspera*	28.0% [c]	0.006%	0	25.6	>51.2	1.6
T5141	*ITS2*	*Lespedeza* KY174540.1; KY174742.1	*Lespedeza* sp.	100% [a]	0	0	>51.2	>51.2	1.6
T5144	*ITS2*	*Senecio scandens*: MH711703.1	*Senecio scandens*	0.1% [b]	0.041%	0.00983%	3.2	12.8	1.6
T5387	*ITS2*	*Senecio scandens*: AF459932.1; MH711703.1	*Senecio scandens*	0.1% [b]	0.053%	0.00539%	12.8	25.6	1.6
	psbA-trnH	*Senecio scandens*: KX346924.1; KX346999.2	*Senecio scandens*						
T5388	*ITS2*	*Lespedeza* KY174540.1; KY174742.1	*Lespedeza* sp.	100% [a]	0	0	>51.2	>51.2	>51.2
T5389	*ITS2*	*Senecio scandens*: MH711703.1	*Senecio scandens*	0	0.067%	0.00816%	25.6	25.6	1.6
T5390	*ITS2*	*Senecio scandens*: MH711703.1 *Achyranthes aspera*: MG730938.1	*Senecio scandens* & *Achyranthes aspera*	92.0% [c]	0	0	25.6	25.6	0.8
	psbA-trnH	*Achyranthes aspera*: GQ435413.1							
T5391	*ITS2*	*Senecio scandens*: MH711703.1	*Senecio scandens*	0	0.019%	0.00672%	25.6	25.6	0.8
	psbA-trnH	*Senecio scandens*: KX346924.1							
T5392	*ITS2*	*Senecio scandens*: MH711703.1	*Senecio scandens*	0	0.038%	0.00562%	25.6	25.6	1.6
T5393	*ITS2*	*Senecio scandens*: AF459932.1	*Senecio scandens* & *Achyranthes aspera*	86.0% [c]	0	0	12.8	25.6	0.8
	psbA-trnH	*Achyranthes aspera*: GQ435413.1							

(Continued)

Table 2. (Continued)

Code	DNA loci	DNA barcode identification	Morphological identification	% Adulterant determined by mass (g) morphologically	% Hyperoside	% Adonifoline	MIC (mg/mL)		
							SAUR 25923	SAUR 29213	SPNE 49619
T5394	*ITS2*	*Senecio scandens*: MH711703.1; *Solanum nigrum*: KY700485.1	*Senecio scandens*	2.0% [b]	0.032%	0.00412%	25.6	25.6	1.6
	psbA-trnH	*Senecio scandens*: KX346924.1							
							Vancomycin (μg/mL) Control		
							2	1	0.5

Note

"a": The adulterants were *Lespedeza* sp.

"b": The adulterants were unidentified species.

"c": The adulterants were *Achyranthes aspera*.

Conclusion: Only sample T5064 fulfilled all the requirements.

https://doi.org/10.1371/journal.pone.0267143.t002

A stock solution of hyperoside (40 μg mL^{-1}) was prepared in 75% methanol. Standard calibration solutions (10 μg mL^{-1}, 20 μg mL^{-1}, 30 μg mL^{-1}, and 40 μg mL^{-1}) for the assessment of linearity were prepared from this stock solution. The solution was filtered through a 0.45 μm nylon filter prior to injection into the HPLC system. A standard curve of hyperoside was made by linear regression of the data of peak area versus drug concentration (S6 File). The calibration curve was found to be linear with the equation of $y = 2095.8x - 72.738$, with a correlation coefficient of $R^2 = 0.999$. The peak area of each injection, and the mean and standard deviation of the three replications are summarized in the S6 File. The amount of hyperoside in each sample was calculated with reference to the hyperoside standard curve (Table 2). Profiles of three standard samples of ChP 2020 standard were set up as references (S34-S36 Fig in S6 File). The profiles were similar and hyperoside was detectable in all three samples at 26.0 min. According to the Chinese Pharmacopoeia, hyperoside content should not be less than 0.03% of the sample. Only ten out of the eighteen samples met the standard requirement for the percentage of hyperoside (Table 2). Samples having 98–100% of Qianliguang fulfilled the Chinese Pharmacopoeia 2020 standard requirement of 0.03%. Representative HPLC profiles of samples are shown in Fig 1. The profile of each sample is shown in S6 File.

UPLC-MS quantification of adonifoline

In the chemical standard mixture and commodity samples containing adonifoline and crotaline, adonifoline was detected at the retention time of 3.80–3.90 min and crotaline was detected at the retention time of 2.40–2.50 min using an ACQUITY UPLC BEH C18 Column (130Å, 2.1 mm X 100 mm, 1.7 μm). In the Qianliguang standard samples (ChP 2020) containing adonifoline and crotaline, adonifoline was detected at the retention time of 2.40 min and crotaline was detected at the retention time of 1.70 min with an ACQUITY UPLC BEH C18 Column (130Å, 2.1 mm X 150 mm, 1.7 μm). The profiles are shown in the S7 File. According to the Chinese Pharmacopoeia 2020, each sample solution should contain no more than 0.004% adonifoline. The adonifoline content in standard samples met the requirement (S7 File). Most commodity samples (nine out of ten, except T5064) containing 98–100% of *Senecio scandens* exceeded the toxicity standard requirement. Samples containing a substantial

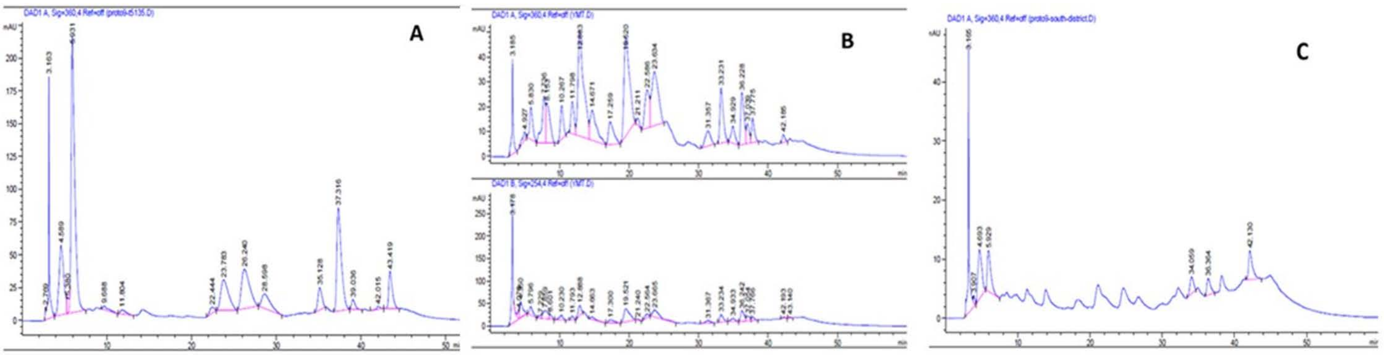

Fig 1. Representative HPLC profiles of samples. (a) Genuine Qianliguang, (b) substituted by plants of *Lespedeza* genus, (c) substituted by *Achyranthes aspera*. Signals of (b) were detected at both 360 nm (upper) and 254 nm (lower) to confirm the lack of hyperoside detection at 26.0–27.0 min. Signals of (a) and (c) were detected at 360 nm.

https://doi.org/10.1371/journal.pone.0267143.g001

amount (28%-100%) of adulterants had undetectable adonifoline (S7 File). The quantification results are shown in Table 2.

Antimicrobial effects of extracts against bacterial pathogens

Samples containing Qianliguang all showed potent inhibitory effects on *S*. *pneumoniae* ATCC® 49619™, and detectable inhibitory effects on either of the *S*. *aureus* strains ATCC® 25923™ or ATCC® 29213™. Alternatively, samples not containing Qianliguang did not show any inhibitory effect on either of the *S*. *aureus* strains. Only one sample not containing Qianliguang showed an inhibitory effect on *S*. *pneumoniae* ATCC® 49619™. Samples not containing Qianliguang were all *Lespedeza* sp. (authenticated by DNA and morphological identification). These results showed that samples of Qianliguang were consistent for their inhibitory effects on pathogenic bacteria such as *S*. *aureus* and *S*. *pneumoniae*. A sample (T5141) not containing Qianliguang showed detectable antibacterial effect on *S*. *pneumoniae* ATCC® 49619™. Furthermore, samples of mixtures containing Qianliguang and *Achyranthes aspera* (T5138, T5390, and T5393 as authenticated by DNA and morphological identification) showed similar antibacterial effects as the samples that only contained Qianliguang, suggesting that *Achyranthes aspera* might elicit synergistic antibacterial effects together with Qianliguang. Past studies also showed that *Achyranthes aspera* has antibacterial effects on *S*. *aureus* [16].

Discussion

The implication of unexpected findings in our study

Samples were expected to contain the correct species, with similar chemical profiles and low toxicity. Nevertheless, unexpected findings were observed.

Our results showed that the quality of Qianliguang in Hong Kong is highly varied. First, the percentage of adulterants in the samples ranged from 0 to 100%. Second, eight samples with significant adulteration had an undetectable or sub-standard amount of hyperoside. Nine out of the ten samples with detectable adonifoline exceeded the toxicity limit in the Chinese Pharmacopoeia 2020. Third, the bioactivities of all samples containing Qianliguang inhibited *S*. *aureus* and *S*. *pneuomniae* strains, while samples of *Lespedeza* sp. showed no inhibition on the *S*. *aureus* strains.

Overall, our study illustrated the power of using a multi-methodological approach for authentication and quality assessment. We have shown there is a lack of quality control of Qianliguang in the market. Among the 18 samples tested, only one sample fulfilled all the criteria stated in the Chinese Pharmacopoeia 2020 [1]. Our study newly revealed serious quality problems in Qianliguang and showed the necessity to improve the regulation of the herbal trade.

There are recent studies on authenticity and quality problems in medicinal plant materials in various places relevant to our study [13,14,17–19]. A DNA-based study on the authentication of 5,957 commercial herbal products found that globally 27% of herbal products were adulterated [17]. These observations imply the global substandard regulation of herb quality.

In China, unintentional adulteration was found in common unexpensive herbs. The reasons include confusing names [20], packaging mistakes [17], and misidentification due to similar morphology [3]. Yik and colleagues found confused *Hedyotis* products in Hong Kong [21]. Deliberate adulteration was found in more expensive herbs. Lo and colleagues found that half of the Placenta Hominis samples in Hong Kong and Taiwan markets were either from non-human origin or a mixture of Placenta Hominis and starch filler [22]. The adulterants include contaminants, substitutes, fillers, or unlabeled species. The problem of adulteration has a significant impact on the safety and quality of herbal products.

Our study also found potential reasons for the adulteration of Qianliguang in the Hong Kong market. For instance, *Lespedeza* sp. was found to have a number of common names, including Qianliguang [23,24]. This probably confused the retailers, resulting in the wrong dispensary.

Three samples contained a mixture of *Achyranthes aspera* and *Senecio scandens*. This revealed a lack of good agriculture practice or an improper harvest of the herb. *Achyranthes aspera* is an invasive species, found in the wild and cohabiting with other plants [25]. *Senecio scandens* is a plant that climbs and reclines on surfaces during its growth [26]. The mixtures might suggest the cohabitation and the co-harvest of the two herbs.

Imprecise harvesting can result in cohabitation and collection of mixed species. People may mislabel and dispense the wrong species in herb shops. To implement quality control at different stages of the supply chain, farms should employ good agricultural practices (GAP), contents in the herb products should be tested and recorded in the processing stage, and consumer records should be traceable at the point of sale.

A way to rectify the problem is to properly record the chain of production, from herb growth to the retail of products. HerBChain, a block chain platform for record and information dissemination, was recently established for such purposes [27]. As we found that Qianliguang samples were highly varied, other Chinese medicinal materials might also have similar problems and a multi-methodological approach should be adopted to assess them, with an aim to identify the problems in an evidence-based manner.

Analytical methods for the quality control of herbal materials

Chemicals in herbal products are always used for authentication and quality assessment [1,28]. The Chinese Pharmacopoeia provides protocols for the chemical assessment of the enlisted herbs [1]. Nonetheless, herbal quality assessment based solely on chemicals may not be sufficient. According to the World Health Organization (WHO), the choice of methods for herbal product quality assessment is determined with due consideration on the level of substance detection and the herbal material matrix used for testing (e.g., seeds containing oils) [29]. The WHO has emphasized that some of the chemical limits are too diverse and lack international consensus to be set due to the huge variations across herbal products [29]. The

WHO has also suggested that initial screening of herb purity and quality can be done by morphological investigation as fragments that are inconsistent or of exceptionally poor quality can be identified quickly [29]. However, morphological assessment can be subjective, and conclusion can be different across assessors. Furthermore, herbal products with no suitable chemical or biological assay can only be assessed by the amount of total extractable matters [29]. DNA authentication methods such as DNA barcoding can help determine the biological components [30]. Multiple assessment methods should be adopted for comprehensively assessing herbal quality and facilitating the formation of solutions to rectify underlying problems related to sub-standard herbal product quality.

The strength and limitation of adopting a multi-methodological approach

Researchers have previously authenticated *Senecio* species by comparing the chemical profiles or specific chemicals in freshly collected herbs [2,28]. In this study, we showed the significance of using multiple methodologies for the evaluation of herbal quality. Morphological identification, DNA barcoding, and the chemical analyses of bioactive and toxic compounds, coupled with the antibacterial assay, produced accurate authentication and quality assessment in this study. This revealed that key organoleptic characteristics could be similar between Qianliguang and the adulterant *Achyranthes aspera*. DNA barcoding could overcome the difficulty in morphological authentication; using individual herb fragments confirms the observation from the examination of organoleptic characteristics. The adoption of DNA barcodes at the *ITS2*, *psbA-trnH*, and *rbcL* regions accorded with the recommendation by the International Barcode of Life Consortium (iBOL) [31] allowed precise and confirmative species identification.

Comparing the discriminatory properties of DNA barcodes in our study, the *rbcL* region has a conservative evolutionary rate and a consistency in length [32]. However, it only provided the differentiation at the Family level. The *psbA-trnH* intergenic spacer consists of a high frequency of mononucleotide repetition, which impedes bidirectional sequencing [33,34]. The *ITS2* is a short subregion of *ITS* [35]. It was easier to amplify than *psbA-trnH*, producing the most sequences amongst the three regions in our study. Gao and colleagues suggested that *ITS2* is the optimal DNA barcode for the Family Asteraceae [36], further confirming that *ITS2* is a suitable DNA barcode for the molecular authentication of Qianliguang.

In addition, chemical fingerprinting by HPLC allowed qualitative and quantitative analyses of compounds. The chemicals differed across samples of different species, suggesting potential differences in their pharmacological activities. The quantification of flavonoid by HPLC-UV and the antibacterial screening provided effective evaluation on the quality of Qianliguang. Furthermore, UPLC-MS showed the amount of toxic compound in most of the genuine samples exceeded the regulatory limit, demonstrating the importance of assessing the known toxic compound in an herb.

The assessment of toxicity in Qianliguang

Qianliguang is potentially toxic, and the assessment of its toxicity is essential [4]. Cases of toxicity in relation to the use of *Senecio* species have been reported [6,7]. The toxicity was attributed by the production of toxic metabolic pyrroles by the liver metabolism of pyrrolizidine alkaloids (PAs) [5,37]. There are three types of representative PAs: retronecine-, otnecine-, and platynecine-groups. The most toxic ones are retronecine-type PAs with 1,2-unsaturated structures such as adonifoline. PAs are hydrolyzed into necine and necic acid in the liver. However, the branched chain of necic acid causes steric hindrance, leading to the generation of metabolic pyrroles by the catalytic action of microsomal mixed function oxidases. The strong electrophilicity of metabolic pyrroles allows their strong affinities to

enzymes, proteins, DNA, and RNA. These conjugations interfere with cell mitosis, causing liver toxicity, mutagenesis, and carcinogenicity [5]. PAs are present in most of the plants of the Genus *Senecio* [6,7,28,37]. Various recent research has been conducted on the assessment of PAs in Qianliguang [11,12,37]. Retronecine-type pyrrolizidine alkaloids (RET-PAs) with 1,2-unsaturated structures are the most toxic among the three groups of pyrrolizidine alkaloids (retronecine-, otnecine- and platynecine-groups) [11]. Adonifoline is of the highest concentration among detectable RET-PAs in Qianliguang [11]. The quantification of adonifoline in our study at the limit of 0.004% was accorded with the Chinese Pharmacopoeia 2020 [1]. We found adonifoline consistently detectable in our study. Our study may be elaborated with the quantification of other RET-PAs such as seneciphylline [37].

Future direction

We established the multi-methodological approach to assess the quality of Qianliguang. Further refinement and automation of the protocols and the generation of a standard operating procedure (SOP) will lead to its use as a routine quality control tool on Qianliguang.

Supporting information

S1 File. A summary of organoleptic characteristics and morphological identities.
(PDF)

S2 File. PCR amplification protocols.
(PDF)

S3 File. Species-specific primer design.
(PDF)

S4 File. Sequence alignment at *ITS2*, *psbA-trnH*, and *rbcL* DNA regions.
(PDF)

S5 File. DNA concentration and purity of samples.
(DOCX)

S6 File. Chromatographs of HPLC-UV for the quantification of hyperoside.
(PDF)

S7 File. Results of UPLC-MS.
(PDF)

Author contributions

Conceptualization: Hiu-Lam Ngai, David Tai-Wai Lau, Pang-Chui Shaw.

Data curation: Hiu-Lam Ngai.

Formal analysis: Hiu-Lam Ngai.

Investigation: Hiu-Lam Ngai, Adrian Jun Chu, Rachel Harper, Alice B. J. E. Jacobsen, David Tai-Wai Lau, Ho-Yin Yu.

Methodology: Hiu-Lam Ngai, Xiao Yang, Adrian Jun Chu, Rachel Harper, Alice B. J. E. Jacobsen, David Tai-Wai Lau, Ho-Yin Yu, Hung-Kay Lee, Pang-Chui Shaw.

Project administration: Hiu-Lam Ngai.

Resources: Xiao Yang, David Tai-Wai Lau, Hung-Kay Lee, Pang-Chui Shaw.

Software: Hiu-Lam Ngai.

Supervision: David Tai-Wai Lau, Hung-Kay Lee, Pang-Chui Shaw.

Validation: Xiao Yang, Hung-Kay Lee, Pang-Chui Shaw.

Visualization: Hiu-Lam Ngai.

Writing – original draft: Hiu-Lam Ngai.

Writing – review & editing: Xiao Yang, Adrian Jun Chu, David Tai-Wai Lau, Hung-Kay Lee, Pang-Chui Shaw.

References

1. The Committee of Pharmacopeia of the People's Republic of China. Pharmacopeia of the People's Republic of China. 11th ed. Beijing: Chemical Industry Press; 2020.
2. Yang XJ, Yang L, Xiong AZ, Li DX, Wang ZT. Authentication of Senecio scandens and S. vulgaris based on the comprehensive secondary metabolic patterns gained by UPLC-DAD/ESI-MS. J Pharm Biomed Anal. 2011;56: 165–172. https://doi.org/10.1016/j.jpba.2011.05.004 PMID: 21664784
3. Wang D, Huang L, Chen S. Senecio scandens Buch.-Ham.: A review on its ethnopharmacology, phytochemistry, pharmacology, and toxicity. J Ethnopharmacol 2013;149(1): 1–23. https://doi.org/10.1016/j.jep.2013.05.048 PMID: 23747644
4. Hospital Authority Toxicology Reference Laboratory. Atlas of Poisonous Plants in Hong Kong—A Clinical Toxicology Perspective. Senecio scandens. 2018. Available from: https://www3.ha.org.hk/toxicplant/en/senecio_scandens.html.
5. Wu B, Wu LJ. Research Progress on the Chemical Constituents of Senecio Plants. China Journal of Chinese Materia Medica. 2003;28(2): 97–100. PMID: 15015275
6. Van Schalkwyk FJ, Stander MA, Nsizwane M, Mathee A, Van Wyk BE. Fatal pyrrolizidine alkaloid poisoning of infants caused by adulterated Senecio coronatus. Forensic Sci Int. 2021;320(110680): 1–7. https://doi.org/10.1016/j.forsciint.2020.110680 PMID: 33461004
7. Xiong AZ, Fang LX, Yang X, Yang F, Qi M, Kang H, et al. An application of target profiling analyses in the hepatotoxicity assessment of herbal medicines: Comparative characteristic fingerprint and bile acid profiling of Senecio vulgaris L. and Senecio scandens Buch.-Ham. Anal Bioanal Chem. 2014;406(29): 7715–7727. https://doi.org/10.1007/s00216-014-8175-z PMID: 25234307
8. Xiong AZ, Yang L, Ji LL, Wang ZY, Yang XJ, Chen Y, et al. UPLC-MS based metabolomics study on Senecio scandens and S. vulgaris: An approach for the differentiation of two Senecio herbs with similar morphology but different toxicity. Metabolomics. 2012;8(4): 614–623. https://doi.org/10.1007/s11306-011-0354-8
9. Xiong AZ, Yang L, Yang XJ, Qi M, Zong LL, Wang CH, et al. RP-HPLC quantitation of hyperoside in medicinal planta Senecionis Scandentis. Chinese Journal Pharmacology Analysis. 2010;30: 936–939.
10. Yong W, Zhong GH, Yuan MM, Xie EL. Simultaneous determination of ten chemical compositions in the aerial parts of Senecio scandens by HPLC. Chin J Pharm Anal. 2021;41(10): 1724–1728.
11. Wang Y, Li Y, Cang S, Cai Q, Xu H, Wang Y, et al. Qualitative and quantitative analysis of pyrrolizidine alkaloids for the entire process quality control from Senecio scandens to Senecio scandens-containing preparations by high performance liquid chromatography-tandem mass spectrometry. J Mass Spectrom. 2020;55(10): e4532. https://doi.org/10.1002/jms.4532 PMID: 32662582
12. Yang M, Tan D, Lu A, Qin L, Wang C, Ling H, et al. Identification and Simultaneous Determination of the Main Toxic Pyrrolizidine Alkaloids in a Compound Prescription of Traditional Chinese Medicine: Qianbai Biyan Tablet. Int J Anal Chem. 2021;5209618: 1–13. https://doi.org/10.1155/2021/5209618 PMID: 34539789
13. Yu N, Wei YL, Zhu Y, Zhu N, Wang YL, Zhang HP, et al. Integrated approach for identifying and evaluating the quality of Marsdenia tenacissima in the medicine market. PloS One. 2018;13(4): e0195240. https://doi.org/10.1371/journal.pone.0195240 PMID: 29624609
14. Gesto-Borroto R, Cardoso-Taketa A, Yactayo-Chang JP, Medina-Jiménez K, Hornung-Leoni C, Lorence A, et al. DNA barcoding and TLC as tools to properly identify natural populations of the Mexican medicinal species Galphimia glauca Cav. PloS One. 2019;14(5): e0217313. https://doi.org/10.1371/journal.pone.0217313 PMID: 31136619
15. Hong Kong Baptist University–School of Chinese Medicine. Senecio scandens Buch.-Ham. Ex D. Don. Medicinal Plant Images Database. 2022. Available from: http://libproject.hkbu.edu.hk/was40/detail?channelid=1288&lang=en&record=1&searchword=(Senecio)&sortfield=%20name_formal_latin_sort.

16. Naidu PL, Kumar KK, Kumar CM, Gunesh G, Rao MN. Antimicrobial activity of Achyranthes aspera. Biosci Biotechnol Res Asia. 2006;3(1): 171–174.
17. Ichim MC. The DNA-based authentication of commercial herbal products reveals their globally widespread adulteration. Front Pharmacol. 2019;10(1227): 1–9. https://doi.org/10.3389/fphar.2019.01227 PMID: 31708772
18. Santhosh Kumar JU, Krishna V, Seethapathy GS, Ganesan R, Ravikanth G, Shaanker RU. Assessment of adulteration in raw herbal trade of important medicinal plants of India using DNA barcoding. 3 Biotech. 2018;8(3): 1–8. https://doi.org/10.1007/s13205-018-1169-3 PMID: 29479511
19. Yanaso S, Phrutivorapongkul A, Hongwiset D, Piyamongkol S, Intharuksa A. Verification of Thai ethnobotanical medicine "Kamlang Suea Khrong" driven by multiplex PCR and powerful TLC techniques. PLoS One. 2021;16(9): e0257243. https://doi.org/10.1371/journal.pone.0257243 PMID: 34534243
20. Chang F, Yu D, Wang H, Qu S, Wang D, Liu X, et al. Authentication of Saposhnikovia divaricata (Trucz.) Schischk and its two adulterants based on their macroscopic morphology and microscopic characteristics. Microsc Res Tech. 2021;84(5): 1089–1094. https://doi.org/10.1002/jemt.23651 PMID: 33217096
21. Yik MHY, Kong BLH, Siu TY, Lau DTW, Cao H. Differentiation of Hedyotis diffusa and common adulterants based on chloroplast genome sequencing and DNA barcoding markers. Plants. 2021;10(161): 1–12. https://doi.org/10.3390/plants10010161 PMID: 33467716
22. Lo YT, Yik MHY, Shaw PC. Effective authentication of Placenta Hominis. Chin Med. 2018;13(1): 1–8. https://doi.org/10.1186/s13020-018-0188-7 PMID: 29946350
23. Academia Sinica Center for Digital Cultures, Forestry Bureau. Lespedeza cuneata (Dum. Cours.) G. Don. Taiwan Encyclopedia of Life. 2022. Available from: https://taieol.tw/pages/41971.
24. Chen LX, Li N, Zhang M, Zhang CF. Study of the Senecio Scandens Buch-Ham. Strait Pharmaceutical Journal. 2006;18(5): 13–16.
25. Bao BJ, Borsch T, Clemants SE. Achyranthes aspera var. aspera. Flora of China. 2021;5: 1a. Available from: http://www.efloras.org/florataxon.aspx?flora_id=2&taxon_id=242300546.
26. Chen YL, Nordenstam B, Jeffrey C, Leszek V. Senecio scandens Buchanan-Hamilton ex D. Don, Prodr. Fl. Nepal. 178. 1825. Flora of China. 2021;20–21: 510,533. Available from: http://www.efloras.org/florataxon.aspx?flora_id=2&taxon_id=200024485.
27. Yik MHY, Wong VCWT, Wong TH, Shaw PC. HerBChain, a blockchain-based informative platform for quality assurance and quality control of herbal products. J Tradit Complement Med. 2021;11(6): 598–600. https://doi.org/10.1016/j.jtcme.2021.07.005 PMID: 34765524
28. Zhu L, Li N, Ruan JQ, Fu PP, Zhao ZZ, Lin G. Chemical diversity investigation of hepatotoxic pyrrolizidine alkaloids in qianliguang (Senecio scandens) and related species by UHPLC-QTOF-MS. World J Tradit Chin Med. 2015;1(2): 1–11.
29. World Health Organization. Quality control methods for herbal materials. Geneva: WHO Press; 2011. Available from: https://apps.who.int/iris/bitstream/handle/10665/44479/9789241500739_eng.pdf.
30. Li Q, Sun Y, Guo HJ, Sang F, Ma HY, Peng H, et al. Quality control of the traditional Chinese medicine Ruyi jinhuang powder based on high-throughput sequencing and real-time PCR. Sci Rep. 2018;8(1): 1–10. https://doi.org/10.1038/s41598-017-17765-5 PMID: 29311619
31. International Barcode of Life Consortium (iBOL). DNA barcoding—International Barcode of Life. 2022. Available from: https://ibol.org/about/dna-barcoding/.
32. Doebley J, Durbin M, Golenberg EM, Clegg MT, Ma DP. Evolutionary analysis of the large subunit of carboxylase (rbcL) nucleotide sequence among the grasses (Gramineae). Evolution. 1990;44(4): 1097–1108. https://doi.org/10.1111/j.1558-5646.1990.tb03828.x PMID: 28569033
33. Devey DS, Chase MW, Clarkson JJ. A stuttering start to plant DNA barcoding: Microsatellites present a previously overlooked problem in non-coding plastid regions. Taxon. 2009;58(1): 7–15. https://doi.org/10.1002/tax.581003
34. Whitlock BA, Hale AM, Groff PA. Intraspecific inversions pose a challenge for the trnH-psbA plant DNA barcode. PLoS ONE. 2010;5(7): e11533. https://doi.org/10.1371/journal.pone.0011533 PMID: 20644717
35. Han J, Zhu Y, Chen X, Liao B, Yao H, Song J, et al. The short ITS2 sequence serves as an efficient taxonomic sequence tag in comparison with the full-length ITS. BioMed Research International. 2013: 741476. https://doi.org/10.1155/2013/741476 PMID: 23484151
36. Gao T, Yao H, Song J, Zhu Y, Liu C, Chen S. Evaluating the feasibility of using candidate DNA barcodes in discriminating species of the large Asteraceae family. BMC Evolutionary Biology. 2010;10(324): 1–7. https://doi.org/10.1186/1471-2148-10-324 PMID: 20977734
37. Zhang SY, Tang CL, Lin C. Song XL, Liu Y, Zhang E, et al. Determination of seneciphylline in Senecio scandens from different origins based on HPLC-MS/MS. J Anhui Agric Sci. 2022;50(2): 202–205. https://doi.org/10.3969/j.issn.0517-6611.2022.02.055
